# A panel of correlates predicts vaccine-induced protection of rats against respiratory challenge with virulent *Francisella tularensis*

**DOI:** 10.1371/journal.pone.0198140

**Published:** 2018-05-25

**Authors:** Roberto De Pascalis, Andrew Hahn, Helen M. Brook, Patrik Ryden, Nathaniel Donart, Lara Mittereder, Blake Frey, Terry H. Wu, Karen L. Elkins

**Affiliations:** 1 Laboratory of Mucosal Pathogens and Cellular Immunology, Division of Bacterial, Parasitic and Allergenic Products, Center for Biologics Evaluation and Research, U.S. Food and Drug Administration, Silver Spring, Maryland, United States of America; 2 Center for Infectious Disease and Immunity, University of New Mexico, Albuquerque, New Mexico, United States of America; 3 Department of Mathematics and Mathematical Statistics, Umeå University, Umeå, Sweden; 4 Department of Internal Medicine, University of New Mexico, Albuquerque, New Mexico, United States of America; New York Medical College, UNITED STATES

## Abstract

There are no defined correlates of protection for any intracellular pathogen, including the bacterium *Francisella tularensis*, which causes tularemia. Evaluating vaccine efficacy against sporadic diseases like tularemia using field trials is problematic, and therefore alternative strategies to test vaccine candidates like the *Francisella* Live Vaccine Strain (LVS), such as testing in animals and applying correlate measurements, are needed. Recently, we described a promising correlate strategy that predicted the degree of vaccine-induced protection in mice given parenteral challenges, primarily when using an attenuated *Francisella* strain. Here, we demonstrate that using peripheral blood lymphocytes (PBLs) in this approach predicts LVS-mediated protection against respiratory challenge of Fischer 344 rats with fully virulent *F*. *tularensis*, with exceptional sensitivity and specificity. Rats were vaccinated with a panel of LVS-derived vaccines and subsequently given lethal respiratory challenges with Type A *F*. *tularensis*. In parallel, PBLs from vaccinated rats were evaluated for their functional ability to control intramacrophage *Francisella* growth in *in vitro* co-culture assays. PBLs recovered from co-cultures were also evaluated for relative gene expression using a large panel of genes identified in murine studies. *In vitro* control of LVS intramacrophage replication reflected the hierarchy of protection. Further, despite variability between individuals, 22 genes were significantly more up-regulated in PBLs from rats vaccinated with LVS compared to those from rats vaccinated with the variant LVS-R or heat-killed LVS, which were poorly protective. These genes included IFN-γ, IL-21, NOS2, LTA, T-bet, IL-12rβ2, and CCL5. Most importantly, combining quantifications of intramacrophage growth control with 5–7 gene expression levels using multivariate analyses discriminated protected from non-protected individuals with greater than 95% sensitivity and specificity. The results therefore support translation of this approach to non-human primates and people to evaluate new vaccines against *Francisella* and other intracellular pathogens.

## Introduction

Clinical studies of new vaccines can often be facilitated by taking advantage of correlates of protection, but no correlates have been defined for any intracellular pathogen. Protection against most of these pathogens, such as *Francisella tularensis* and *Mycobacterium tuberculosis*, depends heavily on immune responses mediated by T lymphocytes, which can be challenging to measure. In the absence of correlates, clinical trials to evaluate vaccine efficacy require natural exposure of subjects to the infectious agent in regions where the causative agent of the disease is endemic, or through human experimental challenge studies. The low incidence of diseases such as tularemia [1, 2], which is caused by the intracellular bacterium *F*. *tularensis* (*Ft*), makes clinical trials based on natural exposure to microorganisms difficult. On the other hand, human challenge studies are problematic, due to ethical issues and limitations on the number of individuals that can be feasibly enrolled.

To overcome these issues, an alternative strategy dubbed the ‘Animal Rule’ is now available in the U.S. to seek FDA licensure, in which safety is still evaluated by traditional human clinical studies but efficacy may be evaluated by studies using animals [3]. In this context, the use of correlates of vaccine-induced protection may facilitate bridging between species as well as extrapolating expectations of efficacy from animals to humans, particularly if similar measurements can be designed in both animal and human studies. This is especially challenging for intracellular pathogens such as *Ft*.

The candidate vaccine “Live Vaccine Strain,” or LVS, was derived by the U.S. Department of Defense in the 1960’s from a Type B Russian vaccine strain [4], and has been studied under U.S. Investigational New Drug (IND) for decades. LVS is the only *Francisella* vaccine actively being developed through clinical studies in the U.S. [5, 6] but is not licensed; it is given to people by scarification and provides some protection, as demonstrated by human challenge studies in the 1960’s [7], but the extent of protection has not been evaluated in field trials. Inbred mice survive LVS administration up to 10^6^ colony forming units (CFU) when given subcutaneously (s.c.) or intradermally (i.d.). However, low doses of LVS are lethal for mice when given intraperitoneally (i.p.) or intranasally (i.n.), and death ensues within about 5–7 days. Further, vaccination of mice via sublethal i.d. LVS infection results in very strong protection against subsequent lethal i.p. or respiratory LVS challenge [8, 9]. These features provide a convenient small animal model of homologous vaccination and lethal challenge. Further, mouse immune responses to *Francisella* LVS infections are quite similar to those against other intracellular pathogens [8, 10, 11], suggesting that this model is representative of bacteria with an intracellular lifestyle.

Typically, T cell functions are assessed *ex vivo* by re-stimulation of leukocytes and by the quantification of readouts such as cell proliferation and cytokine production. Previously, we have favored an *in vitro* co-culture system in which T cells are re-stimulated via macrophages infected with live bacteria, instead of by re-stimulation with killed bacteria or protein preparations. This approach allows the measurement of number of bacteria, recovered from infected macrophages, to be used as additional readout. To study the role of vaccine-induced T cell responses using this approach, we developed a panel of *Francisella* vaccines that provided different amounts of protection against parenteral (intraperitoneal) lethal challenge of mice with LVS [12]. We then demonstrated that intramacrophage bacterial replication was strongly controlled when LVS-infected macrophages were co-cultured with immune lymphocytes obtained from mice given the strongest vaccines, less controlled using lymphocytes from moderately protective vaccines, and poorly controlled when using lymphocytes from poorly protective vaccines; bacterial replication control was T cell-dependent. Thus, the *in vitro* hierarchy of intramacrophage bacterial growth control exhibits a pattern that directly correlates with vaccines’ *in vivo* efficacy [12]. We further identified potential correlates by screening gene expression in non-adherent mouse lymphocytes, including peripheral blood lymphocytes (PBLs), which were recovered from the co-cultures. Similar to the control of *in vitro* intramacrophage bacterial replication, expression of about 30 genes was differentially regulated according to a pattern that reflected *in vivo* vaccine efficacy [13]. Furthermore, similar expression patterns of a subset of these genes were observed in PBLs from mice given vaccines of different efficacy against *M. tuberculosis [14]*, suggesting similarities between efficacious T cell immune responses against intracellular bacteria [10]. However, no single factor was strongly predictive, which is not surprising given the complexity of T cell mechanisms and biological variabilities between individuals. We [15] and others [16] therefore developed mathematical models that combined data from 2–3 different readouts to provide improved discrimination between vaccine groups.

Tularemia in humans is caused by two biovars of *F*. *tularensis*. Infections with Type A *F*. *tularensis* subsp. *tularensis* are generally more acute with greater mortality than infections with Type B (*F*. *tularensis* subsp. *holarctica*), in which disease may be protracted but rarely deadly [17]. Like humans, but unlike mice, Fischer 344 rats die following low dose Type A *F*. *tularensis* challenge but survive infection with Type B strains and with *F*. *novicida*, another *Francisella* species that rarely causes human disease [18]. Further, rats vaccinated with LVS subcutaneously (s.c.) survive a large intratracheal (i.t.) or aerosol challenge with highly virulent Type A *Ft* SchuS4 [19]. Thus, to date, Fischer 344 rats provide a readily available small, inbred animal model that better approximates the susceptibility of humans to *Ft* than do mice. Here, we applied the approach of coupling *in vitro* control of intramacrophage bacterial growth with measurement of relative gene expression in responding lymphocytes to identify correlates of protection using PBLs from differentially vaccinated rats. Remarkably, using measurements of bacterial growth control combined with relative expression of up to six genes predicted protection of individual animals against respiratory challenge with fully virulent *Ft* with greater than 95% sensitivity and specificity.

## Materials and methods

### Experimental animals

Twelve to sixteen week-old specific-pathogen free (SPF) female inbred Fischer 344 rats (Harlan Laboratories, Indianapolis, IN), were age matched within each experiment. All animals were housed in ventilated cages, and circulating air was HEPA filtered; the room was maintained at negative pressure, under temperature and humidity control and with a 12 hour light on/off cycle. The animals were fed *ad libitum* with irradiated food and autoclaved chlorine dioxide-treated water.

### Ethics statement

All animal studies were approved by the University of New Mexico Health Sciences Center Institutional Animal Care and Use Committee, protocols numbers 12-100792-HSC and 14-101234-TR-HSC. These protocols meet the standards for humane animal care and use set by the 8^th^ Edition of the Guide for the Care and Use of Laboratory Animals and PHS policy.

### Bacteria and growth conditions

*F*. *tularensis* LVS (ATCC 29684) and LVS-R (originally obtained from Dr. Francis Nano, University of Victoria, Canada) [20], were grown to mid-log phase in supplemented Mueller-Hinton broth, and working infection stocks were quality controlled as previously described (Difco Laboratories, Detroit, MI) [9]. Heat killed LVS (HK-LVS) was prepared immediately prior to use by treating LVS at 60°C for 60 minutes; killing was confirmed by plating onto chocolate agar plates (Hardy Diagnostics; Santa Maria, CA) or enriched Mueller-Hinton plates [21]. *F*. *tularensis* strain SchuS4 was originally derived from Master Cell Bank, NR-28534 (BEI Resources, Manassas, VA). The Master Cell Bank was sub-cultured in Modified Cysteine Partial Hydrolysate (MCPH) broth once to produce a sub-master stock and a second time to produce a working stock, which as frozen in aliquots with 20% glycerol at -80°C.

### Bacterial immunization and challenge

For each independent experiment, 7–10 rats were vaccinated for each vaccine group. Rats anaesthetized with isoflurane were vaccinated by s.c. administration of 3 x 10^6^ CFU LVS or LVS-R, or the amount equivalent to 3 x 10^6^ HK-LVS, diluted in 0.1 ml phosphate-buffered saline (PBS) (Fischer/BioWhittaker, Walkersville, MD); control groups received 0.1 ml PBS. Four rats from each group were sacrificed 5–6 weeks after vaccination; blood was collected by cardiac puncture and spleens were isolated. The remaining 3–6 rats were challenged with a lethal dose of 10^3^ *F*. *tularensis subsp*. *tularensis* SchuS4 i.t. and monitored for survival for at least 20 days. Challenges were performed on anesthetized animals using a laryngoscope for visualization of the tracheal opening and to facilitate inoculations. Three extra rats were inoculated with challenge material to use for determination of initial CFU deposited in lungs. The target challenge dose of 10^3^ was selected based on previous experiments which tested survival of naïve rats compared to rats vaccinated solely with LVS. Actual vaccination and challenge doses, as well as CFU in lung homogenates from three control rats sacrificed shortly after challenge, were confirmed by plating on chocolate agar. Challenge experiments were repeated seven times, and a total of 31–37 animals were used for survival studies. To assess the *in vivo* gene expression of PBLs and splenocytes, three rats per vaccine group per time point were immunized as described above and sacrificed at specific time points after vaccination; blood was collected, spleens were isolated, and single-cell suspensions of splenocytes and PBLs were prepared.

### Plasma collection

150 to 500 μL of blood was collected into heparinized tubes from the lateral tail vein prior to vaccination, 2–3 weeks after vaccination, and 2–3 weeks after challenge for surviving animals, and processed for plasma according to standard protocols. Plasma from post-challenge samples was passed through a 0.2 μm syringe filter and plated onto chocolate agar to confirm sterility. Plasma was then stored at -80°C for further analysis.

### Co-culture of LVS infected bone marrow derived macrophages with leukocytes

The protocol previously described for mouse co-cultures [12, 22] was used to culture and infect rat bone marrow derived macrophages (BMMØ) with minor revisions. Briefly, 10^6^ ACK-treated cells from bone marrow were cultured in complete DMEM (DMEM supplemented with 10% heat-inactivated FCS [HyClone, Logan, UT], 18–20% L-929-conditioned medium, 0.2 mM L-glutamine, 10 mM HEPES buffer, 1 mM sodium pyruvate, 1 mM sodium bicarbonate and 0.1 mM nonessential amino acids) in 24 well plates. After 6–7 days of incubation with media changes, confluent macrophage monolayers were infected for 2 hours with *F*. *tularensis* LVS at a multiplicity of infection (MOI) of 1:50 (bacterium-to-BMMØ), washed, treated for 60 min with 50 μg/ml gentamicin, and washed with antibiotic-free medium. Single-cell suspensions of splenic lymphocytes or PBLs derived from vaccinated or naïve rats were generated by standard techniques as previously described [12], and 5 x 10^6^ cells/well added to LVS-infected macrophages. For most co-cultures, PBLs and splenocytes from four animals were pooled; or, in the indicated experiments, splenocytes from individual animals were co-cultured separately. After two days, supernatants from co-cultures were harvested, and non-adherent cells were recovered, pelleted, and stored in RNA*later* at -80°C. Supernatants were also stored at -80°C for further analyses. An aliquot of recovered non-adherent cells was used for surface staining and flow cytometry analysis. LVS replication was determined by lysing infected macrophages and plating, as previously described [12]. Additional co-cultures using splenocytes were harvested after three days for supernatants and determination of bacterial CFU.

### Flow cytometry

Single cell suspensions prepared from blood and spleens, and cells recovered from co-culture after two days, were stained for a panel of rat cell surface markers and analyzed using a Becton-Dickinson LSR II flow cytometer (San Jose, CA) and FlowJo software (Tree Star, Inc.) as previously described [23, 24]. Antibody concentrations were titrated and optimized separately for use in seven-color staining protocols as required. The following antibodies were used: anti-CD45R (clone HIS24), anti-CD45RA (clone OX-33), anti-CD11b/c (clone OX-42), anti-CD3 (clone 1F4), anti-CD8a (clone OX-8), anti-CD4 (clone OX-35), and anti-CD45 (clone OX-1) (all antibodies were purchased from Fischer / BD Biosciences). Live cell discrimination was assessed by using a commercially available Live/Dead Staining Kit (Invitrogen).

### *In vitro* cell purification

A MACS Midi system and anti-CD45R microbeads (Miltenyi Biotec, Auburn, CA) was used to enrich or deplete B cells according to the manufacturer’s protocols. Single cell suspensions of splenocytes obtained from either vaccinated or control rats were treated with the appropriate amount of magnetic beads. The composition and relative purity of the resulting enriched cells were assessed by multiparameter flow cytometry. Enriched B and non-B cells were then used to overlay LVS-infected macrophages in co-culture experiments, as described above.

### Evaluation of IFN-γ and nitric oxide in culture supernatants

Assessment and quantification of rat IFN-γ in supernatants recovered from *in vitro* co-cultures was performed using standard sandwich ELISAs, according to the manufacturer’s instructions (eBioscience, San Diego, CA); IFN-γ was quantified by comparison to recombinant standard proteins (Peprotech, Rocky Hill, NJ). Estimation of nitric oxide was performed in culture supernatants using the Griess reaction (Sigma-Aldrich, St Louis, MO) and quantified by comparison to serially diluted NaNO_2_ [25].

### Real time PCR

Total RNA extraction from samples (RNeasy mini kits, Qiagen, Valencia, CA) and cDNA synthesis (High Capacity RNA-to-cDNA Kit, Applied Biosystems, Carlsbad, CA) were performed following the manufacturer’s instructions, as previously described [12]. RNA purified from cells recovered from co-cultures was used for two separate gene expression analyses. First, a total of 93 genes of immunological interest plus three housekeeping genes, distributed in a custom array, were analyzed using Applied Biosystem primers and probes. The array included all the potential correlates of protection that were identified in PBLs and splenocytes derived from differentially vaccinated C57BL/6J mice [13], as well as additional immune-related mediators identified in similar experiments using *M*. *tuberculosis* [10]. For these quantifications, cDNA was synthesized from pooled PBL samples or from pooled splenocytes. In a second set of analyses, the expression of 10 selected genes was assessed from splenocytes recovered from co-cultures that were established using spleens from individual rats. In addition, RNA purified from PBLs and splenocytes from rats sacrificed at the indicated time points after vaccination, without *ex vivo* re-stimulation, was used to amplify twenty-two selected genes. In all qRT-PCR analyses, GAPDH, RPS29, and GUSB were used to normalize the data; delta Ct (ΔCt) and the ratios between ΔCt of vaccine samples and control naïve samples were then calculated. All semi-quantitative real-time PCR analyses were performed with a ViiA 7 sequence detection system (Applied Biosystems).

### Serum analyses

Specific anti-LVS serum IgG and IgM were determined by ELISA as previously described [26] with modifications. Briefly, vinyl “U” plates (ThermoScientific) were coated with 5 x 10^6^ LVS bacteria per well diluted in coating buffer (4.2 mg/L NaCO_3_, pH 9) and incubated for 2 hours at 37°C, then overnight at 4°C. Wells were then washed with PBS supplemented with 0.05% Tween 20 (PBST) and blocked with 10% HiPure Liquid Gelatin (Norland Products, Cranbury, NJ) in PBS for 60 minutes at 37°C. After washing, serum samples were serially diluted in blocking buffer and added to each well and incubated for 90 minutes at 37°C. The plates were washed with PBST and incubated with HRPO-labelled antibodies detecting rat IgM and total rat IgG (Southern Biotech, Birmingham, AL) for 90 minutes at 37°C. After washing, the assay was developed by the addition of TMB substrate solution (Kirkegaard & Perry Laboratories, Gaithersburg, MD) for 15 minutes at room temperature and stopped with the addition of 0.5N H_2_SO_4_. Optical density was read at 410 nm. Blank OD values, generated by developing wells that included all reagents without serum samples, were subtracted from sample values. A negative cut-off value was determined by averaging OD values from wells containing sera from PBS-vaccinated rats and then adding 3 standard deviations of the mean. The endpoint titer of a given sample was defined as the reciprocal of the last dilution with an OD value greater than that of the cut-off value [26–28].

### Statistical analyses

**Fig 1:**
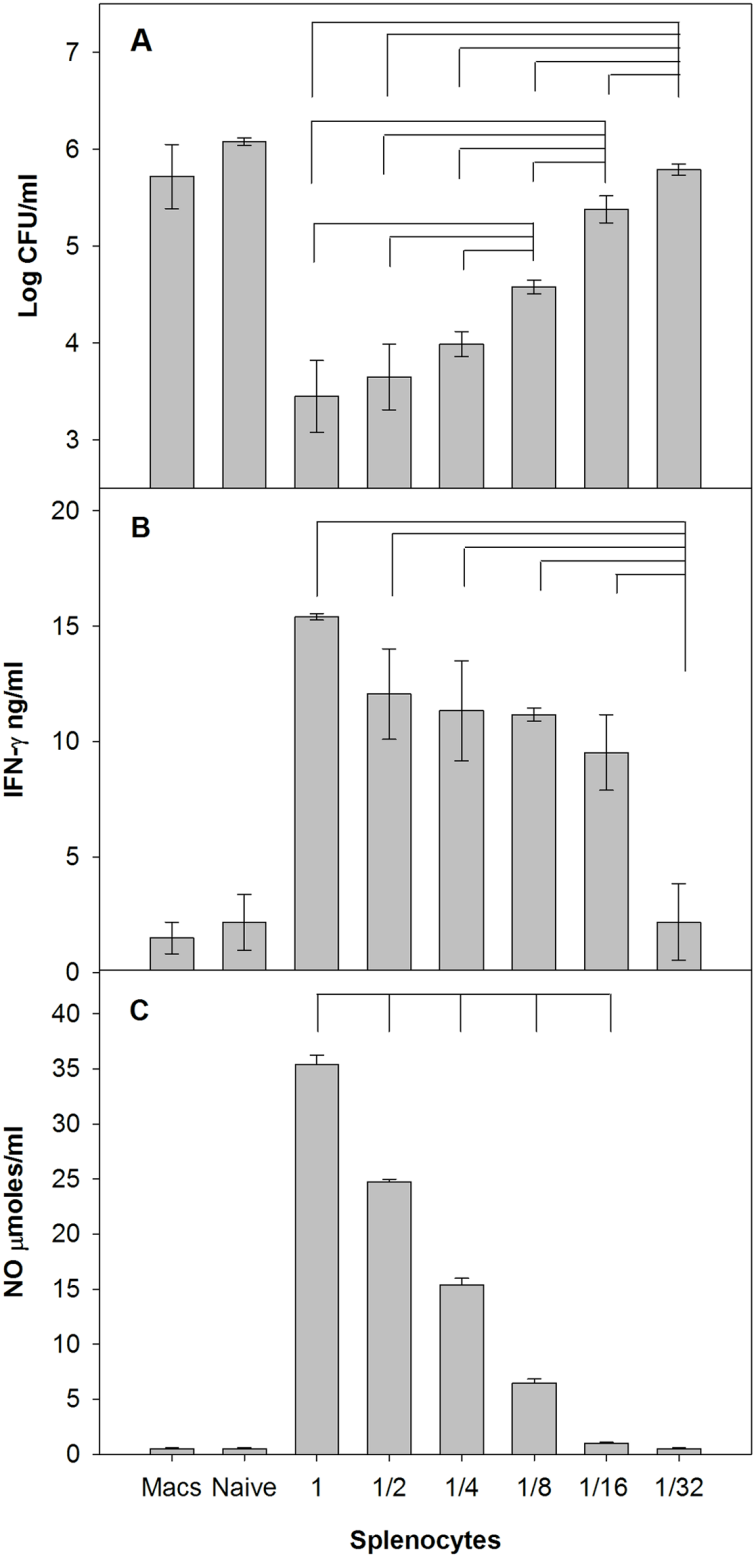
Rat splenocytes control intramacrophage *Francisella* growth that is accompanied by IFN-γ and NO production. BMMΦ from Fischer 344 rats were infected with LVS (Macs), and co-cultured with naïve splenocytes or with immune splenocytes derived from LVS-vaccinated rats. Decreasing numbers of splenocytes were added to a constant number of confluent LVS-infected macrophages, where 1 = 5 x 10^6^, 1/2 = 2.5 x 10^6^, 1/4 = 1.25 x 10^6^, 1/8 = 0.625 x 10^6^, 1/16 = 0.312 x 10^6^, 1/32 = 0.156 x 10^6^ splenocytes. After three days of co-culture, BMMΦ were lysed to evaluate the recovery of intracellular bacteria (Panel A). Values shown are the mean numbers of CFU/ml ± SD of viable bacteria from triplicate wells. Supernatants were collected for analyses of IFN-γ by ELISA (Panel B) and NO by Griess reaction (Panel C), and concentrations of each were calculated using standard curves as references. Values shown are the mean concentration ± standard deviation of triplicate wells. Brackets indicate a significant difference (*P* < 0.05) between the recoveries of bacteria from macrophages in co-cultures or production of IFN-γ and nitric oxide.

**Fig 2:**
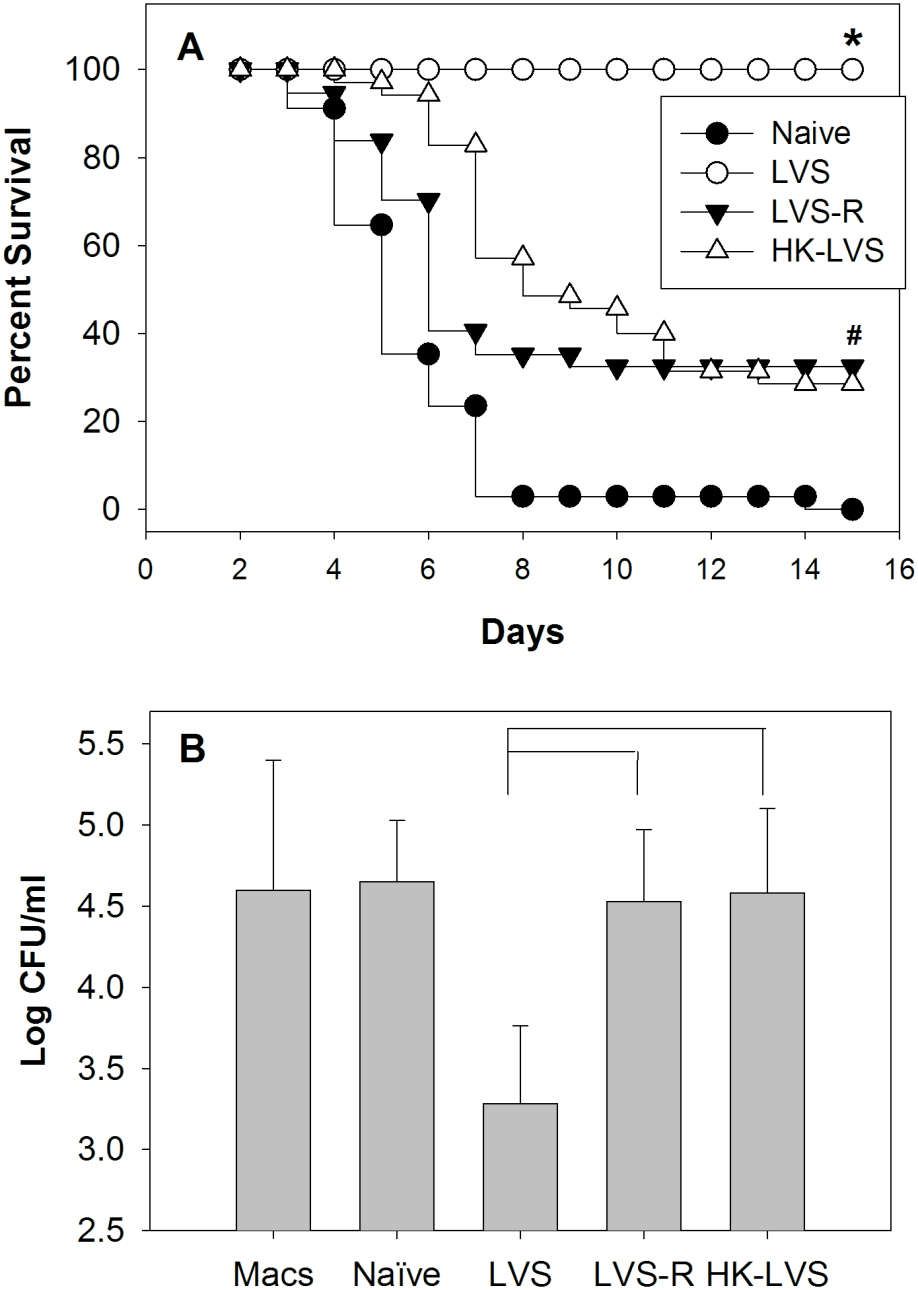
Rat PBLs from differentially vaccinated rats control intramacrophage *Francisella* growth in a pattern that reflects vaccine efficacy. Groups of 6 Fischer 344 rats were vaccinated with LVS, LVS-R, HK-LVS, or PBS (naïve) for a total of 31–37 rats per group, and challenged 5 weeks later i.t. with 10^3^ CFU *F*. *tularensis*. Percent survival after one month is shown with data combined from five independent challenge experiments (Panel A). * indicates significantly greater survival compared to naïve rats or rats vaccinated with LVS-R and HK-LVS; # indicates significantly greater survival compared to naïve rats. There was no significant difference between survival of LVS-R and HK-LVS vaccinated rats. In parallel, BMMΦ from Fischer 344 rats were infected with LVS (Macs alone), and co-cultured with PBLs obtained 5 weeks after vaccination from 3 remaining naïve or vaccinated Fischer 344 rats, as indicated. After two days of co-culture, BMMΦ were lysed to evaluate the recovery of intracellular bacteria. Values shown are the mean numbers of CFU/ml ± SD of viable bacteria for five independent experiments of similar design and outcomes (Panel B). Brackets indicate a significant difference (*P* < 0.05) between the recoveries of bacteria from macrophages in co-cultures. There was no significant difference between the recoveries of bacteria from co-cultures using LVS-R or HK-LVS-immune PBLs.

**Fig 3:**
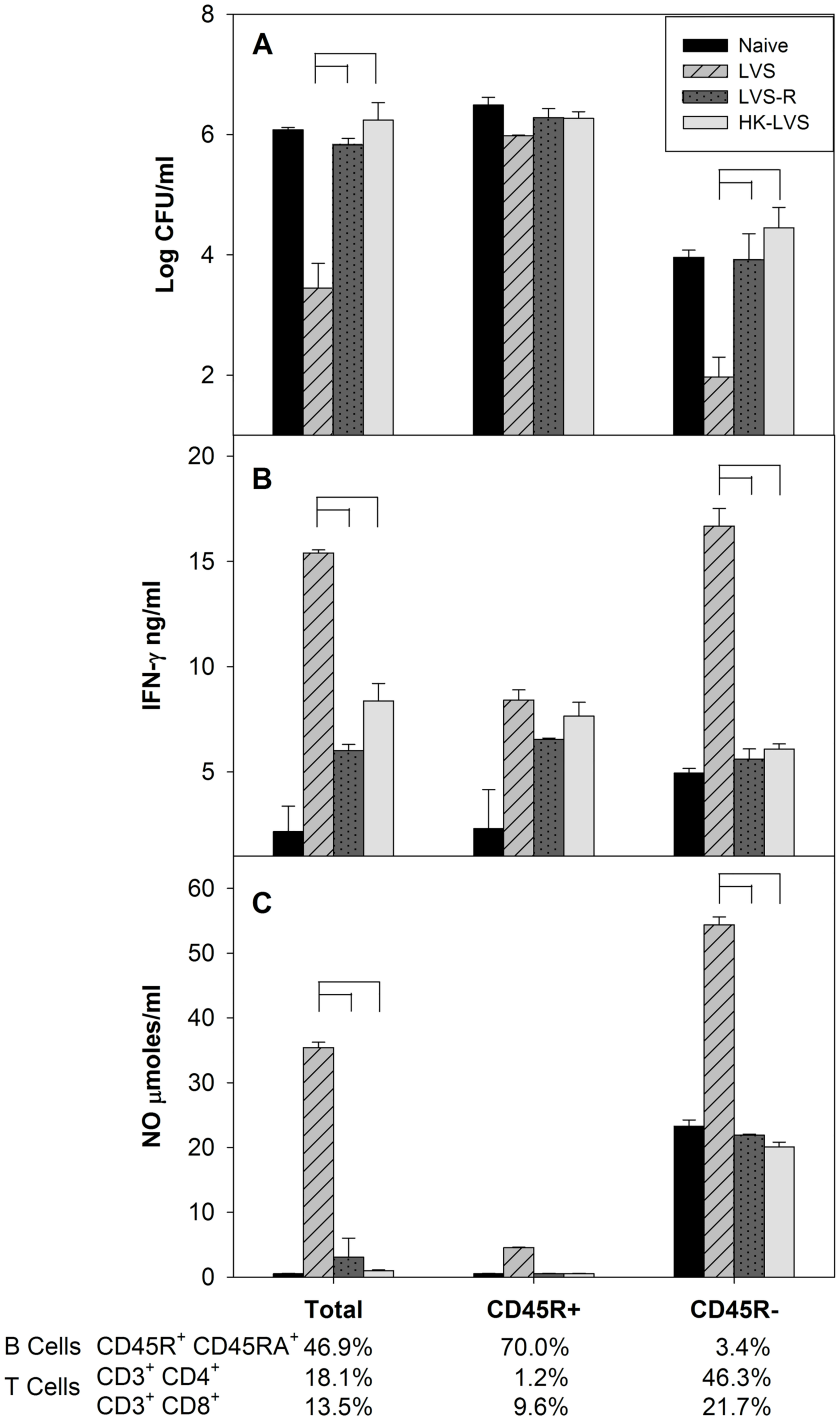
Control of intramacrophage LVS growth depends on T cells but not B cells. BMMΦ from Fischer 344 rats were infected with LVS and co-cultured with total, CD45R^+^ (B lymphocytes) or CD45R^-^ splenocytes (non-B lymphocytes) obtained from naïve or vaccinated Fischer 344 rats, as indicated. Splenocytes were analyzed by flow cytometry to evaluate purity of B and T cells, as indicated. After three days of co-culture, BMMΦ were lysed to evaluate the recovery of intracellular bacteria (Panel A). Supernatants were collected for analyses of IFN-γ by ELISA (Panel B) and NO by Griess reaction (Panel C), and their concentrations were calculated using standard curves as references. Values shown are the mean concentration ± standard deviation of triplicate wells. Brackets indicate significant differences between the indicated groups (*P* < 0.05). Data shown are from one representative experiments of two of similar design and outcome.

**Fig 4:**
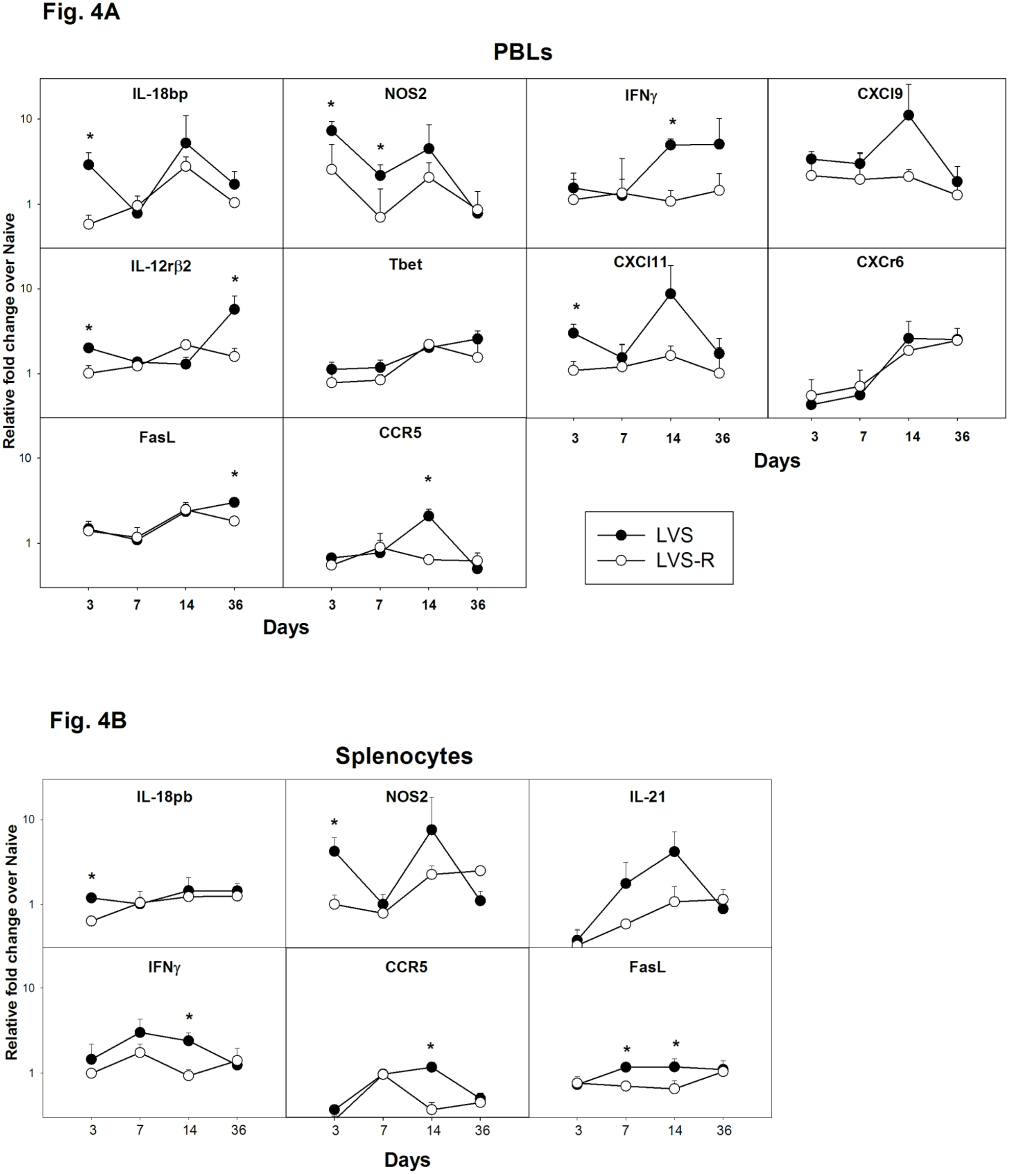
Gene expression of potential correlates of protection is differentially up-regulated in rat PBLs and splenocytes after vaccination with LVS-derived vaccines. Total RNA was purified from PBLs (Panel A) or splenocytes (Panel B) obtained at the indicated time points after vaccination from three naïve rats or from three rats immunized with LVS or LVS-R. RNA was used for semi-quantitative analyses of gene expression using the indicated sets of primers/probes. Values shown are average fold changes ± standard deviation of the indicated genes compared to those from naïve rats. Asterisks indicate significant differences in gene expression (*P* < 0.05) between LVS- and LVS-R-derived cells.

**Fig 5:**
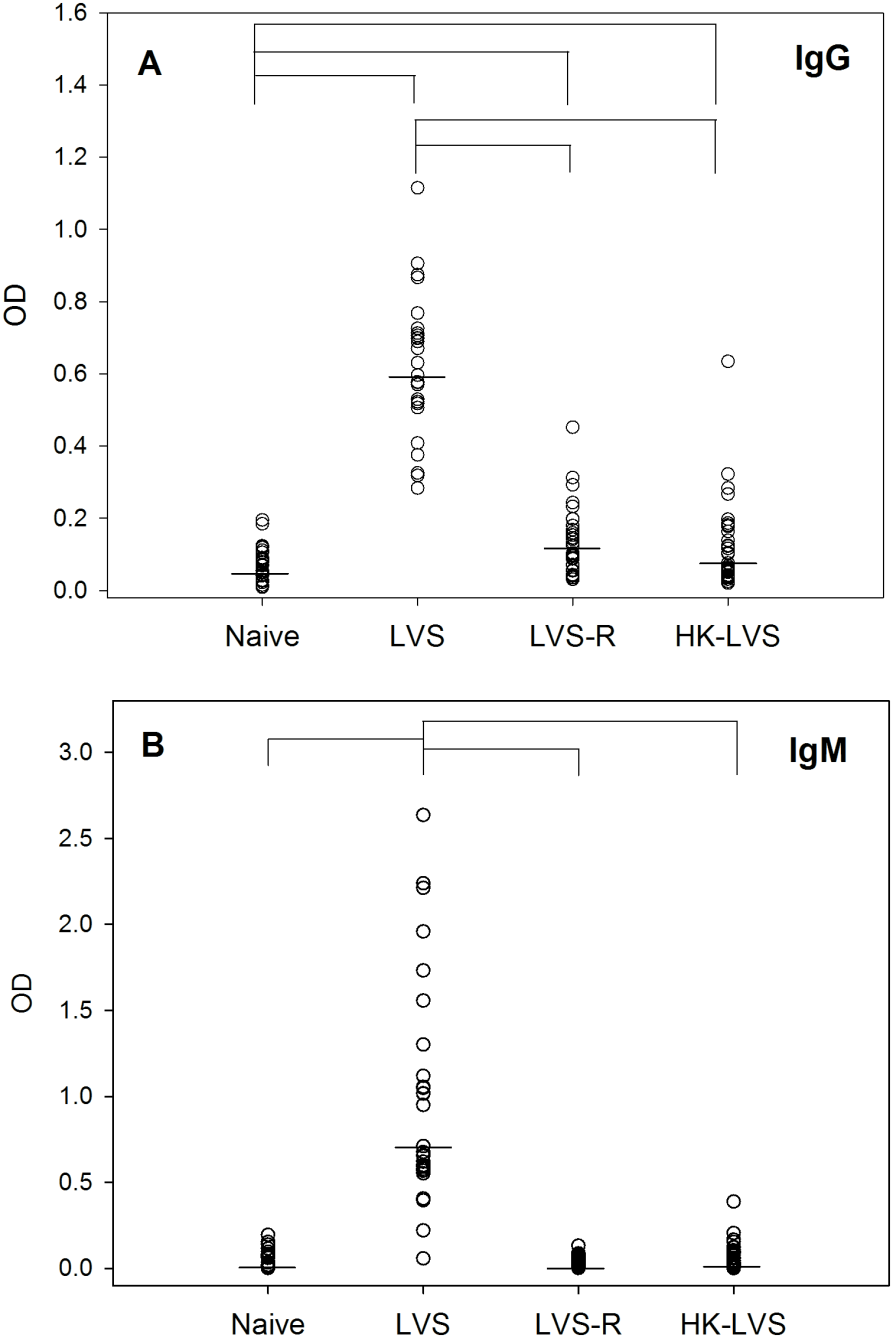
LVS-vaccinated rats exhibit humoral immune responses. Sera from individual rats were obtained 2–3 weeks after vaccination and analyzed for anti-LVS total IgG (Panel A) and IgM (Panel B) antibodies. Sera from four sets of vaccinations were tested for a total of 27–35 sera for each vaccine group. Shown are the OD values obtained at a 1:1600 dilution for each individual serum; lines indicate median values for the group. Brackets indicate significant differences (*P* < 0.05) between the indicated antibody responses.

Statistical analyses to evaluate differential bacterial growth, cytokine production, gene expression in recovered leukocytes, and *in vivo* gene expression from PBLs (without re-stimulation), were performed using Microsoft Excel. CFU data were log_10_ transformed, and cytokine and nitric oxide concentrations were measured in a log scale; thus, a normal distribution was assumed. Significant differences in CFU levels (Figs 1A, 2B and 3A), cytokine (Figs 1B and 3B) and nitric oxide levels (Figs 1C and 3C), gene regulation for individual pairs of means (Fig 4), and humoral immune responses (Fig 5) were evaluated using a two-tailed Student’s *t* test, with a *P* value of < 0.05 indicating significance. Corrections for multiple comparisons, as seen in Figs 1, 2B, 3 and 5, were performed using the Bonferroni method. Significant differences in survival after challenge (Fig 2A) were evaluated using log- rank test, with a *P* value of < 0.05 indicating significance.

Linear discriminant analysis (LDA), assuming a homoscedastic covariance structure and using a non-informative prior, was used to determine a decision rule with the objective of discriminating between individuals vaccinated with LVS and all other individuals that were either naïve, vaccinated with LVS-R, or vaccinated with HK-LVS. For this purpose, 2047 experimental models were considered. Each model included between one and eleven variables in all possible combinations, where the 11 variables consisted of average CFU values and ΔCt values from 10 genes. Each model was fitted considering the data from the 88 individuals (22 vaccinated with LVS and 66 other vaccines or naïve, studied in 6–8 separate experiments). Leave one-out cross validation was used to calculate the posterior probabilities of the individuals being classified as LVS-vaccinated, denoted PP-LVS-values. To determine the models’ performance, sensitivity, specificity, and the expected fraction of correctly classified (CC) individuals were calculated.

## Results

### Rat T cell control of intramacrophage *Francisella* growth

To measure *in vitro* T cell function from vaccinated Fischer 344 rats, we first adapted an *in vitro* co-culture system previously used for mouse T cell functional studies for use with rat cells. Rat BMMΦ were infected with *Francisella* LVS, which serves as a BSL-2 surrogate for virulent *Francisella* infection [12, 22, 29]. Either naïve rat splenocytes or splenocytes from LVS-vaccinated rats were then overlaid on the adherent infected macrophages. Three days later, supernatants were recovered, and remaining adherent infected macrophages were lysed to determine bacterial CFU. Addition of an optimal number of LVS-immune splenocytes to confluent LVS-infected macrophages resulted in recovery of bacterial CFU that were approximately 2.5 logs lower than numbers of CFU recovered from co-cultures with splenocytes from naïve animals (Fig 1A). Control of intramacrophage bacterial growth was reduced when the number of added LVS-immune splenocytes was decreased. Control was accompanied by production of IFN-γ (Fig 1B) and nitric oxide (NO; Fig 1C) in co-culture supernatants, and amounts of each were similarly reduced as numbers of splenocytes decreased.

### *In vivo* protection against *Ft* compared to *in vitro* T cell functions

We previously established a panel of vaccines related to LVS, the most efficacious candidate vaccine currently available, that provided strong, moderate, or weak protection to C57BL/6J mice against lethal *Francisella* LVS intraperitoneal parenteral challenge [13]. Here, we evaluated the degree of protection provided by this panel of vaccines in Fischer 344 rats given a respiratory challenge with highly virulent *Ft*. Rats were vaccinated s.c. with LVS, with the opacity variant LVS-R, or with heat killed (HK-) LVS, and then challenged 5–6 weeks after vaccination via i.t. administration of *Ft*. All rats vaccinated with LVS survived challenge, while all naïve rats succumbed; relatively weak protection was observed in rats vaccinated with either LVS-R or HK-LVS (Fig 2A), whose survival was significantly smaller than that of LVS vaccinate rats, but significantly greater than control naïve rats.

To determine whether *in vitro* functions reflect *in vivo* protection, the activities of PBLs (Fig 2B) and splenocytes (Fig 3A) prepared from vaccinated and naive rats were compared using *in vitro* co-cultures in parallel with challenge studies. Leukocytes from LVS-vaccinated animals strongly controlled intramacrophage LVS growth, whereas those from naïve rats did not. PBLs from both LVS-R and HK-LVS vaccinated rats exhibited minimal control of intramacrophage LVS replication that was similar to that of naïve leukocytes. Of note, analyses of the distribution of input leucocytes did not reveal any obvious differences between cell populations from naïve and vaccinated mice by flow cytometry. However, the cell composition of PBLs and splenocytes was somewhat different; CD4 and CD8 T cells were more abundant in PBLs than splenocytes (Panel A in S1 Fig). After two days of co-culture, approximately 20–40% of the input number of splenocytes were recovered from all groups; of those, about 30–40% were live cells represented mostly by B cells and T cells, and the numbers and proportions of non-B or non-T cells were notably reduced compared to numbers found in the input cells (Panel B in S1 Fig).

To determine the cell types responsible for controlling intramacrophage *Francisella* growth, we performed co-cultures using splenocytes enriched for, or depleted of, B lymphocytes (Fig 3). When immune splenocytes used in co-cultures were enriched for CD45R^+^ B cells, resulting in loss of almost all CD4^+^ T cells and a large reduction in CD8^+^ T cells, control of intramacrophage LVS replication was lost in all vaccine groups, and numbers of CFU recovered were similar to those using naïve splenocytes (Fig 3A). In contrast, CD45R^-^ splenocytes, comprised mostly of T cells but almost no B cells, controlled intramacrophage bacterial replication even better than total splenocytes (Fig 3A). Control was generally accompanied by IFN-γ (Fig 3B) and NO (Fig 3C) production; notably, however, supernatants from co-cultures containing mostly B cells contained substantial amounts of IFN-γ, even though these co-cultures did not exhibit control of intramacrophage bacterial growth (Fig 3B).

### Relative gene expression by *Francisella*-immune rat lymphocytes as potential correlates of protection

Previously we evaluated relative gene expression in non-adherent lymphocytes as a complementary readout for T cell functions, and identified a panel of genes whose relative expression in immune mouse lymphocytes reflected the hierarchy of *in vivo* protection of mice against parenteral LVS challenge [12, 13, 15]. To evaluate potential correlates of protection in lymphocytes from vaccinated rats, non-adherent pooled splenocytes or PBLs were recovered from co-cultures and analyzed for expression of a panel of immune-related genes. Table 1 lists results from the 22 genes that were differentially expressed among vaccine groups, with the pattern reflecting *in vivo* protection of LVS >> LVR-R ≅ HK-LVS ≥ naïve, and for which results were consistent across experiments. Most of these genes were differentially upregulated in both PBLs and splenocytes. Moreover, eighteen of these genes were previously identified in mouse studies [12, 13], including IFN-γ, NOS-2, IL-21, T-bet, FasL, and LTA (Table 1, Group 1). Four more genes that were differentially expressed only in rat leukocytes were also identified (Table 1, Group 2).

**Table 1:** Relative gene expression of potential correlates of protection in co-cultures using immune leukocytes from differentially vaccinated rats.

		PBLs			Spleens		
		LVS	LVS-R	HK-LVS	LVS	LVS-R	HK-LVS
Group 1	NOS2	17.61	2.08	2.24	3.34	0.64	1.05
	IFN-γ	9.02	1.84	1.19	8.39	1.22	1.34
	IL-21	5.94	1.48	0.93	10.42	0.82	1.94
	LTA	4.38	1.22	1.35	4.99	1.16	1.14
	FasL	3.80	1.28	1.24	1.73	1.38	0.71
	IL-2ra	2.52	0.97	1.07	2.59	1.05	1.06
	T-bet	2.23	0.87	0.85	1.74	0.83	0.90
	HMOX1	1.98	0.89	1.07	1.51	0.50	0.58
	GzmB	3.39	1.70	1.17	3.49	0.73	0.78
	ICOS	2.05	1.07	1.53	2.34	1.38	1.16
	CCR5	1.94	1.03	0.94	1.90	1.11	0.94
	IL-12rβ2	2.69	1.46	1.21	2.33	0.88	0.89
	SOCS-1	3.95	1.21	0.98	3.89	1.15	1.20
	IL-18bp	2.83	1.10	1.16	2.06	0.82	1.09
	IRF1	2.81	1.25	0.89	2.54	1.38	0.92
	CCL5	4.02	1.54	0.96			
	CCL7	2.10	0.93	1.12			
	CXCR6	1.81	0.78	0.73			
Group 2	CCR3	2.69	0.77	1.41	2.52	0.68	1.56
	CXCL9	5.49	1.64	1.41	3.46	1.28	1.24
	CCL4	4.60	1.93	0.93			
	IL-1RA	2.62	1.14	1.12			

A custom array for real-time PCR was designed by including approximately 30 potential correlates of protection that were identified in PBLs of differentially vaccinated C57BL/6J mice as well as an additional 60 immune-related factors identified in similar studies with *M. tuberculosis* [13, 14]. Shown is the median fold change of the indicated gene compared to naive cells, calculated from 4–6 independent experiments. Group 1 indicates genes exhibiting an expression hierarchy such that LVS >> LVS-R ≅ HK-LVS > naïve and that were previously identified using the mouse model. Group 2 indicate genes exhibiting an expression hierarchy that were uniquely differentially expressed in rat leukocytes but not mouse leukocytes [12, 13].

To determine whether these genes were differentially expressed among vaccine groups in PBLs or splenocytes shortly after vaccination, leukocytes were analyzed without *ex vivo* re-stimulation. Rats were vaccinated with LVS or PBS, sacrificed on days 2, 4, 7, 14, 28, and 56, and blood and splenocytes processed immediately for mRNA analyses of selected genes. Four time points were then selected based on these initial studies. In the next series, rats were vaccinated with LVS, LVS-R, or PBS, and leukocytes obtained over time from differentially vaccinated animals and then analyzed (Fig 4). Several genes were differentially up-regulated in both PBLs (Fig 4A) and splenocytes (Fig 4B) from LVS-vaccinated rats compared to leukocytes from LVS-R-vaccinated rats, including IL-18bp, NOS2, IFN-γ, and FasL. The differential expression patterns shown were consistent in multiple animals, although differences were not always significant. Compared to PBLs, quantitative differences in upregulation of genes in splenocytes were smaller, fewer genes were up-regulated, and none were up-regulated by 36 days after vaccination. Nonetheless, these results suggest that differential gene expression in blood samples may provide another predictive correlate measurement that does not depend on *ex vivo* lymphocyte re-stimulation.

### Assessment of humoral immune responses

To evaluate whether humoral immune responses were associated with survival of challenge with *Ft*, sera were obtained 2–3 weeks after vaccination and analyzed for anti-*Francisella* IgM and IgG antibodies. Strong antibody responses of both isotypes were observed following vaccination with LVS (Fig 5 and S1 Table). In contrast, humoral IgG immune responses following immunization with LVS-R and HK-LVS were minimal, and IgM responses were negligible. Of note, levels of specific antibody responses were highly variable between animals within any given vaccine group, and there was no obvious relationship between individual serum anti-*Francisella* responses and survival after challenge.

### Multivariate analyses to develop predictive models

**Fig 6:**
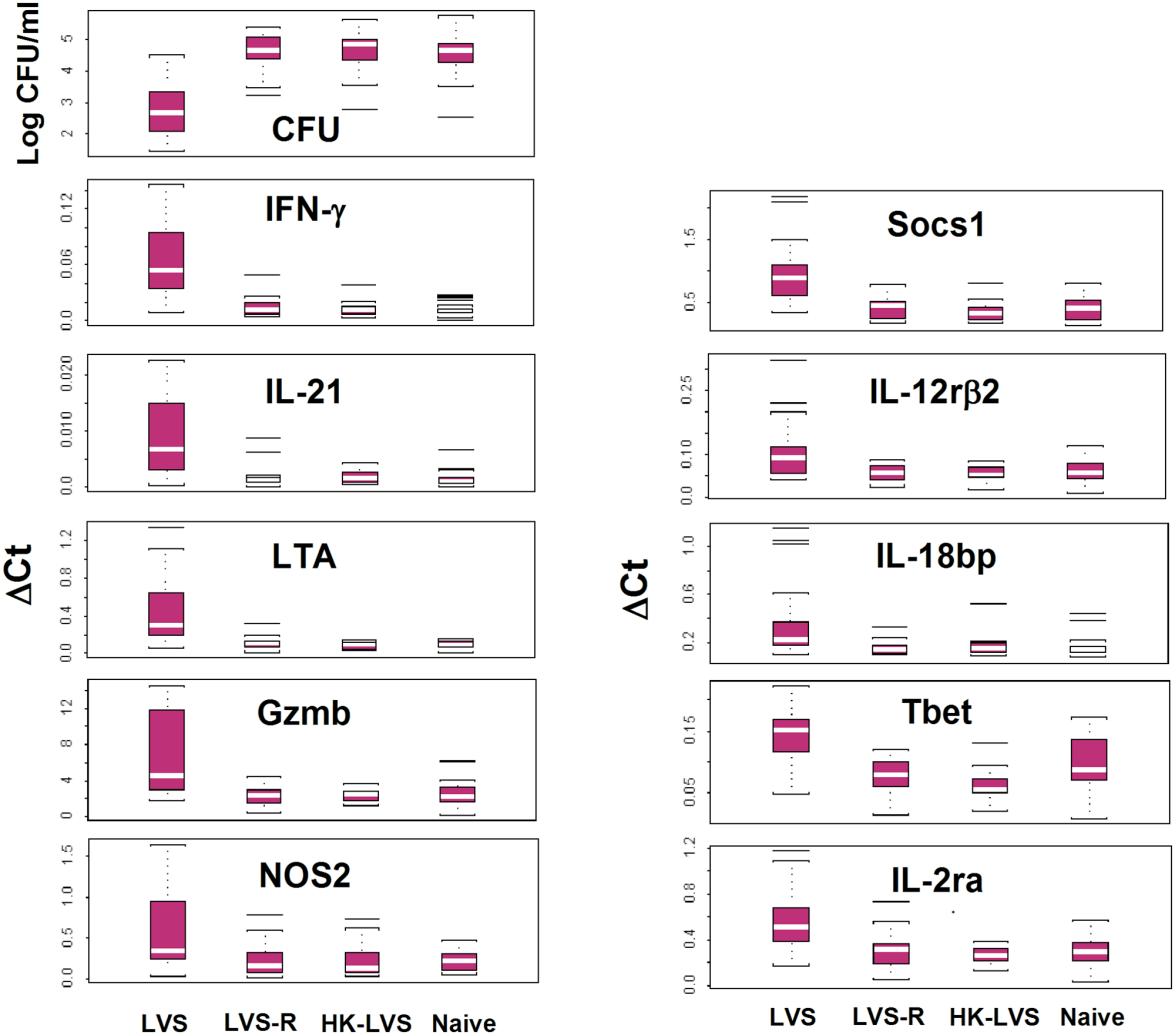
Bacterial replication measurements and relative gene expression measurements discriminate LVS-vaccinated rats from LVS-R-vaccinated, HK-LVS vaccinated, and naïve rats. BMMΦ from Fischer 344 rats were infected with LVS and co-cultured with splenocytes obtained from naïve Fischer 344 rats or rats vaccinated with LVS, LVS-R, or HK-LVS. Splenocytes from 22 rats from each group were analyzed individually in studies comprised of 6–8 separate experiments. After two days of co-culture, splenocytes were recovered and used to purify total RNA, then BMMΦ were lysed to evaluate the recovery of intracellular bacteria. Semi-quantitative gene expression analyses of total RNA from recovered splenocytes were performed using the indicated sets of primers/probes, chosen among those that best reflected the hierarchy of *in vivo* efficacy. Values shown indicate median (white line), 1^st^ and 3^rd^ quartiles (lower and upper box), and maximum and minimum values (upper and lower line). Bacterial growth and gene expression were significantly different for each variable in splenocytes after vaccination with LVS compared to that of naïve, LVS-R, and HK-LVS groups (p < 0.05).

Because relative gene expression measurements yielded a promising correlate option, we evaluated the variability of gene expression within each vaccine group. We selected ten genes that best reflected the hierarchy of vaccine efficacy in rats (LVS >> LVR-R ≅ HK-LVS ≥ naïve). The relative expression of this panel was further analyzed by qRT-PCR in splenocytes recovered from co-cultures established with cells from individual animals. The resulting CFU values and ΔCt values of each individual PCR amplification, illustrated in heat map form (S2 Fig), indicated a clear difference between the LVS-vaccinated group and the other vaccines. However, samples from individual rats from all groups exhibited a range of values that reflected biological variability even in inbred animals. These data were therefore subjected to rigorous statistical analyses (Fig 6). Despite variability, the median levels of CFU and all ten selected genes using splenocytes from the LVS group were significantly different from levels using splenocytes from the LVS-R group, HK-LVS group, or the naïve (PBS) group. In some cases, however, quantitative differences were relatively small, with overlap in absolute values between LVS and non-LVS groups; therefore, no single parameter readily predicted protection of any given individual.

**Fig 7:**
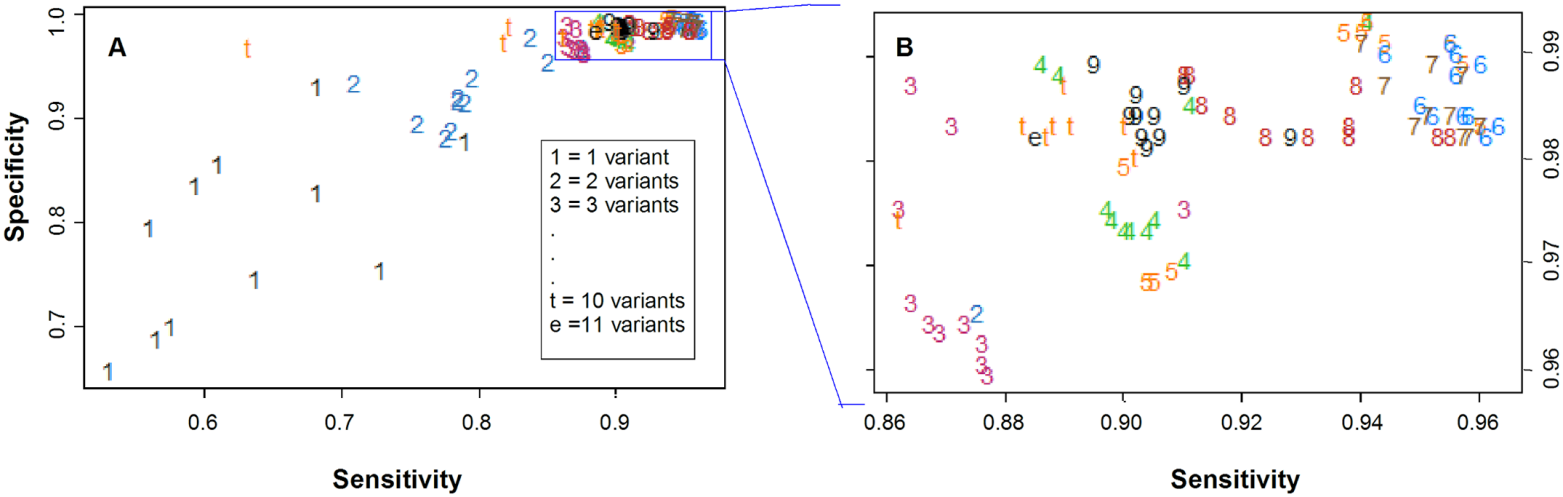
Sensitivity and specificity increase by using groups of 5–7 variables. Eleven variables (CFU and ΔCt data) from the complete data set were used alone or in multiple combinations to generate 2047 experimental models; each model therefore included between 1 and 11 variables. The sensitivity and specificity for a subset of all the models were calculated and plotted (Panel A). This subset was generated by dividing the experimental models into eleven groups according to the number of variables used, and by selecting the top eleven scorers for each group. Plots using numbers or letters indicate the number of variables used in each model, where t and e correspond to 10 and 11 variables, respectively. The area within the rectangle in the upper right hand corner of Panel A corresponds to the highest values of sensitivity and specificity and is enlarged for presentation in Panel B.

The CFU values and ΔCt values were therefore further evaluated using multivariate analyses. To determine whether groups of parameters could be used to discriminate LVS from non-LVS vaccinated animals, sensitivity, specificity, and correctly classified values were calculated from 2047 models that were built by using these 11 variables (S2 Table). Each model consisted of CFU (1) and gene expression values (10), alone or in all possible combinations. The expected number of correctly classified (*i*.*e*., Correct Classification (CC)) ranged between 59.1% and 83.1% when any one of the 11 variables were used alone, with CFU as the highest scorer (S3 Table). CC increased substantially, however, when two (S3 Table) or more (Table 2, S3 Table) variables were combined in the models. The highest CC values were obtained when 5–7 variables were used (Table 2, S3 Table). Correspondingly, sensitivity and specificity were moderate when 1 or 2 variables were used (Fig 7A), but increased dramatically when three or more variables were used (Fig 7B). Different combinations of 5–7 variables achieved > 95% sensitivity and > 98% specificity that did not increase further when additional variables were added (Table 2, S3 Table, Fig 7B). Taken together, these data indicate that a manageable number of measurements used in combination provide excellent discrimination of highly protected individual rats from those with poor or no protection.

**Table 2:** Sensitivity, specificity, and correct classification for linear discrimination analyses.

Number of Variables	Variables	Sensitivity	Specificity	Correct Classification
1	CFU	78.9	87.4	83.1
2	CFU, SOCS1	87.5	96.5	92.0
3	CFU, SOCS1, T-Bet	91.0	97.5	94.2
4	CFU, IFN-γ, LTA, SOCS1	94.1	99.3	96.7
5	CFU, IFN-γ, LTA, SOCS1, IL-2ra	95.5	99.1	97.3
6	CFU, IFN-γ, LTA, NOS2, SOCS1, IL-2ra	96.0	98.9	97.4
7	CFU, IFN-γ, LTA, NOS2, SOCS1, IL-2rβ2, IL-18bp	95.7	98.8	97.2
8	CFU, IFN-γ, LTA, GZMB, NOS2, SOCS1, IL-12rβ2, IL-18bp	95.5	98.2	96.8
9	CFU, IFN-γ, GZMB, NOS2, SOCS1, IL-12rβ2, IL-2ra, IL-18bp	92.8	98.2	95.5
10	CFU, IFN-γ, IL21, LTA, GZMB, NOS2, SOCS1, IL-12rβ2, IL-2ra, IL-18bp	90.0	98.3	94.1
11	CFU, IFN-γ, IL-21, LTA, GZMB, NOS2, SOCS1, IL-12rβ2, IL-2ra, IL-18bp, T-Bet	88.5	98.2	93.3

Experimental models were analyzed using different number of variables. Shown are the best performing models for each combination of 1 to 11 variables. Sensitivity and specificity were calculated by discriminating individuals vaccinated with LVS from naïve as well as from individuals vaccinated with LVS-R or HK-LVS. The expected numbers of correctly classified individual (corrected classification) values are shown, calculated as an average of sensitivity and specificity.

## Discussion

Animal models are widely used to evaluate immune responses against infectious diseases, to explore mechanism of protection, and to obtain preliminary assessments of safety and efficacy of new vaccine candidates. Ultimately, vaccine safety and efficacy are usually evaluated in human clinical trials. However, large clinical studies to determine efficacy may be impractical for infectious diseases such as tularemia that have a low incidence rate in nature. In these circumstances, animal models may facilitate selection and evaluation of potential vaccine candidates, particularly if correlates of protection are available to bridge between species and to aid licensure via the FDA “Animal Rule” [3]. The lack of defined correlates for intracellular pathogens impedes development of vaccines against this entire class of microbes, many of which are significant public health concerns.

We previously defined a strategy that identified potential correlates of protection against parenteral challenge with the attenuated *Francisella* LVS strain by using a mouse model. Because Fischer 344 rats now provide a considerably improved small animal model that better approximates human *Francisella* infection and immunity [18, 19], the overall goals of this study were to evaluate this approach to advance correlate development. Specifically, we wished to determine whether the co-culture method used to identify correlates of protection in mice is applicable to vaccination of Fischer 344 rats subjected to respiratory challenge with highly virulent *Ft*, which has not been studied in previous correlate analyses, and if possible to identify common interspecies correlates of protection against *Ft*.

The subcutaneous route of vaccination was chosen to approximate skin vaccination used in humans [6], as well as to compare findings with mice. Animals were challenged with *Ft* SchuS4 intratracheally to simulate the most virulent form of human infection, which is the form of bioterror concern. The pattern of protection provided by LVS-derived vaccines was similar to, but not the same as, that observed in C57BL/6J mice [12]. As expected, all rats vaccinated with LVS survived challenge, whereas naïve rats succumbed (Fig 2A). In contrast to mice, however, an average of approximately 30% of rats vaccinated with either of the suboptimal vaccines LVS-R and HK-LVS survived this dose of i.t. *Ft* challenge. Survival rates using these two suboptimal vaccines varied across experiments, however; for example, in two of seven experiments, none of the LVS-R-vaccinated rats survived. Because of this pattern, we focused on identifying functional assays and biomarkers which follow the pattern of LVS >> LVS-R ≅ HK-LVS ≥ naïve, reflecting *in vivo* efficacy.

T cell functions of PBLs and splenocytes from rats given different vaccines were tested using *in vitro* co-cultures previously described [12, 13, 15], successfully modified here for use with rat cells. Of note, larger numbers of T cells were found in rat PBLs than in rat spleens, and correspondingly more T cells were later recovered from PBL co-cultures. It is not clear whether T cells proliferate during co-cultures, but it is likely that activated lymphocytes survive longer than other cell types, thereby contributing to their relative enrichment. Similar to mouse studies [13], PBLs and splenocytes from LVS-vaccinated rats were re-stimulated efficiently *in vitro* by interaction with LVS-infected macrophages to effect control of intramacrophage bacterial replication. Control was a function of the number of lymphocytes added to co-cultures (Figs 1, 2B and 3). Although enrichment and depletion of B cells was suboptimal with the available reagents, results clearly indicated that *in vitro* control of LVS intramacrophage replication is T cell-dependent but B cell-independent (Fig 3). Production of nitric oxide was associated with strong control (Fig 3C). However, co-culture supernatants when using cells from LVS-R- or HK-LVS-vaccinated rats, or with B cell-enriched cells that lacked control, contained substantial amounts of IFN-γ (Fig 3B). These observations are consistent with the concept that IFN-γ is necessary, but not sufficient, during responses to intracellular bacteria such as *Francisella* [10, 30–33].

In the mouse model, LVS-R provided intermediate *in vivo* efficacy using LVS parenteral challenge, which was reflected by intermediate *in vitro* T cell functions. In contrast, splenocytes and PBLs from LVS-R-vaccinated Fischer 344 rats that were at best modestly protected did not control *in vitro* LVS intramacrophage replication. Similarly, splenocytes and PBLs from HK-LVS-vaccinated rats did not control *in vitro* LVS intramacrophage replication. The observation that *in vitro* control functions of T cells did not reflect completely the *in vivo* efficacy of these vaccines against respiratory challenge of rats suggests that non-T cell activities, such as antibodies or other B cell functions, may play protective roles that are specific to the route of challenge and/or challenge with fully virulent *Ft*. This is consistent with previous studies in which transfer of anti-*Francisella* specific antibodies into naïve rats provided some protection against subsequent *Ft* challenge, possibly by controlling infection while T cell-mediated immunity developed [34].

On the other hand, our data indicate a wide range of humoral immune responses among animals in all vaccine groups (Fig 5 and S1 Table). Notably, antibody levels overlapped between animals in the LVS group, which were 100% protected, and the LVS-R and HK-LVS groups, which were poorly protected. In absence of strong T cell-mediated responses using the latter two vaccines, as suggested by co-culture studies (Fig 2A), B cell functions, including antibody immune responses, may contribute to protection. However, differences between specific antibody levels in sera from LVS-R-vaccinated rats, HK-LVS-vaccinated rats, and naïve rats were minimal. Further, when we compared specific anti-LVS antibody titers of individual animals that survived challenge with those that succumbed, there was no obvious correlation between titers and outcome.

In addition to measuring intramacrophage bacterial growth control, we determined the relative expression of immunologically-related genes in rat PBLs and splenocytes recovered from co-cultures. Because common correlates across animal species may provide the most robust candidates, we focused first on genes identified previously in mouse studies and in PBLs, which are a clinically accessible cell source. We searched for expression patterns that reflected vaccine efficacy in rats *in vivo*. Among those with this pattern, NOS2, IL-21, CCL5, and LTA were consistently and strongly upregulated in LVS-derived PBLs across multiple experiments compared to levels in cells from other vaccine groups, followed by genes such as FasL, IL-2ra, IFN-γ, and CXCL9 (Table 1, Group 1). Of note, IL18bp, Socs1, IRF1, and CCL7 were among those potential correlates that were originally identified using mouse splenocytes, but their relative expression was less consistent in mouse PBLs [12, 13]; nonetheless, these genes were also differentially expressed in rat PBLs. The patterns observed were further highlighted by the analyses of individual splenocytes (S2 Fig). These mediators may therefore represent protective T cell responses against *Francisella* that are in common between mice and rats. Further, screening also included other immunologically-related genes beyond the ones specifically identified as correlate candidates in mouse *Francisella* studies. Here, CCR3, CXCL9, CCL4, and IL-1RA were differentially expressed in rat PBLs and splenocytes (Table 1, Group 2), expanding the working panel further.

Similar to mouse studies [13], we also analyzed the relative expression of selected genes in PBLs and splenocytes within days after vaccination in the absence of *in vitro* re-stimulation (Fig 4). Results indicated that this approach is also worth further exploration. Although animal group size was small, patterns of differential gene regulation between blood from the LVS and LVS-R groups were consistent if not always significant. Despite small group sizes, gene expression of IL-18bp and CXCL11 were clearly differentially regulated within 3 days after vaccination both in rat PBLs (Fig 4A) and mouse PBLs [13]; other genes, such as IFN-γ and CCR5, were differentially regulated 2 weeks after vaccination. Quantitative differences in gene expression were smaller in splenocytes than in PBLs, but the pattern of most markers overlapped between the two cell sources. These results corroborate mouse studies and support developing the use of gene expression in blood PBLs to quickly monitor efficacious immune responses, an approach that is amenable to clinical studies.

The complexity of immune responses against intracellular bacteria coupled with assay complexities inevitably contributes to variabilities between individuals. We evaluated this variability by studying splenocytes from individual animals, and analyzed eleven potential correlate measurements (Figs 6 and 7 and S2 Fig). Although the differences between the LVS group compared to LVS-R, HK-LVS and naïve groups were significant for each of these measurements, overlap between groups was evident (Fig 6). By using the values of only one correlate, either CFU or gene expression data, 59.1–83.1% of LVS-vaccinated rats were correctly classified as distinct from all non-LVS vaccinated animals (S2 Table). However, combining two or more correlates into the analytical models progressively reduced the effect of variability and improved sensitivity and specificity (S3 Table). Many combinations were valuable, but the highest corrected classified values were obtained using 5–7 correlates, including CFU and IFN-γ in almost in all combinations (Fig 7 and Table 2). These models were built by using data derived from splenocytes, but support proceeding to more difficult studies using PBLs. To evaluate whether this multivariate approach improves correct classification when the differences between groups were minimal and no single mediator’s values provided significant differences (Fig 6), we further compared all values from the LVS-R group to the naïve group. Although modest, correct classification improved from ~ 50% to ~ 60% by using models with 5–7 variables, again illustrating the power of multiple parameters to facilitate discrimination between vaccination groups.

Taken together, these studies demonstrate that the use of hierarchical vaccine panels coupled with assessments of *in vitro* control of intramacrophage bacterial growth and relative gene expression in responding lymphocytes provides a powerful means to predict successful vaccination against virulent *Francisella* challenge in multiple animal species. This strategy therefore merits advancement to non-human primates and ultimately humans [29]. Gene expression signatures have been explored to discriminate diagnosis of infectious diseases [35], including tuberculosis [36], as well as to evaluate immune responses against *Francisella* [16, 37] in comparison to healthy subjects. Similarly, our results anticipate that the approach of coupling mechanistically relevant correlates candidates with mathematical models may be used to predict the probability that an individual has been treated with a protective or suboptimal vaccine [38].

## Supporting information

S1 Fig Spleen cell populations differ from PBLs and both cell populations differ slightly from the corresponding cells recovered after co-culture. Single cell preparations obtained from spleens and PBLs of naïve and vaccinated rats were stained with a panel of fluorescent antibodies to cell surface markers and with a fluorescent viability dye. After exclusion of fragments and aggregates by SSC-A vs. FSC-A and FSC-W vs. FSC-H, cells were initially gated for viable leukocytes (live CD45^+^). B and T cells were then identified as CD45R^+^ CD45RA^+^ CD3^-^ (B cells), CD3^+^ CD4^+^CD45R^-^CD45RA^-^ (CD4^+^ T cells), or CD3^+^ CD8a^+^CD45R^-^CD45RA (CD8^+^ T cells). The remaining non-B and non-T cells were analyzed using the CD11b/c marker to identify a combination of dendritic cells, neutrophils, and macrophages. Cells populations that were negative for all markers included NK and NK T cells, for which specific markers were not available for rat lymphocytes. Values shown are the average percent leukocytes identified in the indicated cell preparations from naïve and vaccinated rats used for 8–9 co-culture experiments; error bars indicate standard deviation. Results are shown for cells at the start of co-cultures (Panel A) and the corresponding cells recovered after 2–3 days in co-cultures (Panel B).
(TIF)

S2 Fig Gene expression of selected correlates of protection is differentially up-regulated in splenocytes recovered from co-cultures using spleens from individual rats. BMMΦ from Fischer 344 rats were infected with LVS and co-cultured with splenocytes obtained from naïve Fischer 344 rats or rats vaccinated with LVS, LVS-R, or HK-LVS. Splenocytes from 22 rats for each group were analyzed individually in studies comprised of 6–8 separate experiments. After two days of co-culture, splenocytes were recovered and used to purify total RNA, then BMMΦ were lysed to evaluate the recovery of intracellular bacteria. Semi-quantitative gene expression analyses were performed using the indicated sets of primers/probes, chosen among those that best reflected the hierarchy of *in vivo* efficacy. Data are depicted as heat maps derived from values for CFU/ml of viable bacteria for triplicate samples and by the ΔCt values for each individual gene, determined for each individual animal (horizontal lines).
(TIF)

S1 Table Anti-LVS IgG and IgM titers of vaccinated rats. Sera from individual rats were obtained 2–3 weeks after vaccination and analyzed for anti-LVS total IgG and IgM antibodies. Sera from four sets of vaccinations were tested, for a total of 27–35 sera for each vaccine group. Shown are medians and ranges of antibody titers, indicated as sera dilutions, which were obtained using data from 4–16 animals for each vaccine group. Data for the HK-LVS group from vaccination 1 were excluded because of vaccination anomalies.
(PDF)

S2 Table Sensitivity, specificity and correct classification of 2047 models. Eleven variables, consisting of CFU and gene expression values, were used alone or in all possible combinations to build 2047 experimental models. Sensitivity, Specificity and Correct Classification for all 2047 models were calculated and the results were sorted according to the Correct Classification values.
(PDF)

S3 Table Correct classification for linear discriminant analyses. Experimental models consisting of 11 possible variables were analyzed. Shown here are models that have either one explanatory variable (the same name in column and row) or two explanatory variables (different names in the column and the row), for a total of 66 models. Sensitivities and specificity were calculated by discriminating individuals vaccinated with LVS from naïve, as well as from individuals vaccinated with LVS-R or HK-LVS. The corrected classification values is shown, calculated as average of sensitivity and specificity.
(PDF)
