# Supplementary figures and images for: A panel of correlates predicts vaccine-induced protection of rats against respiratory challenge with virulent Francisella tularensis
Source: PLoS One. 2018 May 25;13(5):e0198140. doi: 10.1371/journal.pone.0198140 (PMC5969757; doi:10.1371/journal.pone.0198140)

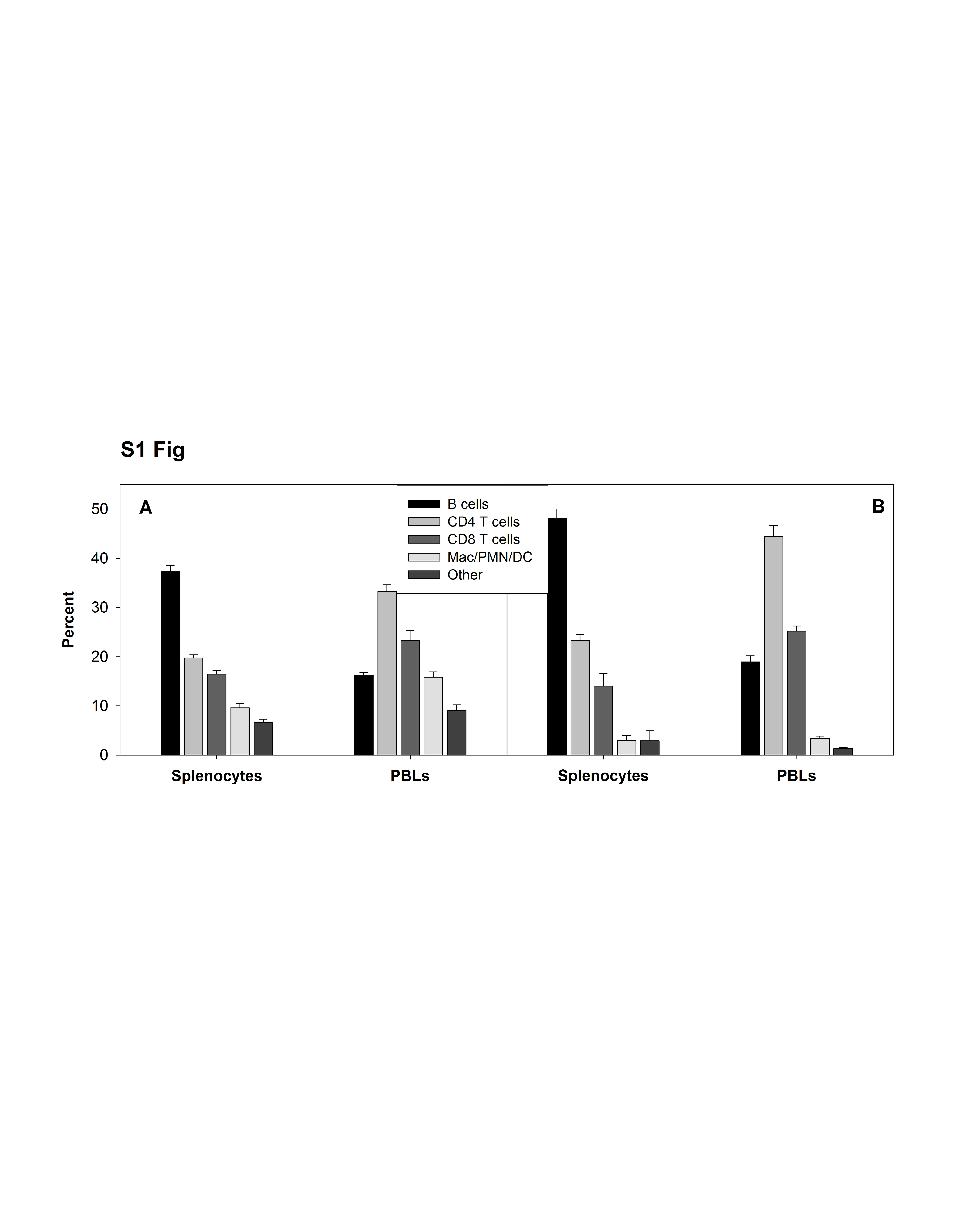

Supplement: S1 Fig — Single cell preparations obtained from spleens and PBLs of naïve and vaccinated rats were stained with a panel of fluorescent antibodies to cell surface markers and with a fluorescent viability dye. After exclusion of fragments and aggregates by SSC-A vs. FSC-A and FSC-W vs. FSC-H, cells were initially gated for viable leukocytes (live CD45+). B and T cells were then identified as CD45R+ CD45RA+ CD3- (B cells), CD3+ CD4+CD45R-CD45RA- (CD4+ T cells), or CD3+ CD8a+CD45R-CD45RA (CD8+ T cells). The remaining non-B and non-T cells were analyzed using the CD11b/c marker to identify a combination of dendritic cells, neutrophils, and macrophages. Cells populations that were negative for all markers included NK and NK T cells, for which specific markers were not available for rat lymphocytes. Values shown are the average percent leukocytes identified in the indicated cell preparations from naïve and vaccinated rats used for 8–9 co-culture experiments; error bars indicate standard deviation. Results are shown for cells at the start of co-cultures (Panel A) and the corresponding cells recovered after 2–3 days in co-cultures (Panel B). (TIF) [file pone.0198140.s001.tif]

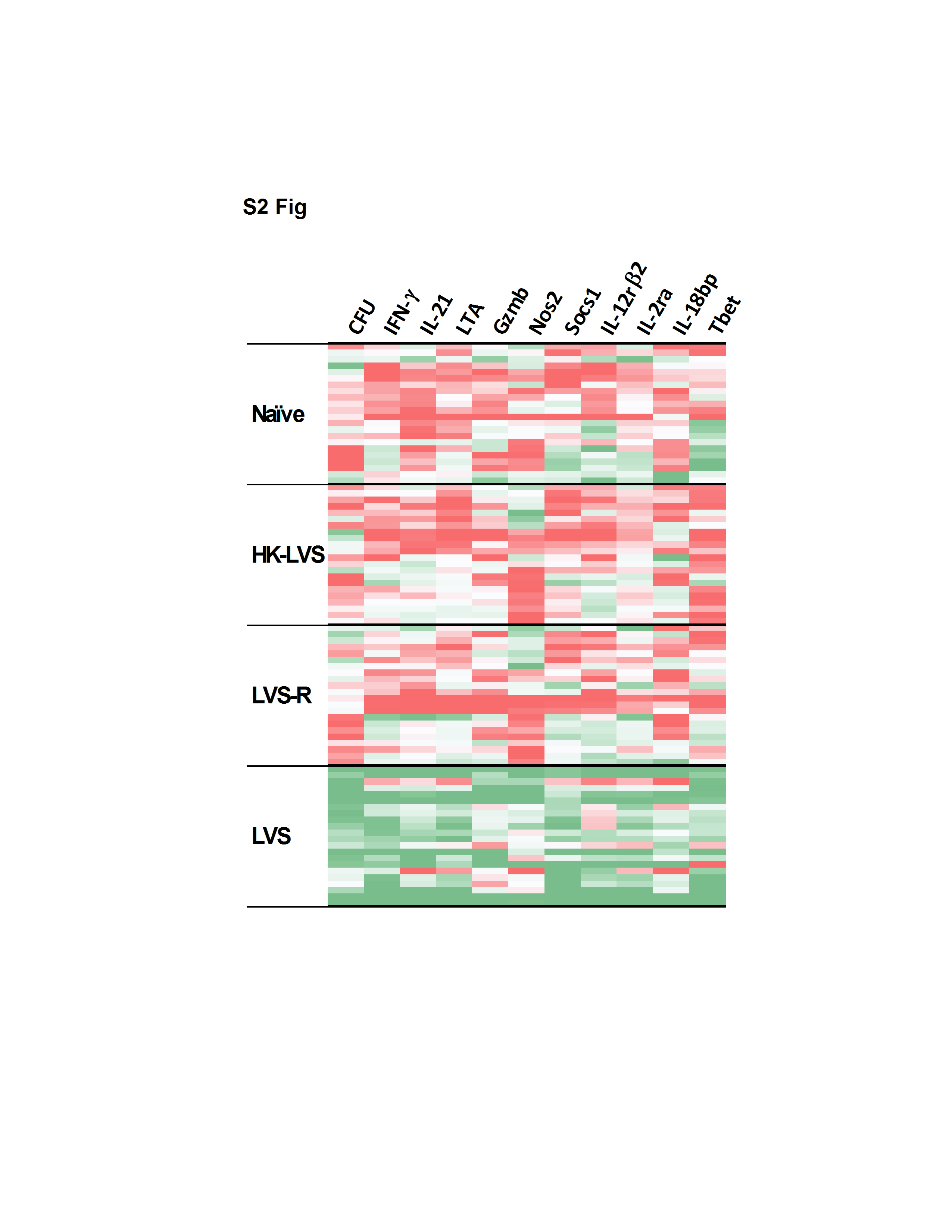

Supplement: S2 Fig — BMMΦ from Fischer 344 rats were infected with LVS and co-cultured with splenocytes obtained from naïve Fischer 344 rats or rats vaccinated with LVS, LVS-R, or HK-LVS. Splenocytes from 22 rats for each group were analyzed individually in studies comprised of 6–8 separate experiments. After two days of co-culture, splenocytes were recovered and used to purify total RNA, then BMMΦ were lysed to evaluate the recovery of intracellular bacteria. Semi-quantitative gene expression analyses were performed using the indicated sets of primers/probes, chosen among those that best reflected the hierarchy of in vivo efficacy. Data are depicted as heat maps derived from values for CFU/ml of viable bacteria for triplicate samples and by the ΔCt values for each individual gene, determined for each individual animal (horizontal lines). (TIF) [file pone.0198140.s002.TIF]
