# Supplementary material for: A panel of correlates predicts vaccine-induced protection of rats against respiratory challenge with virulent Francisella tularensis
Source: PLoS One. 2018 May 25;13(5):e0198140. doi: 10.1371/journal.pone.0198140 (PMC5969757; doi:10.1371/journal.pone.0198140)
Supplement: S1 Table — Sera from individual rats were obtained 2–3 weeks after vaccination and analyzed for anti-LVS total IgG and IgM antibodies. Sera from four sets of vaccinations were tested, for a total of 27–35 sera for each vaccine group. Shown are medians and ranges of antibody titers, indicated as sera dilutions, which were obtained using data from 4–16 animals for each vaccine group. Data for the HK-LVS group from vaccination 1 were excluded because of vaccination anomalies. (PDF) [file pone.0198140.s003.pdf]

S1 Table. Anti-LVS IgG and IgM titers of vaccinated rats

IgG

**Vaccination**	**Naive**	**LVS**	**LVS-R**	**HK-LVS**
**1**	400<br>< 400 - 800	6400<br>1600 - 6400	400<br>< 400 - 800	
**2**	400<br>< 400 - 800	6400<br>3200 - 6400	800<br>< 400 - 1600	200<br>< 400 - 1600
**3**	< 400	12800<br>3200 - >12800	800<br>< 400 - 1600	400<br>< 400 - > 3200
**4**	200	4800<br>3200 - 6400	400<br>< 200 - >1600	400<br>< 200 - 800

IgM

**Vaccination**	**Naive**	**LVS**	**LVS-R**	**HK-LVS**
**1**	< 400	3200<br>1600 - 12800	<400	
**2**	200<br>< 200 - 400	6400<br>1600 - 12800	400<br>< 200 - 800	400<br>400 - 1600
**3**	200<br>< 400	51200<br>25600 - > 25600	800<br>< 400 - 1600	600<br>< 400 - 1600
**4**	200<br><200 - 400	25600<br>➢ 12800	200<br>< 200 - 400	1200<br>200 - > 1600

Sera from individual rats were obtained 2-3 weeks after vaccination and analyzed for anti-LVS total IgG and IgM antibodies. Sera from four sets of vaccinations were tested, for a total of 27-35 sera for each vaccine group. Shown are medians and ranges of antibody titers, indicated as sera dilutions, which were obtained using data from 4-16 animals for each vaccine group. Data of the HK-LVS group from vaccination 1 were excluded because of vaccination anomalies.
