# Supplementary material for: A panel of correlates predicts vaccine-induced protection of rats against respiratory challenge with virulent Francisella tularensis
Source: PLoS One. 2018 May 25;13(5):e0198140. doi: 10.1371/journal.pone.0198140 (PMC5969757; doi:10.1371/journal.pone.0198140)
Supplement: S2 Table — Eleven variables, consisting of CFU and gene expression values, were used alone or in all possible combinations to build 2047 experimental models. Sensitivity, Specificity and Correct Classification for all 2047 models were calculated and the results were sorted according to the Correct Classification values. (PDF) [file pone.0198140.s004.pdf]

S2 Table. Sensitivity, Specificity and Correct Classification of 2047 models

Variables in the model	Sensitivity	Specificity	Correctly Classified	Number of variables in the model
CFU IFNg LTA Nos2 Socs1 IL-2ra	0.96	0.989	0.974	6
CFU IFNg LTA Nos2 Socs1	0.957	0.989	0.973	5
CFU IFNg LTA Socs1 IL-2ra	0.955	0.991	0.973	5
CFU IFNg LTA Nos2 Socs1 IL-12rb2	0.956	0.99	0.973	6
CFU IFNg LTA Gzmb Socs1 IL-2ra	0.963	0.983	0.973	6
CFU IFNg LTA Socs1 IL-12rb2 IL-2ra	0.955	0.991	0.973	6
CFU IFNg LTA Nos2 Socs1 IL-12rb2 IL-18bp	0.957	0.988	0.973	7
CFU IFNg LTA Gzmb Nos2 Socs1	0.961	0.982	0.972	6
CFU IFNg LTA Nos2 Socs1 IL-18bp	0.956	0.988	0.972	6
CFU IFNg LTA Gzmb Socs1 IL-12rb2 IL-2ra	0.96	0.983	0.972	7
CFU IFNg LTA Gzmb Socs1	0.96	0.983	0.971	5
CFU IFNg LTA Gzmb Socs1 IL-12rb2	0.957	0.984	0.971	6
CFU IFNg LTA Gzmb Socs1 IL-18bp	0.958	0.984	0.971	6
CFU IFNg LTA Gzmb Nos2 Socs1 IL-12rb2	0.96	0.983	0.971	7
CFU IFNg LTA Nos2 Socs1 IL-12rb2 IL-2ra	0.952	0.989	0.971	7
CFU IFNg LTA Gzmb Nos2 Socs1 IL-2ra	0.958	0.982	0.97	7
CFU IFNg LTA Gzmb Nos2 Socs1 IL-18bp	0.957	0.982	0.969	7
CFU IFNg LTA Gzmb Socs1 IL-12rb2 IL-18bp	0.955	0.984	0.969	7
CFU IFNg LTA Gzmb Socs1 Tbet	0.952	0.984	0.968	6
CFU IFNg IL-21 LTA Gzmb Socs1 IL-2ra	0.951	0.984	0.968	7
CFU IFNg LTA Gzmb Nos2 Socs1 IL-12rb2 IL-2ra	0.953	0.982	0.968	8
CFU IFNg LTA Gzmb Nos2 Socs1 IL-12rb2 IL-18bp	0.955	0.982	0.968	8
CFU IFNg LTA Socs1	0.941	0.993	0.967	4
CFU IFNg IL-21 LTA Socs1	0.944	0.991	0.967	5
CFU IFNg LTA Socs1 IL-12rb2	0.941	0.993	0.967	5
CFU IFNg IL-21 LTA Gzmb Socs1	0.95	0.985	0.967	6
CFU IFNg IL-21 LTA Socs1 IL-2ra	0.944	0.99	0.967	6
CFU IFNg LTA Socs1 IL-2ra IL-18bp	0.943	0.991	0.967	6
CFU IFNg LTA Nos2 Socs1 Tbet	0.944	0.99	0.967	6
CFU IFNg LTA Socs1 IL-18bp	0.94	0.992	0.966	5
CFU IFNg IL-21 LTA Gzmb Socs1 Tbet	0.949	0.983	0.966	7
CFU IFNg LTA Nos2 Socs1 IL-18bp Tbet	0.944	0.987	0.966	7
CFU IFNg LTA Socs1 IL-12rb2 IL-18bp	0.938	0.993	0.965	6
CFU IFNg LTA Nos2 Socs1 IL-2ra IL-18bp	0.942	0.987	0.965	7
CFU IFNg LTA Socs1 IL-12rb2 IL-2ra IL-18bp	0.94	0.991	0.965	7
CFU IFNg LTA Socs1 Tbet	0.937	0.992	0.964	5
CFU IFNg IL-21 LTA Socs1 IL-12rb2	0.937	0.991	0.964	6
CFU IFNg LTA Socs1 IL-2ra Tbet	0.938	0.99	0.964	6
CFU IFNg IL-21 LTA Socs1 Tbet	0.934	0.991	0.963	6
CFU IFNg LTA Socs1 IL-18bp Tbet	0.935	0.991	0.963	6
CFU IFNg IL-21 LTA Gzmb Socs1 IL-12rb2	0.94	0.985	0.963	7
CFU IFNg LTA Nos2 Socs1 IL-12rb2 IL-2ra IL-18bp	0.939	0.987	0.963	8
CFU IFNg LTA Gzmb Socs1 IL-2ra IL-18bp	0.941	0.983	0.962	7
CFU IFNg LTA Gzmb Nos2 Socs1 Tbet	0.941	0.983	0.962	7
CFU IFNg LTA Gzmb Socs1 IL-2ra Tbet	0.94	0.983	0.962	7
CFU IFNg IL-21 LTA Nos2 Socs1	0.931	0.988	0.96	6
CFU IFNg LTA Gzmb Socs1 IL-12rb2 Tbet	0.936	0.984	0.96	7
CFU IFNg LTA Gzmb Socs1 IL-12rb2 IL-2ra IL-18bp	0.938	0.983	0.96	8
CFU IFNg IL-21 LTA Gzmb Socs1 IL-2ra Tbet	0.938	0.982	0.96	8
CFU IFNg LTA Gzmb Socs1 IL-18bp Tbet	0.935	0.983	0.959	7
CFU IFNg LTA Socs1 IL-12rb2 Tbet	0.924	0.992	0.958	6
CFU IFNg IL-21 LTA Gzmb Nos2 Socs1	0.929	0.983	0.956	7

CFU IFNg LTA Gzmb Nos2 Socs1 IL-2ra IL-18bp	0.931	0.982	0.956	8
CFU IFNg IL-21 LTA Socs1 IL-12rb2 IL-2ra	0.92	0.99	0.955	7
CFU IFNg LTA Gzmb Nos2 Socs1 IL-12rb2 IL-2ra IL-18bp	0.928	0.982	0.955	9
CFU IFNg IL-21 LTA Nos2 Socs1 IL-2ra	0.921	0.988	0.954	7
CFU IFNg LTA Gzmb Nos2 Socs1 IL-18bp Tbet	0.924	0.982	0.953	8
CFU IFNg LTA Socs1 IL-12rb2 IL-18bp Tbet	0.912	0.992	0.952	7
CFU IFNg IL-21 LTA Nos2 Socs1 IL-12rb2	0.914	0.989	0.951	7
CFU IFNg LTA Nos2 Socs1 IL-2ra Tbet	0.914	0.989	0.951	7
CFU IFNg IL-21 LTA Gzmb Socs1 IL-12rb2 IL-2ra	0.918	0.984	0.951	8
CFU IFNg IL-21 LTA Socs1 IL-2ra Tbet	0.911	0.99	0.95	7
CFU IFNg IL-21 LTA Nos2 Socs1 IL-12rb2 IL-2ra	0.911	0.988	0.95	8
CFU IFNg IL-21 LTA Gzmb Nos2 Socs1 IL-2ra	0.914	0.983	0.949	8
CFU IFNg IL-21 LTA Nos2 Socs1 IL-12rb2 IL-18bp	0.911	0.987	0.949	8
CFU IFNg LTA Gzmb Socs1 IL-12rb2 IL-18bp Tbet	0.913	0.985	0.949	8
CFU IFNg LTA Nos2 Socs1 IL-12rb2 IL-18bp Tbet	0.91	0.988	0.949	8
CFU IFNg Socs1 IL-2ra	0.911	0.985	0.948	4
CFU IFNg IL-21 LTA Nos2 Socs1 IL-18bp	0.91	0.987	0.948	7
CFU IFNg IL-21 LTA Nos2 Socs1 IL-2ra IL-18bp	0.91	0.986	0.948	8
CFU IFNg IL-21 LTA Gzmb Nos2 Socs1 Tbet	0.914	0.982	0.948	8
CFU IFNg LTA Nos2 Socs1 IL-2ra IL-18bp Tbet	0.909	0.986	0.948	8
CFU IFNg IL-21 LTA Nos2 Socs1 IL-12rb2 IL-2ra IL-18bp	0.91	0.987	0.948	9
CFU IFNg IL-21 LTA Nos2 Socs1 Tbet	0.905	0.989	0.947	7
CFU IFNg LTA Socs1 IL-2ra IL-18bp Tbet	0.905	0.989	0.947	7
CFU IFNg LTA Gzmb Nos2 Socs1 IL-2ra Tbet	0.91	0.983	0.946	8
CFU IFNg IL-21 LTA Socs1 IL-2ra IL-18bp	0.9	0.99	0.945	7
CFU IFNg IL-21 LTA Gzmb Nos2 Socs1 IL-12rb2	0.905	0.984	0.945	8
CFU IFNg IL-21 LTA Gzmb Socs1 IL-2ra IL-18bp	0.905	0.984	0.945	8
CFU IFNg IL-21 LTA Socs1 IL-12rb2 IL-2ra IL-18bp	0.899	0.99	0.945	8
CFU IFNg IL-21 LTA Nos2 Socs1 IL-2ra Tbet	0.903	0.988	0.945	8
CFU IFNg IL-21 LTA Nos2 Socs1 IL-18bp Tbet	0.903	0.987	0.945	8
CFU IFNg LTA Gzmb Socs1 IL-2ra IL-18bp Tbet	0.907	0.983	0.945	8
CFU IFNg IL-21 LTA Socs1 IL-18bp	0.897	0.991	0.944	6
CFU IFNg IL-21 LTA Gzmb Socs1 IL-18bp	0.904	0.985	0.944	7
CFU IFNg IL-21 LTA Socs1 IL-12rb2 IL-18bp	0.897	0.991	0.944	7
CFU IFNg LTA Nos2 Socs1 IL-12rb2 Tbet	0.898	0.991	0.944	7
CFU IFNg IL-21 LTA Gzmb Socs1 IL-18bp Tbet	0.906	0.982	0.944	8
CFU IFNg LTA Gzmb Nos2 Socs1 IL-12rb2 IL-18bp Tbet	0.905	0.984	0.944	9
CFU IFNg IL-21 LTA Gzmb Socs1 IL-2ra IL-18bp Tbet	0.906	0.982	0.944	9
CFU IFNg IL-21 LTA Nos2 Socs1 IL-2ra IL-18bp Tbet	0.902	0.986	0.944	9
CFU IFNg IL-21 LTA Gzmb Nos2 Socs1 IL-18bp	0.904	0.983	0.943	8
CFU IFNg IL-21 LTA Gzmb Socs1 IL-12rb2 Tbet	0.903	0.983	0.943	8
CFU IFNg IL-21 LTA Nos2 Socs1 IL-12rb2 Tbet	0.897	0.99	0.943	8
CFU IFNg LTA Gzmb Nos2 Socs1 IL-12rb2 Tbet	0.902	0.984	0.943	8
CFU IFNg LTA Nos2 Socs1 IL-12rb2 IL-2ra Tbet	0.897	0.99	0.943	8
CFU IFNg IL-21 LTA Gzmb Nos2 Socs1 IL-12rb2 IL-2ra	0.902	0.984	0.943	9
CFU IFNg IL-21 LTA Gzmb Nos2 Socs1 IL-12rb2 IL-18bp	0.902	0.984	0.943	9
CFU IFNg IL-21 LTA Gzmb Socs1 IL-12rb2 IL-2ra IL-18bp	0.901	0.984	0.943	9
CFU IFNg IL-21 LTA Gzmb Nos2 Socs1 IL-18bp Tbet	0.904	0.981	0.943	9
CFU IFNg LTA Gzmb Nos2 Socs1 IL-2ra IL-18bp Tbet	0.903	0.982	0.943	9
CFU Socs1 Tbet	0.91	0.975	0.942	3
CFU IFNg LTA Socs1 IL-12rb2 IL-2ra Tbet	0.893	0.991	0.942	7
CFU IFNg IL-21 LTA Gzmb Socs1 IL-12rb2 IL-18bp	0.9	0.985	0.942	8
CFU IFNg LTA Gzmb Socs1 IL-12rb2 IL-2ra Tbet	0.9	0.984	0.942	8
CFU IFNg IL-21 LTA Gzmb Nos2 Socs1 IL-2ra IL-18bp	0.902	0.982	0.942	9
CFU IFNg IL-21 LTA Gzmb Nos2 Socs1 IL-2ra Tbet	0.902	0.982	0.942	9
CFU IFNg IL-21 LTA Nos2 Socs1 IL-12rb2 IL-2ra Tbet	0.895	0.989	0.942	9
CFU IFNg IL-21 LTA Socs1 IL-12rb2 Tbet	0.89	0.991	0.941	7

CFU IFNg IL-21 LTA Socs1 IL-18bp Tbet	0.891	0.99	0.941	7
CFU IFNg LTA Nos2 Socs1 IL-12rb2 IL-2ra IL-18bp Tbet	0.896	0.987	0.941	9
CFU IFNg IL-21 LTA Gzmb Nos2 Socs1 IL-12rb2 IL-2ra IL-18bp	0.9	0.983	0.941	10
CFU IFNg IL-21 LTA Gzmb Nos2 Socs1 IL-2ra IL-18bp Tbet	0.902	0.98	0.941	10
CFU Gzmb Socs1 Tbet	0.91	0.97	0.94	4
CFU Socs1 IL-12rb2 Tbet	0.905	0.974	0.94	4
CFU IFNg IL-21 LTA Socs1 IL-12rb2 IL-2ra Tbet	0.89	0.99	0.94	8
CFU IFNg IL-21 LTA Socs1 IL-2ra IL-18bp Tbet	0.891	0.989	0.94	8
CFU IFNg IL-21 LTA Nos2 Socs1 IL-12rb2 IL-18bp Tbet	0.893	0.988	0.94	9
CFU LTA Socs1 Tbet	0.904	0.973	0.939	4
CFU IFNg Gzmb Socs1 IL-2ra	0.9	0.979	0.939	5
CFU Gzmb Socs1 IL-12rb2 Tbet	0.908	0.969	0.939	5
CFU IFNg IL-21 LTA Gzmb Socs1 IL-12rb2 IL-2ra Tbet	0.896	0.983	0.939	9
CFU IFNg Socs1 Tbet	0.889	0.988	0.938	4
CFU IFNg IL-21 LTA Nos2 Socs1 IL-12rb2 IL-2ra IL-18bp Tbet	0.89	0.987	0.938	10
CFU IFNg Socs1 IL-12rb2	0.886	0.989	0.937	4
CFU IL-21 Socs1 Tbet	0.901	0.973	0.937	4
CFU Socs1 IL-2ra Tbet	0.9	0.973	0.937	4
CFU IFNg IL-21 LTA Gzmb Nos2 Socs1 IL-12rb2 Tbet	0.89	0.983	0.937	9
CFU IFNg LTA Gzmb Nos2 Socs1 IL-12rb2 IL-2ra Tbet	0.89	0.984	0.937	9
CFU IFNg LTA Gzmb Nos2 Socs1 IL-12rb2 IL-2ra IL-18bp Tbet	0.891	0.983	0.937	10
CFU Nos2 Socs1 Tbet	0.897	0.975	0.936	4
CFU Socs1 IL-18bp Tbet	0.898	0.974	0.936	4
CFU Gzmb Socs1 IL-18bp Tbet	0.905	0.968	0.936	5
CFU Socs1 IL-2ra IL-18bp Tbet	0.904	0.968	0.936	5
CFU IFNg LTA Socs1 IL-12rb2 IL-2ra IL-18bp Tbet	0.882	0.99	0.936	8
CFU IFNg LTA Gzmb Socs1 IL-12rb2 IL-2ra IL-18bp Tbet	0.888	0.985	0.936	9
CFU IFNg Socs1 IL-12rb2 IL-2ra	0.883	0.987	0.935	5
CFU IFNg Socs1 IL-2ra Tbet	0.884	0.986	0.935	5
CFU IL-21 Socs1 IL-2ra Tbet	0.896	0.973	0.935	5
CFU IFNg IL-21 LTA Gzmb Tbet	0.895	0.975	0.935	6
CFU IFNg IL-21 LTA Gzmb Nos2 Socs1 IL-12rb2 IL-18bp Tbet	0.888	0.983	0.935	10
CFU IFNg IL-21 Socs1	0.882	0.985	0.934	4
CFU IFNg LTA Tbet	0.885	0.983	0.934	4
CFU IFNg IL-21 Socs1 IL-2ra	0.884	0.985	0.934	5
CFU IFNg Nos2 Socs1 IL-2ra	0.883	0.985	0.934	5
CFU IFNg Socs1 IL-2ra IL-18bp	0.882	0.986	0.934	5
CFU Nos2 Socs1 IL-12rb2 Tbet	0.892	0.976	0.934	5
CFU LTA Socs1 IL-18bp Tbet	0.902	0.966	0.934	5
CFU Nos2 Socs1 IL-18bp Tbet	0.896	0.972	0.934	5
CFU IFNg IL-21 LTA Gzmb Nos2 Socs1 IL-12rb2 IL-2ra Tbet	0.887	0.982	0.934	10
CFU IFNg IL-21 LTA Gzmb Nos2 Socs1 IL-12rb2 IL-2ra IL-18bp Tbe	0.885	0.982	0.934	11
CFU IL-21 Gzmb Socs1 Tbet	0.894	0.972	0.933	5
CFU IL-21 Socs1 IL-12rb2 Tbet	0.892	0.973	0.933	5
CFU Gzmb Socs1 IL-2ra Tbet	0.896	0.97	0.933	5
CFU Nos2 Socs1 IL-2ra Tbet	0.891	0.974	0.933	5
CFU IFNg IL-21 Socs1 IL-2ra IL-18bp	0.88	0.985	0.933	6
CFU IFNg IL-21 Socs1 IL-2ra Tbet	0.881	0.985	0.933	6
CFU Gzmb Socs1 IL-2ra IL-18bp Tbet	0.9	0.966	0.933	6
CFU Nos2 Socs1 IL-2ra IL-18bp Tbet	0.898	0.968	0.933	6
CFU IL-21 LTA Gzmb Socs1 IL-18bp Tbet	0.9	0.967	0.933	7
CFU IFNg IL-21 LTA Gzmb Socs1 IL-12rb2 IL-2ra IL-18bp Tbet	0.883	0.983	0.933	10
CFU LTA Nos2 Socs1 Tbet	0.89	0.974	0.932	5
CFU Socs1 IL-12rb2 IL-2ra Tbet	0.891	0.973	0.932	5
CFU Socs1 IL-12rb2 IL-18bp Tbet	0.892	0.972	0.932	5
CFU IL-21 LTA Gzmb Socs1 Tbet	0.894	0.971	0.932	6
CFU LTA Nos2 Socs1 IL-18bp Tbet	0.903	0.962	0.932	6

CFU IFNg Socs1 IL-2ra IL-18bp Tbet	0.878	0.986	0.932	6
CFU IFNg IL-21 Socs1 IL-2ra IL-18bp Tbet	0.878	0.985	0.932	7
CFU IFNg IL-21 LTA Gzmb Socs1 IL-12rb2 IL-18bp Tbet	0.881	0.984	0.932	9
CFU LTA Gzmb Socs1 Tbet	0.892	0.97	0.931	5
CFU IL-21 Nos2 Socs1 Tbet	0.888	0.973	0.931	5
CFU IL-21 Socs1 IL-18bp Tbet	0.896	0.967	0.931	5
CFU IFNg Nos2 Socs1 IL-2ra Tbet	0.875	0.986	0.931	6
CFU Gzmb Socs1 IL-12rb2 IL-18bp Tbet	0.893	0.969	0.931	6
CFU IL-21 Socs1 IL-2ra IL-18bp Tbet	0.894	0.968	0.931	6
CFU IFNg IL-21 LTA Socs1 IL-12rb2 IL-18bp Tbet	0.872	0.991	0.931	8
CFU IFNg IL-21 LTA Socs1 IL-12rb2 IL-2ra IL-18bp Tbet	0.873	0.99	0.931	9
CFU IFNg Nos2 Socs1 IL-2ra IL-18bp	0.874	0.986	0.93	6
CFU IL-21 Gzmb Socs1 IL-12rb2 Tbet	0.888	0.972	0.93	6
CFU IL-21 Gzmb Socs1 IL-2ra Tbet	0.889	0.972	0.93	6
CFU IL-21 Nos2 Socs1 IL-2ra Tbet	0.885	0.975	0.93	6
CFU Gzmb Socs1 IL-12rb2 IL-2ra Tbet	0.892	0.969	0.93	6
CFU LTA Gzmb Socs1 IL-18bp Tbet	0.896	0.963	0.93	6
CFU Nos2 Socs1 IL-12rb2 IL-18bp Tbet	0.891	0.97	0.93	6
CFU IFNg LTA Gzmb	0.894	0.964	0.929	4
CFU IFNg Gzmb Socs1 IL-12rb2	0.879	0.98	0.929	5
CFU IFNg IL-21 Socs1 IL-18bp	0.872	0.986	0.929	5
CFU IFNg IL-21 Socs1 Tbet	0.872	0.986	0.929	5
CFU IFNg IL-21 Gzmb Socs1 IL-2ra	0.875	0.982	0.929	6
CFU IL-21 Nos2 Socs1 IL-18bp Tbet	0.892	0.966	0.929	6
CFU IL-21 Gzmb Socs1 IL-2ra IL-18bp Tbet	0.889	0.969	0.929	7
CFU IFNg Nos2 Socs1 IL-2ra IL-18bp Tbet	0.873	0.985	0.929	7
CFU IL-21 LTA Gzmb Socs1 IL-2ra IL-18bp Tbet	0.891	0.968	0.929	8
CFU IFNg Nos2 Socs1	0.87	0.985	0.928	4
CFU IFNg LTA Gzmb IL-12rb2	0.892	0.964	0.928	5
CFU IFNg Nos2 Socs1 IL-12rb2	0.869	0.987	0.928	5
CFU IL-21 LTA Socs1 Tbet	0.883	0.973	0.928	5
CFU Gzmb Nos2 Socs1 Tbet	0.885	0.972	0.928	5
CFU IFNg IL-21 Nos2 Socs1 IL-2ra	0.872	0.984	0.928	6
CFU IFNg IL-21 Socs1 IL-12rb2 IL-2ra	0.869	0.986	0.928	6
CFU IFNg IL-21 Socs1 IL-18bp Tbet	0.869	0.986	0.928	6
CFU IFNg IL-21 Nos2 Socs1 IL-2ra IL-18bp	0.871	0.985	0.928	7
CFU IFNg IL-21 Nos2 Socs1 IL-2ra Tbet	0.871	0.985	0.928	7
CFU IFNg Tbet	0.871	0.983	0.927	3
CFU IFNg IL-21 Gzmb Socs1	0.873	0.982	0.927	5
CFU IFNg Gzmb Socs1 IL-12rb2 IL-2ra	0.873	0.981	0.927	6
CFU IFNg Socs1 IL-12rb2 IL-2ra IL-18bp	0.868	0.986	0.927	6
CFU Gzmb Nos2 Socs1 IL-12rb2 Tbet	0.882	0.971	0.927	6
CFU IL-21 Socs1 IL-12rb2 IL-2ra Tbet	0.881	0.974	0.927	6
CFU Nos2 Socs1 IL-12rb2 IL-2ra Tbet	0.88	0.974	0.927	6
CFU IL-21 Gzmb Socs1 IL-18bp Tbet	0.885	0.969	0.927	6
CFU Gzmb Nos2 Socs1 IL-18bp Tbet	0.885	0.969	0.927	6
CFU IFNg IL-21 Gzmb Socs1 IL-2ra IL-18bp	0.871	0.982	0.927	7
CFU IL-21 Nos2 Socs1 IL-2ra IL-18bp Tbet	0.887	0.968	0.927	7
CFU IFNg IL-21 Nos2 Socs1 IL-2ra IL-18bp Tbet	0.87	0.985	0.927	8
CFU IFNg LTA Gzmb Tbet	0.876	0.976	0.926	5
CFU IFNg Nos2 Socs1 IL-12rb2 IL-2ra	0.865	0.987	0.926	6
CFU IFNg Gzmb Socs1 IL-2ra IL-18bp	0.872	0.979	0.926	6
CFU IL-21 Nos2 Socs1 IL-12rb2 Tbet	0.877	0.975	0.926	6
CFU Socs1 IL-12rb2 IL-2ra IL-18bp Tbet	0.883	0.969	0.926	6
CFU IL-21 Gzmb Socs1 IL-12rb2 IL-2ra Tbet	0.88	0.972	0.926	7
CFU IFNg Socs1	0.864	0.987	0.925	3
CFU IFNg Gzmb Socs1	0.873	0.976	0.925	4

CFU IFNg LTA Gzmb IL-2ra	0.892	0.959	0.925	5
CFU IFNg Gzmb Socs1 Tbet	0.87	0.98	0.925	5
CFU IFNg IL-21 Nos2 Socs1 IL-18bp	0.865	0.986	0.925	6
CFU IFNg IL-21 Nos2 Socs1 Tbet	0.865	0.985	0.925	6
CFU IFNg Gzmb Socs1 IL-2ra Tbet	0.87	0.981	0.925	6
CFU IFNg Socs1 IL-12rb2 IL-2ra Tbet	0.862	0.988	0.925	6
CFU IL-21 LTA Socs1 IL-18bp Tbet	0.885	0.965	0.925	6
CFU IFNg IL-21 LTA Gzmb IL-2ra Tbet	0.876	0.974	0.925	7
CFU IFNg IL-21 Nos2 Socs1 IL-18bp Tbet	0.865	0.985	0.925	7
CFU IFNg IL-21 Nos2 Socs1	0.864	0.984	0.924	5
CFU IFNg IL-21 Socs1 IL-12rb2	0.861	0.988	0.924	5
CFU IFNg Socs1 IL-12rb2 Tbet	0.859	0.99	0.924	5
CFU IFNg Gzmb Nos2 Socs1 IL-2ra	0.869	0.979	0.924	6
CFU IFNg LTA Gzmb IL-12rb2 IL-2ra	0.89	0.958	0.924	6
CFU IFNg IL-21 Gzmb Socs1 IL-12rb2 IL-2ra	0.861	0.986	0.924	7
CFU IFNg IL-21 Socs1 IL-12rb2 IL-2ra IL-18bp	0.863	0.986	0.924	7
CFU IFNg IL-21 Gzmb Socs1 IL-2ra Tbet	0.867	0.981	0.924	7
CFU Gzmb Nos2 Socs1 IL-12rb2 IL-18bp Tbet	0.88	0.968	0.924	7
CFU Gzmb Nos2 Socs1 IL-2ra IL-18bp Tbet	0.879	0.968	0.924	7
CFU IL-21 Gzmb Tbet	0.879	0.966	0.923	4
CFU IFNg Socs1 IL-12rb2 IL-18bp	0.858	0.989	0.923	5
CFU IFNg Nos2 Socs1 Tbet	0.859	0.986	0.923	5
CFU IL-21 Gzmb IL-18bp Tbet	0.881	0.965	0.923	5
CFU Gzmb Nos2 Socs1 IL-2ra Tbet	0.874	0.972	0.923	6
CFU IL-21 Gzmb IL-12rb2 IL-18bp Tbet	0.88	0.967	0.923	6
CFU IFNg Nos2 Socs1 IL-12rb2 IL-2ra IL-18bp	0.86	0.986	0.923	7
CFU IFNg IL-21 Socs1 IL-12rb2 IL-2ra Tbet	0.859	0.988	0.923	7
CFU IFNg Gzmb Socs1 IL-2ra IL-18bp Tbet	0.866	0.98	0.923	7
CFU Gzmb Socs1 IL-12rb2 IL-2ra IL-18bp Tbet	0.879	0.967	0.923	7
CFU Nos2 Socs1 IL-12rb2 IL-2ra IL-18bp Tbet	0.879	0.968	0.923	7
CFU IL-21 LTA Gzmb Nos2 Socs1 IL-18bp Tbet	0.878	0.967	0.923	8
CFU IFNg LTA IL-12rb2	0.869	0.974	0.922	4
CFU IFNg LTA Gzmb Nos2	0.882	0.963	0.922	5
CFU LTA Socs1 IL-2ra Tbet	0.87	0.973	0.922	5
CFU IFNg LTA Gzmb Nos2 IL-2ra	0.887	0.958	0.922	6
CFU IFNg IL-21 Gzmb Socs1 IL-18bp	0.862	0.983	0.922	6
CFU IFNg Nos2 Socs1 IL-12rb2 Tbet	0.854	0.989	0.922	6
CFU LTA Socs1 IL-2ra IL-18bp Tbet	0.878	0.965	0.922	6
CFU IFNg IL-21 Nos2 Socs1 IL-12rb2 IL-2ra	0.858	0.986	0.922	7
CFU IFNg IL-21 LTA Gzmb Nos2 Tbet	0.869	0.974	0.922	7
CFU IFNg IL-21 LTA Gzmb IL-12rb2 Tbet	0.869	0.976	0.922	7
CFU IFNg IL-21 LTA Gzmb IL-18bp Tbet	0.869	0.975	0.922	7
CFU IL-21 LTA Nos2 Socs1 IL-18bp Tbet	0.882	0.962	0.922	7
CFU IFNg IL-21 Gzmb Socs1 IL-2ra IL-18bp Tbet	0.863	0.981	0.922	8
CFU IFNg LTA Nos2	0.874	0.969	0.921	4
CFU IFNg IL-21 LTA Gzmb	0.879	0.963	0.921	5
CFU IL-21 Gzmb IL-12rb2 Tbet	0.876	0.965	0.921	5
CFU IFNg LTA Gzmb Nos2 Tbet	0.867	0.975	0.921	6
CFU IFNg LTA Gzmb IL-12rb2 Tbet	0.865	0.977	0.921	6
CFU IFNg LTA Nos2 IL-12rb2 Tbet	0.86	0.982	0.921	6
CFU IFNg IL-21 Socs1 IL-12rb2 Tbet	0.853	0.989	0.921	6
CFU IFNg LTA Gzmb IL-18bp Tbet	0.866	0.976	0.921	6
CFU IL-21 Socs1 IL-12rb2 IL-18bp Tbet	0.87	0.972	0.921	6
CFU IFNg Nos2 Socs1 IL-12rb2 IL-2ra Tbet	0.855	0.988	0.921	7
CFU IFNg LTA Gzmb Nos2 IL-18bp Tbet	0.867	0.974	0.921	7
CFU IFNg LTA Nos2 IL-12rb2 IL-18bp Tbet	0.862	0.979	0.921	7
CFU IL-21 Gzmb Socs1 IL-12rb2 IL-18bp Tbet	0.871	0.971	0.921	7

CFU IFNg IL-21 Gzmb Socs1 IL-12rb2 IL-2ra IL-18bp	0.855	0.986	0.921	8
CFU IFNg IL-21 LTA Gzmb Nos2 IL-12rb2 Tbet	0.867	0.975	0.921	8
CFU IFNg IL-21 LTA Gzmb Nos2 IL-18bp Tbet	0.869	0.973	0.921	8
CFU IFNg IL-21 LTA Gzmb IL-12rb2 IL-18bp Tbet	0.866	0.976	0.921	8
CFU IFNg IL-21 LTA Gzmb Nos2 IL-12rb2 IL-18bp Tbet	0.868	0.974	0.921	9
CFU Socs1	0.875	0.965	0.92	2
CFU IFNg IL-12rb2 Tbet	0.855	0.985	0.92	4
CFU IFNg LTA Nos2 Tbet	0.861	0.98	0.92	5
CFU IFNg LTA IL-12rb2 Tbet	0.855	0.985	0.92	5
CFU IFNg Nos2 IL-12rb2 Tbet	0.857	0.983	0.92	5
CFU IFNg Socs1 IL-18bp Tbet	0.853	0.988	0.92	5
CFU IFNg IL-21 Gzmb Socs1 IL-12rb2	0.853	0.987	0.92	6
CFU IFNg IL-21 Socs1 IL-12rb2 IL-18bp	0.853	0.988	0.92	6
CFU IFNg IL-21 Nos2 IL-12rb2 Tbet	0.857	0.982	0.92	6
CFU IFNg LTA Gzmb IL-2ra Tbet	0.864	0.976	0.92	6
CFU LTA Socs1 IL-12rb2 IL-18bp Tbet	0.87	0.969	0.92	6
CFU IFNg IL-21 Gzmb Nos2 Socs1 IL-2ra	0.859	0.982	0.92	7
CFU IFNg Gzmb Socs1 IL-12rb2 IL-2ra IL-18bp	0.859	0.981	0.92	7
CFU IFNg IL-21 LTA Nos2 IL-12rb2 Tbet	0.858	0.982	0.92	7
CFU IFNg LTA Gzmb Nos2 IL-12rb2 Tbet	0.864	0.976	0.92	7
CFU IL-21 LTA Gzmb Socs1 IL-2ra Tbet	0.869	0.971	0.92	7
CFU IFNg LTA Nos2 IL-12rb2 IL-2ra Tbet	0.857	0.982	0.92	7
CFU Gzmb Nos2 Socs1 IL-12rb2 IL-2ra Tbet	0.869	0.971	0.92	7
CFU IFNg LTA Gzmb IL-12rb2 IL-18bp Tbet	0.862	0.977	0.92	7
CFU IL-21 Gzmb Nos2 IL-12rb2 IL-18bp Tbet	0.874	0.966	0.92	7
CFU IL-21 Nos2 Socs1 IL-12rb2 IL-18bp Tbet	0.87	0.97	0.92	7
CFU IFNg IL-21 Nos2 Socs1 IL-12rb2 IL-2ra IL-18bp	0.855	0.986	0.92	8
CFU IFNg LTA Gzmb Nos2 IL-12rb2 IL-18bp Tbet	0.865	0.976	0.92	8
CFU IL-21 LTA Gzmb Socs1 IL-12rb2 IL-18bp Tbet	0.871	0.969	0.92	8
CFU IFNg LTA	0.862	0.975	0.919	3
CFU Socs1 IL-2ra	0.876	0.962	0.919	3
CFU IFNg Socs1 IL-18bp	0.85	0.989	0.919	4
CFU IFNg LTA Nos2 IL-2ra	0.87	0.968	0.919	5
CFU IFNg IL-21 IL-12rb2 Tbet	0.854	0.984	0.919	5
CFU IFNg Gzmb IL-12rb2 Tbet	0.859	0.978	0.919	5
CFU IL-21 Gzmb IL-2ra Tbet	0.873	0.965	0.919	5
CFU IFNg IL-21 Nos2 Socs1 IL-12rb2	0.852	0.987	0.919	6
CFU IFNg IL-21 LTA Gzmb IL-2ra	0.879	0.959	0.919	6
CFU IFNg IL-21 LTA Nos2 Tbet	0.858	0.98	0.919	6
CFU IL-21 LTA Socs1 IL-2ra Tbet	0.864	0.973	0.919	6
CFU IFNg LTA Nos2 IL-18bp Tbet	0.86	0.978	0.919	6
CFU IFNg Nos2 Socs1 IL-18bp Tbet	0.851	0.986	0.919	6
CFU IFNg Nos2 IL-12rb2 IL-18bp Tbet	0.857	0.981	0.919	6
CFU IFNg Socs1 IL-12rb2 IL-18bp Tbet	0.848	0.989	0.919	6
CFU IL-21 Gzmb IL-2ra IL-18bp Tbet	0.873	0.964	0.919	6
CFU IFNg LTA Gzmb Nos2 IL-2ra Tbet	0.864	0.975	0.919	7
CFU IFNg LTA Gzmb IL-12rb2 IL-2ra Tbet	0.861	0.977	0.919	7
CFU IFNg Gzmb Socs1 IL-12rb2 IL-2ra Tbet	0.854	0.983	0.919	7
CFU LTA Gzmb Nos2 Socs1 IL-18bp Tbet	0.874	0.964	0.919	7
CFU IFNg IL-21 Gzmb Nos2 Socs1 IL-2ra IL-18bp	0.856	0.982	0.919	8
CFU IFNg IL-21 LTA Gzmb Nos2 IL-2ra Tbet	0.865	0.973	0.919	8
CFU IFNg IL-21 LTA Gzmb IL-12rb2 IL-2ra Tbet	0.864	0.974	0.919	8
CFU IFNg LTA Gzmb Nos2 IL-12rb2 IL-2ra Tbet	0.862	0.976	0.919	8
CFU IFNg IL-21 LTA Nos2 IL-12rb2 IL-18bp Tbet	0.857	0.98	0.919	8
CFU IFNg LTA Gzmb Nos2 IL-2ra IL-18bp Tbet	0.864	0.974	0.919	8
CFU IFNg LTA Nos2 IL-12rb2 IL-2ra IL-18bp Tbet	0.859	0.98	0.919	8
CFU IFNg LTA Gzmb Nos2 IL-12rb2 IL-2ra IL-18bp Tbet	0.863	0.976	0.919	9

CFU IL-21 Socs1	0.876	0.96	0.918	3
CFU Gzmb Socs1	0.877	0.959	0.918	3
CFU Socs1 IL-12rb2	0.873	0.964	0.918	3
CFU Socs1 IL-12rb2 IL-2ra	0.875	0.962	0.918	4
CFU IFNg Gzmb Tbet	0.859	0.976	0.918	4
CFU IL-21 Gzmb Nos2 Tbet	0.87	0.966	0.918	5
CFU LTA Socs1 IL-12rb2 Tbet	0.863	0.974	0.918	5
CFU IFNg IL-12rb2 IL-2ra Tbet	0.852	0.984	0.918	5
CFU IFNg IL-21 Gzmb Socs1 Tbet	0.854	0.981	0.918	6
CFU IL-21 LTA Nos2 Socs1 Tbet	0.863	0.973	0.918	6
CFU IFNg IL-21 LTA IL-12rb2 Tbet	0.851	0.984	0.918	6
CFU IFNg IL-21 Gzmb IL-12rb2 Tbet	0.858	0.978	0.918	6
CFU IFNg Gzmb Socs1 IL-12rb2 Tbet	0.852	0.983	0.918	6
CFU LTA Gzmb Socs1 IL-12rb2 Tbet	0.866	0.969	0.918	6
CFU IFNg LTA Nos2 IL-2ra Tbet	0.856	0.98	0.918	6
CFU IFNg LTA IL-12rb2 IL-2ra Tbet	0.851	0.984	0.918	6
CFU IFNg Nos2 IL-12rb2 IL-2ra Tbet	0.854	0.983	0.918	6
CFU IL-21 Gzmb Nos2 IL-18bp Tbet	0.872	0.965	0.918	6
CFU IL-21 LTA Gzmb Nos2 Socs1 Tbet	0.865	0.972	0.918	7
CFU IL-21 Gzmb Nos2 Socs1 IL-2ra Tbet	0.861	0.974	0.918	7
CFU IL-21 Nos2 Socs1 IL-12rb2 IL-2ra Tbet	0.86	0.975	0.918	7
CFU IFNg IL-21 Nos2 IL-12rb2 IL-18bp Tbet	0.856	0.981	0.918	7
CFU IFNg Nos2 Socs1 IL-12rb2 IL-18bp Tbet	0.849	0.988	0.918	7
CFU IFNg LTA Gzmb IL-2ra IL-18bp Tbet	0.861	0.975	0.918	7
CFU LTA Nos2 Socs1 IL-2ra IL-18bp Tbet	0.873	0.963	0.918	7
CFU IL-21 Gzmb IL-12rb2 IL-2ra IL-18bp Tbet	0.87	0.965	0.918	7
CFU IFNg IL-21 LTA Nos2 IL-12rb2 IL-2ra Tbet	0.854	0.982	0.918	8
CFU IFNg IL-21 Nos2 Socs1 IL-12rb2 IL-2ra Tbet	0.848	0.988	0.918	8
CFU IFNg IL-21 LTA Gzmb IL-2ra IL-18bp Tbet	0.863	0.973	0.918	8
CFU IFNg LTA Gzmb IL-12rb2 IL-2ra IL-18bp Tbet	0.859	0.977	0.918	8
CFU IL-21 Gzmb Socs1 IL-12rb2 IL-2ra IL-18bp Tbet	0.863	0.972	0.918	8
CFU IFNg IL-21 LTA Gzmb Nos2 IL-12rb2 IL-2ra Tbet	0.863	0.973	0.918	9
CFU IFNg IL-21 LTA Gzmb Nos2 IL-2ra IL-18bp Tbet	0.864	0.972	0.918	9
CFU IL-21 LTA Gzmb Nos2 Socs1 IL-2ra IL-18bp Tbet	0.866	0.969	0.918	9
CFU IFNg IL-21 LTA Gzmb Nos2 IL-12rb2 IL-2ra IL-18bp Tbet	0.862	0.974	0.918	10
CFU IL-21 Socs1 IL-12rb2	0.876	0.958	0.917	4
CFU Gzmb Socs1 IL-12rb2	0.876	0.958	0.917	4
CFU LTA Socs1 IL-2ra	0.871	0.963	0.917	4
CFU IFNg Gzmb Nos2 Socs1	0.858	0.976	0.917	5
CFU IFNg LTA IL-12rb2 IL-2ra	0.862	0.973	0.917	5
CFU IFNg IL-21 LTA Tbet	0.851	0.983	0.917	5
CFU IFNg IL-12rb2 IL-18bp Tbet	0.85	0.984	0.917	5
CFU IL-21 Gzmb Nos2 Socs1 Tbet	0.861	0.973	0.917	6
CFU IL-21 Gzmb Nos2 IL-12rb2 Tbet	0.868	0.965	0.917	6
CFU LTA Gzmb Socs1 IL-2ra Tbet	0.863	0.97	0.917	6
CFU LTA Nos2 Socs1 IL-2ra Tbet	0.859	0.975	0.917	6
CFU IFNg IL-21 IL-12rb2 IL-2ra Tbet	0.85	0.984	0.917	6
CFU IFNg Gzmb IL-12rb2 IL-2ra Tbet	0.856	0.978	0.917	6
CFU IL-21 Gzmb IL-12rb2 IL-2ra Tbet	0.871	0.964	0.917	6
CFU IFNg LTA IL-12rb2 IL-18bp Tbet	0.85	0.984	0.917	6
CFU IFNg Gzmb IL-12rb2 IL-18bp Tbet	0.855	0.978	0.917	6
CFU IFNg IL-21 Nos2 Socs1 IL-12rb2 IL-18bp	0.849	0.986	0.917	7
CFU IFNg IL-21 Nos2 Socs1 IL-12rb2 Tbet	0.846	0.988	0.917	7
CFU IFNg IL-21 LTA Nos2 IL-2ra Tbet	0.853	0.98	0.917	7
CFU IFNg IL-21 Nos2 IL-12rb2 IL-2ra Tbet	0.852	0.982	0.917	7
CFU IFNg IL-21 LTA Nos2 IL-18bp Tbet	0.857	0.978	0.917	7
CFU IFNg LTA Nos2 IL-2ra IL-18bp Tbet	0.856	0.978	0.917	7

CFU IFNg Nos2 IL-12rb2 IL-2ra IL-18bp Tbet	0.853	0.981	0.917	7
CFU IFNg Socs1 IL-12rb2 IL-2ra IL-18bp Tbet	0.848	0.987	0.917	7
CFU IFNg IL-21 Gzmb Socs1 IL-12rb2 IL-2ra Tbet	0.85	0.984	0.917	8
CFU IFNg IL-21 LTA Gzmb IL-12rb2 IL-2ra IL-18bp Tbet	0.859	0.975	0.917	9
CFU LTA Socs1	0.869	0.963	0.916	3
CFU Socs1 IL-18bp	0.867	0.964	0.916	3
CFU IL-21 Socs1 IL-2ra	0.873	0.959	0.916	4
CFU Gzmb Socs1 IL-2ra	0.874	0.959	0.916	4
CFU Gzmb Socs1 IL-12rb2 IL-2ra	0.875	0.957	0.916	5
CFU IFNg IL-21 Gzmb Tbet	0.856	0.976	0.916	5
CFU IFNg IL-21 Nos2 Tbet	0.852	0.98	0.916	5
CFU IFNg LTA IL-2ra Tbet	0.849	0.983	0.916	5
CFU IFNg LTA IL-18bp Tbet	0.849	0.983	0.916	5
CFU IFNg LTA Gzmb Nos2 IL-12rb2	0.869	0.962	0.916	6
CFU IFNg Gzmb Nos2 Socs1 IL-12rb2	0.852	0.979	0.916	6
CFU IFNg Nos2 Socs1 IL-12rb2 IL-18bp	0.845	0.987	0.916	6
CFU LTA Gzmb Nos2 Socs1 Tbet	0.86	0.972	0.916	6
CFU IFNg Gzmb Socs1 IL-18bp Tbet	0.851	0.98	0.916	6
CFU IFNg IL-21 IL-12rb2 IL-18bp Tbet	0.849	0.984	0.916	6
CFU IFNg IL-21 Gzmb Socs1 IL-12rb2 IL-18bp	0.846	0.987	0.916	7
CFU IFNg Gzmb Nos2 Socs1 IL-2ra IL-18bp	0.851	0.98	0.916	7
CFU IL-21 LTA Gzmb Socs1 IL-12rb2 Tbet	0.862	0.97	0.916	7
CFU IFNg IL-21 Gzmb IL-12rb2 IL-2ra Tbet	0.855	0.977	0.916	7
CFU IFNg IL-21 Gzmb Socs1 IL-18bp Tbet	0.851	0.981	0.916	7
CFU IL-21 Gzmb Nos2 Socs1 IL-18bp Tbet	0.861	0.97	0.916	7
CFU IFNg IL-21 Gzmb IL-12rb2 IL-18bp Tbet	0.854	0.978	0.916	7
CFU IFNg Gzmb Nos2 IL-12rb2 IL-18bp Tbet	0.854	0.977	0.916	7
CFU IFNg IL-21 Gzmb Nos2 Socs1 IL-12rb2 IL-2ra	0.847	0.986	0.916	8
CFU IL-21 Gzmb Nos2 Socs1 IL-2ra IL-18bp Tbet	0.861	0.972	0.916	8
CFU IFNg Nos2 Socs1 IL-12rb2 IL-2ra IL-18bp Tbet	0.847	0.986	0.916	8
CFU Gzmb Nos2 Socs1 IL-12rb2 IL-2ra IL-18bp Tbet	0.865	0.967	0.916	8
CFU Nos2 Socs1	0.864	0.966	0.915	3
CFU IFNg IL-21 LTA	0.857	0.973	0.915	4
CFU IL-21 LTA Socs1	0.868	0.961	0.915	4
CFU IL-21 Gzmb Socs1	0.873	0.957	0.915	4
CFU IFNg Gzmb IL-12rb2	0.867	0.963	0.915	4
CFU IFNg LTA IL-2ra	0.856	0.974	0.915	4
CFU Socs1 IL-2ra IL-18bp	0.87	0.96	0.915	4
CFU IFNg IL-21 Tbet	0.849	0.981	0.915	4
CFU IL-21 Socs1 IL-12rb2 IL-2ra	0.873	0.958	0.915	5
CFU LTA Socs1 IL-12rb2 IL-2ra	0.869	0.96	0.915	5
CFU IFNg Gzmb IL-18bp Tbet	0.855	0.976	0.915	5
CFU IFNg IL-21 Gzmb Nos2 Socs1	0.848	0.981	0.915	6
CFU IFNg Gzmb Nos2 IL-12rb2 Tbet	0.853	0.978	0.915	6
CFU IFNg IL-21 Nos2 IL-18bp Tbet	0.852	0.979	0.915	6
CFU IFNg IL-12rb2 IL-2ra IL-18bp Tbet	0.846	0.983	0.915	6
CFU IFNg LTA Gzmb Nos2 IL-12rb2 IL-2ra	0.873	0.958	0.915	7
CFU IFNg IL-21 Gzmb Nos2 Socs1 IL-18bp	0.847	0.983	0.915	7
CFU IFNg IL-21 Gzmb Socs1 IL-12rb2 Tbet	0.845	0.985	0.915	7
CFU IL-21 Gzmb Nos2 Socs1 IL-12rb2 Tbet	0.857	0.973	0.915	7
CFU IFNg IL-21 LTA IL-12rb2 IL-2ra Tbet	0.846	0.984	0.915	7
CFU IFNg IL-21 LTA IL-12rb2 IL-18bp Tbet	0.845	0.984	0.915	7
CFU LTA Nos2 Socs1 IL-12rb2 IL-18bp Tbet	0.864	0.966	0.915	7
CFU IFNg LTA IL-12rb2 IL-2ra IL-18bp Tbet	0.847	0.984	0.915	7
CFU IFNg Gzmb IL-12rb2 IL-2ra IL-18bp Tbet	0.852	0.978	0.915	7
CFU IFNg IL-21 Gzmb Nos2 Socs1 IL-12rb2 IL-2ra IL-18bp	0.844	0.985	0.915	9
CFU IFNg IL-21 LTA Nos2 IL-12rb2 IL-2ra IL-18bp Tbet	0.85	0.98	0.915	9

CFU IFNg Gzmb	0.865	0.963	0.914	3
CFU IFNg IL-12rb2	0.855	0.973	0.914	3
CFU LTA Gzmb Socs1	0.869	0.959	0.914	4
CFU LTA Socs1 IL-12rb2	0.866	0.961	0.914	4
CFU Nos2 Socs1 IL-2ra	0.863	0.965	0.914	4
CFU Socs1 IL-12rb2 IL-18bp	0.864	0.963	0.914	4
CFU IFNg Nos2 Tbet	0.847	0.982	0.914	4
CFU IL-21 Gzmb Socs1 IL-12rb2	0.873	0.955	0.914	5
CFU IFNg IL-21 LTA IL-2ra	0.854	0.973	0.914	5
CFU IFNg Gzmb IL-2ra Tbet	0.853	0.976	0.914	5
CFU IFNg IL-21 IL-18bp Tbet	0.847	0.981	0.914	5
CFU IFNg IL-21 Gzmb IL-2ra Tbet	0.852	0.975	0.914	6
CFU IL-21 Gzmb Nos2 IL-2ra Tbet	0.864	0.965	0.914	6
CFU IFNg IL-21 Gzmb IL-18bp Tbet	0.853	0.976	0.914	6
CFU IFNg Gzmb IL-2ra IL-18bp Tbet	0.852	0.976	0.914	6
CFU IFNg IL-21 Gzmb Nos2 Socs1 IL-12rb2	0.841	0.987	0.914	7
CFU IFNg Gzmb Nos2 IL-12rb2 IL-2ra Tbet	0.85	0.978	0.914	7
CFU LTA Gzmb Socs1 IL-12rb2 IL-18bp Tbet	0.861	0.966	0.914	7
CFU LTA Gzmb Socs1 IL-2ra IL-18bp Tbet	0.865	0.963	0.914	7
CFU IL-21 Socs1 IL-12rb2 IL-2ra IL-18bp Tbet	0.855	0.972	0.914	7
CFU IFNg IL-21 Gzmb Nos2 IL-12rb2 IL-18bp Tbet	0.85	0.977	0.914	8
CFU IFNg IL-21 LTA Nos2 IL-2ra IL-18bp Tbet	0.85	0.978	0.914	8
CFU IFNg Gzmb Nos2 IL-12rb2 IL-2ra IL-18bp Tbet	0.85	0.977	0.914	8
CFU IL-21 Gzmb Nos2 IL-12rb2 IL-2ra IL-18bp Tbet	0.862	0.965	0.914	8
CFU IFNg IL-21 Socs1 IL-12rb2 IL-2ra IL-18bp Tbet	0.841	0.987	0.914	8
CFU Nos2 Socs1 IL-12rb2	0.862	0.965	0.913	4
CFU Gzmb Socs1 IL-18bp	0.869	0.958	0.913	4
CFU Nos2 Socs1 IL-18bp	0.861	0.964	0.913	4
CFU IFNg LTA Nos2 IL-12rb2	0.859	0.968	0.913	5
CFU IL-21 LTA Socs1 IL-12rb2	0.868	0.958	0.913	5
CFU LTA Gzmb Socs1 IL-12rb2	0.871	0.955	0.913	5
CFU IL-21 Gzmb Socs1 IL-2ra	0.869	0.956	0.913	5
CFU LTA Gzmb Socs1 IL-2ra	0.866	0.96	0.913	5
CFU IFNg Nos2 Socs1 IL-18bp	0.838	0.987	0.913	5
CFU Socs1 IL-12rb2 IL-2ra IL-18bp	0.867	0.959	0.913	5
CFU IL-21 LTA Gzmb Tbet	0.862	0.965	0.913	5
CFU IFNg Nos2 IL-18bp Tbet	0.845	0.981	0.913	5
CFU LTA Gzmb Socs1 IL-12rb2 IL-2ra	0.869	0.956	0.913	6
CFU IFNg IL-21 Nos2 IL-2ra Tbet	0.846	0.979	0.913	6
CFU IFNg IL-21 LTA IL-18bp Tbet	0.844	0.983	0.913	6
CFU IFNg Gzmb Nos2 Socs1 IL-12rb2 IL-2ra	0.845	0.981	0.913	7
CFU IFNg IL-21 Gzmb Nos2 IL-12rb2 Tbet	0.849	0.977	0.913	7
CFU IFNg Gzmb Nos2 Socs1 IL-2ra Tbet	0.845	0.981	0.913	7
CFU IL-21 Gzmb Nos2 IL-12rb2 IL-2ra Tbet	0.862	0.964	0.913	7
CFU IFNg IL-21 Socs1 IL-12rb2 IL-18bp Tbet	0.838	0.988	0.913	7
CFU IL-21 Gzmb Nos2 IL-2ra IL-18bp Tbet	0.863	0.964	0.913	7
CFU IL-21 Gzmb Nos2 Socs1 IL-12rb2 IL-2ra Tbet	0.853	0.974	0.913	8
CFU IFNg IL-21 Nos2 Socs1 IL-12rb2 IL-18bp Tbet	0.839	0.987	0.913	8
CFU IL-21 Gzmb Nos2 Socs1 IL-12rb2 IL-18bp Tbet	0.856	0.971	0.913	8
CFU IFNg IL-21 Nos2 IL-12rb2 IL-2ra IL-18bp Tbet	0.846	0.98	0.913	8
CFU IL-21 Socs1 IL-18bp	0.868	0.957	0.912	4
CFU LTA Socs1 IL-18bp	0.863	0.96	0.912	4
CFU IFNg IL-18bp Tbet	0.841	0.983	0.912	4
CFU IL-21 LTA Socs1 IL-2ra	0.863	0.96	0.912	5
CFU Nos2 Socs1 IL-12rb2 IL-2ra	0.861	0.964	0.912	5
CFU IFNg LTA Gzmb IL-18bp	0.859	0.966	0.912	5
CFU Gzmb Socs1 IL-12rb2 IL-18bp	0.868	0.957	0.912	5

CFU LTA Socs1 IL-2ra IL-18bp	0.865	0.959	0.912	5
CFU IFNg Nos2 IL-2ra Tbet	0.844	0.98	0.912	5
CFU IL-21 IL-12rb2 IL-18bp Tbet	0.855	0.968	0.912	5
CFU IFNg LTA Nos2 IL-12rb2 IL-2ra	0.856	0.967	0.912	6
CFU IL-21 Gzmb Socs1 IL-12rb2 IL-2ra	0.87	0.954	0.912	6
CFU IFNg LTA Gzmb IL-12rb2 IL-18bp	0.859	0.965	0.912	6
CFU IFNg LTA IL-2ra IL-18bp Tbet	0.841	0.983	0.912	6
CFU IL-21 LTA Nos2 Socs1 IL-2ra Tbet	0.849	0.975	0.912	7
CFU IFNg Gzmb Socs1 IL-12rb2 IL-18bp Tbet	0.841	0.983	0.912	7
CFU IFNg IL-21 Gzmb IL-2ra IL-18bp Tbet	0.85	0.975	0.912	7
CFU IL-21 LTA Socs1 IL-2ra IL-18bp Tbet	0.858	0.966	0.912	7
CFU IFNg IL-21 Gzmb Nos2 Socs1 IL-12rb2 IL-18bp	0.838	0.986	0.912	8
CFU IFNg IL-21 Gzmb Nos2 Socs1 IL-2ra Tbet	0.843	0.981	0.912	8
CFU IFNg Gzmb Nos2 Socs1 IL-2ra IL-18bp Tbet	0.843	0.981	0.912	8
CFU IFNg IL-21 Gzmb IL-12rb2 IL-2ra IL-18bp Tbet	0.847	0.977	0.912	8
CFU IFNg Gzmb Socs1 IL-12rb2 IL-2ra IL-18bp Tbet	0.841	0.984	0.912	8
CFU IL-21 LTA Gzmb Nos2 Socs1 IL-12rb2 IL-18bp Tbet	0.857	0.968	0.912	9
CFU IFNg IL-21 Nos2 Socs1 IL-12rb2 IL-2ra IL-18bp Tbet	0.838	0.986	0.912	9
CFU IL-21 Nos2 Socs1	0.861	0.962	0.911	4
CFU LTA Nos2 Socs1	0.858	0.964	0.911	4
CFU IFNg IL-2ra Tbet	0.84	0.981	0.911	4
CFU IL-21 LTA Gzmb Socs1	0.864	0.958	0.911	5
CFU IL-21 Socs1 IL-12rb2 IL-18bp	0.867	0.956	0.911	5
CFU IL-21 Socs1 IL-2ra IL-18bp	0.866	0.956	0.911	5
CFU Gzmb Socs1 IL-2ra IL-18bp	0.866	0.957	0.911	5
CFU Nos2 Socs1 IL-2ra IL-18bp	0.86	0.962	0.911	5
CFU IFNg Gzmb Nos2 Tbet	0.847	0.976	0.911	5
CFU IFNg IL-21 IL-2ra Tbet	0.841	0.981	0.911	5
CFU Gzmb IL-2ra IL-18bp Tbet	0.868	0.954	0.911	5
CFU IFNg LTA Gzmb Nos2 IL-18bp	0.859	0.964	0.911	6
CFU Gzmb Socs1 IL-12rb2 IL-2ra IL-18bp	0.865	0.956	0.911	6
CFU IFNg IL-21 LTA IL-2ra Tbet	0.839	0.983	0.911	6
CFU IL-21 LTA Gzmb IL-18bp Tbet	0.86	0.962	0.911	6
CFU IFNg Gzmb Nos2 IL-18bp Tbet	0.846	0.976	0.911	6
CFU IFNg Nos2 IL-2ra IL-18bp Tbet	0.842	0.979	0.911	6
CFU IFNg LTA Gzmb Nos2 IL-12rb2 IL-18bp	0.858	0.963	0.911	7
CFU IFNg IL-21 Nos2 IL-2ra IL-18bp Tbet	0.844	0.978	0.911	7
CFU IFNg IL-21 Gzmb Nos2 IL-12rb2 IL-2ra Tbet	0.845	0.977	0.911	8
CFU IL-21 LTA Gzmb Socs1 IL-12rb2 IL-2ra Tbet	0.853	0.969	0.911	8
CFU IFNg IL-21 Gzmb Nos2 Socs1 IL-2ra IL-18bp Tbet	0.841	0.981	0.911	9
CFU IFNg IL-21 LTA Nos2	0.853	0.968	0.91	5
CFU IL-21 Nos2 Socs1 IL-12rb2	0.861	0.959	0.91	5
CFU IL-21 Nos2 Socs1 IL-2ra	0.858	0.962	0.91	5
CFU LTA Nos2 Socs1 IL-2ra	0.854	0.966	0.91	5
CFU IFNg Gzmb Socs1 IL-18bp	0.843	0.978	0.91	5
CFU Nos2 Socs1 IL-12rb2 IL-18bp	0.858	0.963	0.91	5
CFU IFNg IL-21 LTA Nos2 IL-12rb2	0.853	0.968	0.91	6
CFU IL-21 LTA Gzmb Socs1 IL-12rb2	0.865	0.954	0.91	6
CFU IFNg IL-21 LTA Nos2 IL-2ra	0.851	0.968	0.91	6
CFU IL-21 LTA Socs1 IL-12rb2 IL-2ra	0.863	0.958	0.91	6
CFU IFNg IL-21 LTA Nos2 IL-18bp	0.851	0.97	0.91	6
CFU IFNg Gzmb Socs1 IL-12rb2 IL-18bp	0.84	0.98	0.91	6
CFU IL-21 Socs1 IL-12rb2 IL-2ra IL-18bp	0.865	0.955	0.91	6
CFU IFNg Gzmb Nos2 IL-2ra Tbet	0.844	0.975	0.91	6
CFU IL-21 Nos2 IL-12rb2 IL-18bp Tbet	0.857	0.964	0.91	6
CFU Gzmb IL-12rb2 IL-2ra IL-18bp Tbet	0.865	0.954	0.91	6
CFU IFNg IL-21 LTA Nos2 IL-12rb2 IL-2ra	0.852	0.968	0.91	7

CFU IFNg IL-21 IL-12rb2 IL-2ra IL-18bp Tbet	0.837	0.983	0.91	7
CFU IFNg Gzmb Nos2 Socs1 IL-12rb2 IL-2ra IL-18bp	0.839	0.981	0.91	8
CFU IL-21 Nos2 Socs1 IL-12rb2 IL-2ra IL-18bp Tbet	0.849	0.971	0.91	8
CFU IFNg IL-21 Gzmb Nos2 IL-12rb2 IL-2ra IL-18bp Tbet	0.843	0.977	0.91	9
CFU IFNg IL-21 Gzmb Socs1 IL-12rb2 IL-2ra IL-18bp Tbet	0.836	0.984	0.91	9
CFU IFNg IL-21	0.845	0.972	0.909	3
CFU IL-21 Tbet	0.851	0.967	0.909	3
CFU IFNg IL-21 Gzmb	0.852	0.965	0.909	4
CFU IFNg Gzmb IL-2ra	0.855	0.962	0.909	4
CFU IFNg Gzmb IL-12rb2 IL-2ra	0.855	0.963	0.909	5
CFU LTA Gzmb Socs1 IL-18bp	0.863	0.956	0.909	5
CFU IFNg IL-2ra IL-18bp Tbet	0.837	0.981	0.909	5
CFU IL-12rb2 IL-2ra IL-18bp Tbet	0.859	0.959	0.909	5
CFU LTA Socs1 IL-12rb2 IL-2ra IL-18bp	0.861	0.958	0.909	6
CFU Nos2 Socs1 IL-12rb2 IL-2ra IL-18bp	0.857	0.961	0.909	6
CFU IFNg IL-21 Gzmb Nos2 Tbet	0.842	0.975	0.909	6
CFU IL-21 LTA Socs1 IL-12rb2 Tbet	0.845	0.973	0.909	6
CFU LTA Socs1 IL-12rb2 IL-2ra Tbet	0.846	0.973	0.909	6
CFU IFNg IL-21 IL-2ra IL-18bp Tbet	0.838	0.981	0.909	6
CFU IFNg IL-21 LTA Nos2 IL-12rb2 IL-18bp	0.85	0.969	0.909	7
CFU LTA Gzmb Socs1 IL-12rb2 IL-2ra Tbet	0.849	0.968	0.909	7
CFU IFNg IL-21 Gzmb Nos2 IL-18bp Tbet	0.842	0.975	0.909	7
CFU IFNg Gzmb Nos2 IL-2ra IL-18bp Tbet	0.844	0.975	0.909	7
CFU IL-21 LTA Gzmb Nos2 Socs1 IL-2ra Tbet	0.846	0.973	0.909	8
CFU IFNg IL-21 Gzmb Socs1 IL-12rb2 IL-18bp Tbet	0.833	0.985	0.909	8
CFU IL-21 LTA Nos2 Socs1 IL-2ra IL-18bp Tbet	0.854	0.964	0.909	8
CFU IFNg IL-21 LTA IL-12rb2 IL-2ra IL-18bp Tbet	0.833	0.984	0.909	8
CFU IFNg IL-21 IL-12rb2	0.844	0.972	0.908	4
CFU Gzmb IL-2ra Tbet	0.861	0.956	0.908	4
CFU IFNg IL-21 Nos2 IL-12rb2	0.847	0.969	0.908	5
CFU IFNg LTA Nos2 IL-18bp	0.847	0.97	0.908	5
CFU IL-21 Gzmb Socs1 IL-18bp	0.862	0.953	0.908	5
CFU IL-21 Nos2 Socs1 IL-18bp	0.858	0.957	0.908	5
CFU LTA Nos2 Socs1 IL-18bp	0.856	0.96	0.908	5
CFU LTA Socs1 IL-12rb2 IL-18bp	0.857	0.959	0.908	5
CFU IFNg IL-21 LTA Gzmb IL-12rb2	0.853	0.962	0.908	6
CFU IL-21 Nos2 Socs1 IL-12rb2 IL-2ra	0.857	0.96	0.908	6
CFU IFNg IL-21 LTA Gzmb IL-18bp	0.852	0.964	0.908	6
CFU IFNg LTA Nos2 IL-12rb2 IL-18bp	0.847	0.969	0.908	6
CFU LTA Gzmb Socs1 IL-12rb2 IL-18bp	0.862	0.954	0.908	6
CFU LTA Gzmb Socs1 IL-2ra IL-18bp	0.86	0.956	0.908	6
CFU LTA Nos2 Socs1 IL-12rb2 Tbet	0.84	0.975	0.908	6
CFU IFNg IL-21 LTA Nos2 IL-2ra IL-18bp	0.847	0.969	0.908	7
CFU IFNg IL-21 LTA IL-2ra IL-18bp Tbet	0.833	0.982	0.908	7
CFU IFNg IL-21 LTA Nos2 IL-12rb2 IL-2ra IL-18bp	0.848	0.968	0.908	8
CFU IL-21 Gzmb Nos2 Socs1 IL-12rb2 IL-2ra IL-18bp Tbet	0.843	0.972	0.908	9
CFU IL-2ra Tbet	0.852	0.961	0.907	3
CFU IFNg IL-12rb2 IL-2ra	0.842	0.972	0.907	4
CFU IL-21 IL-12rb2 Tbet	0.846	0.968	0.907	4
CFU IL-21 IL-18bp Tbet	0.851	0.963	0.907	4
CFU LTA Nos2 Socs1 IL-12rb2	0.853	0.962	0.907	5
CFU IFNg IL-21 LTA IL-18bp	0.838	0.976	0.907	5
CFU IFNg IL-21 Nos2 IL-18bp	0.842	0.972	0.907	5
CFU IL-21 LTA Socs1 IL-18bp	0.858	0.957	0.907	5
CFU Gzmb IL-12rb2 IL-2ra Tbet	0.86	0.954	0.907	5
CFU IL-21 LTA Gzmb Socs1 IL-2ra	0.857	0.957	0.907	6
CFU LTA Nos2 Socs1 IL-12rb2 IL-2ra	0.85	0.963	0.907	6

CFU IFNg IL-21 Nos2 IL-12rb2 IL-18bp	0.844	0.971	0.907	6
CFU IL-21 Gzmb Socs1 IL-12rb2 IL-18bp	0.862	0.953	0.907	6
CFU IL-21 Nos2 Socs1 IL-12rb2 IL-18bp	0.857	0.956	0.907	6
CFU LTA Nos2 Socs1 IL-2ra IL-18bp	0.853	0.962	0.907	6
CFU IL-21 LTA Gzmb Nos2 Tbet	0.85	0.964	0.907	6
CFU IFNg Gzmb Nos2 Socs1 Tbet	0.835	0.98	0.907	6
CFU IFNg IL-21 LTA Gzmb IL-12rb2 IL-2ra	0.856	0.959	0.907	7
CFU IFNg IL-21 LTA Gzmb IL-12rb2 IL-18bp	0.851	0.963	0.907	7
CFU LTA Gzmb Socs1 IL-12rb2 IL-2ra IL-18bp	0.859	0.954	0.907	7
CFU IFNg IL-21 Gzmb Nos2 Socs1 Tbet	0.832	0.981	0.907	7
CFU IFNg IL-21 Gzmb Nos2 IL-2ra Tbet	0.839	0.975	0.907	7
CFU IL-21 LTA Gzmb Nos2 IL-18bp Tbet	0.853	0.962	0.907	7
CFU IFNg IL-21 Gzmb Nos2 Socs1 IL-18bp Tbet	0.832	0.981	0.907	8
CFU IFNg	0.837	0.975	0.906	2
CFU Gzmb Nos2 Socs1	0.852	0.96	0.906	4
CFU IFNg IL-21 IL-18bp	0.837	0.975	0.906	4
CFU IL-2ra IL-18bp Tbet	0.855	0.958	0.906	4
CFU IFNg IL-21 LTA IL-12rb2	0.84	0.973	0.906	5
CFU IFNg IL-21 IL-12rb2 IL-2ra	0.839	0.972	0.906	5
CFU IFNg IL-21 IL-12rb2 IL-18bp	0.838	0.974	0.906	5
CFU IFNg IL-21 Nos2 IL-12rb2 IL-2ra	0.842	0.969	0.906	6
CFU IFNg IL-21 LTA IL-12rb2 IL-18bp	0.837	0.975	0.906	6
CFU IFNg LTA Gzmb IL-2ra IL-18bp	0.855	0.956	0.906	6
CFU IFNg LTA Nos2 IL-2ra IL-18bp	0.842	0.969	0.906	6
CFU IL-21 Gzmb Socs1 IL-2ra IL-18bp	0.86	0.953	0.906	6
CFU IL-21 Nos2 Socs1 IL-2ra IL-18bp	0.855	0.958	0.906	6
CFU IL-21 LTA Gzmb IL-2ra Tbet	0.85	0.963	0.906	6
CFU Gzmb Nos2 IL-2ra IL-18bp Tbet	0.86	0.952	0.906	6
CFU IL-21 LTA Gzmb Socs1 IL-12rb2 IL-2ra	0.857	0.954	0.906	7
CFU IFNg IL-21 LTA Gzmb Nos2 IL-18bp	0.851	0.962	0.906	7
CFU IFNg LTA Gzmb Nos2 IL-2ra IL-18bp	0.855	0.956	0.906	7
CFU IL-21 Gzmb Socs1 IL-12rb2 IL-2ra IL-18bp	0.859	0.953	0.906	7
CFU IFNg Gzmb Nos2 Socs1 IL-12rb2 Tbet	0.829	0.983	0.906	7
CFU IL-21 LTA Socs1 IL-12rb2 IL-2ra Tbet	0.838	0.973	0.906	7
CFU IL-21 LTA Socs1 IL-12rb2 IL-18bp Tbet	0.842	0.97	0.906	7
CFU IFNg IL-21 Gzmb Nos2 Socs1 IL-12rb2 Tbet	0.827	0.985	0.906	8
CFU IFNg Gzmb Nos2 Socs1 IL-12rb2 IL-2ra Tbet	0.829	0.983	0.906	8
CFU IFNg IL-21 Gzmb Nos2 IL-2ra IL-18bp Tbet	0.838	0.975	0.906	8
CFU IFNg IL-21 Gzmb Nos2 Socs1 IL-12rb2 IL-2ra Tbet	0.827	0.984	0.906	9
CFU IL-18bp Tbet	0.856	0.953	0.905	3
CFU IFNg IL-21 Nos2	0.84	0.969	0.905	4
CFU IFNg IL-21 IL-2ra	0.837	0.972	0.905	4
CFU IFNg LTA IL-18bp	0.833	0.977	0.905	4
CFU IL-21 IL-2ra Tbet	0.844	0.966	0.905	4
CFU IL-21 LTA Nos2 Socs1	0.847	0.962	0.905	5
CFU IFNg IL-21 Gzmb IL-12rb2	0.845	0.966	0.905	5
CFU IFNg IL-21 Gzmb IL-2ra	0.844	0.965	0.905	5
CFU Gzmb Nos2 Socs1 IL-2ra	0.849	0.96	0.905	5
CFU IFNg IL-21 Gzmb IL-18bp	0.843	0.967	0.905	5
CFU IFNg IL-21 LTA Gzmb Nos2	0.848	0.962	0.905	6
CFU IFNg IL-21 LTA IL-12rb2 IL-2ra	0.837	0.973	0.905	6
CFU IL-21 LTA Socs1 IL-12rb2 IL-18bp	0.854	0.956	0.905	6
CFU IL-21 LTA Gzmb IL-12rb2 Tbet	0.848	0.963	0.905	6
CFU Nos2 IL-12rb2 IL-2ra IL-18bp Tbet	0.856	0.954	0.905	6
CFU IFNg LTA Gzmb IL-12rb2 IL-2ra IL-18bp	0.855	0.956	0.905	7
CFU IFNg IL-21 Nos2 IL-12rb2 IL-2ra IL-18bp	0.839	0.971	0.905	7
CFU IFNg LTA Nos2 IL-12rb2 IL-2ra IL-18bp	0.843	0.967	0.905	7

CFU IL-21 Nos2 Socs1 IL-12rb2 IL-2ra IL-18bp	0.853	0.957	0.905	7
CFU IFNg Gzmb Nos2 Socs1 IL-18bp Tbet	0.83	0.981	0.905	7
CFU IL-21 LTA Gzmb IL-12rb2 IL-18bp Tbet	0.845	0.964	0.905	7
CFU Gzmb Nos2 IL-12rb2 IL-2ra IL-18bp Tbet	0.859	0.952	0.905	7
CFU IFNg IL-21 LTA Gzmb Nos2 IL-12rb2 IL-18bp	0.848	0.962	0.905	8
CFU IFNg LTA Gzmb Nos2 IL-12rb2 IL-2ra IL-18bp	0.854	0.955	0.905	8
CFU IFNg Gzmb Nos2 Socs1 IL-12rb2 IL-18bp Tbet	0.827	0.983	0.905	8
CFU IFNg IL-21 Gzmb Nos2 Socs1 IL-12rb2 IL-18bp Tbet	0.825	0.984	0.905	9
CFU IL-12rb2 IL-2ra Tbet	0.848	0.96	0.904	4
CFU Gzmb IL-18bp Tbet	0.863	0.946	0.904	4
CFU Gzmb Nos2 Socs1 IL-12rb2	0.85	0.958	0.904	5
CFU Gzmb Nos2 Socs1 IL-18bp	0.85	0.958	0.904	5
CFU IFNg LTA IL-12rb2 IL-18bp	0.832	0.976	0.904	5
CFU IL-21 IL-12rb2 IL-2ra Tbet	0.842	0.967	0.904	5
CFU IFNg IL-21 Gzmb IL-12rb2 IL-18bp	0.841	0.967	0.904	6
CFU IFNg IL-21 Nos2 IL-2ra IL-18bp	0.836	0.973	0.904	6
CFU IFNg IL-21 LTA Gzmb Nos2 IL-12rb2	0.846	0.961	0.904	7
CFU IFNg IL-21 LTA Gzmb Nos2 IL-2ra	0.848	0.959	0.904	7
CFU LTA Gzmb Nos2 Socs1 IL-12rb2 IL-18bp Tbet	0.844	0.965	0.904	8
CFU IL-21 LTA Gzmb Socs1 IL-12rb2 IL-2ra IL-18bp Tbet	0.837	0.97	0.904	9
CFU IFNg Gzmb Nos2 Socs1 IL-12rb2 IL-2ra IL-18bp Tbet	0.824	0.983	0.904	9
CFU IFNg IL-2ra	0.834	0.973	0.903	3
CFU IFNg Gzmb Nos2	0.844	0.962	0.903	4
CFU IFNg Gzmb IL-18bp	0.84	0.965	0.903	4
CFU IL-12rb2 IL-18bp Tbet	0.856	0.951	0.903	4
CFU IFNg IL-21 IL-2ra IL-18bp	0.83	0.976	0.903	5
CFU Gzmb Nos2 Socs1 IL-12rb2 IL-2ra	0.847	0.959	0.903	6
CFU IFNg IL-21 Gzmb Nos2 IL-18bp	0.839	0.966	0.903	6
CFU LTA Nos2 Socs1 IL-12rb2 IL-18bp	0.848	0.959	0.903	6
CFU IFNg IL-21 LTA IL-2ra IL-18bp	0.831	0.975	0.903	6
CFU IL-21 LTA Socs1 IL-2ra IL-18bp	0.849	0.957	0.903	6
CFU IFNg IL-21 IL-12rb2 IL-2ra IL-18bp	0.832	0.975	0.903	6
CFU IL-21 IL-12rb2 IL-2ra IL-18bp Tbet	0.839	0.967	0.903	6
CFU IFNg IL-21 LTA IL-12rb2 IL-2ra IL-18bp	0.832	0.974	0.903	7
CFU LTA Nos2 Socs1 IL-12rb2 IL-2ra IL-18bp	0.846	0.96	0.903	7
CFU IL-21 LTA Gzmb IL-12rb2 IL-2ra Tbet	0.844	0.961	0.903	7
CFU IL-21 Nos2 IL-12rb2 IL-2ra IL-18bp Tbet	0.842	0.963	0.903	7
CFU IFNg IL-21 LTA Gzmb Nos2 IL-12rb2 IL-2ra	0.846	0.959	0.903	8
CFU IL-21 LTA Nos2 Socs1 IL-12rb2 IL-18bp Tbet	0.84	0.967	0.903	8
CFU LTA Gzmb Nos2 Socs1 IL-2ra IL-18bp Tbet	0.841	0.964	0.903	8
CFU IFNg IL-21 Gzmb Nos2 Socs1 IL-12rb2 IL-2ra IL-18bp Tbet	0.822	0.984	0.903	10
CFU IFNg Nos2	0.834	0.971	0.902	3
CFU LTA Tbet	0.837	0.966	0.902	3
CFU IL-21 Gzmb Nos2 Socs1	0.847	0.957	0.902	5
CFU IFNg IL-21 Nos2 IL-2ra	0.834	0.969	0.902	5
CFU IFNg Gzmb IL-12rb2 IL-18bp	0.838	0.965	0.902	5
CFU Gzmb Nos2 IL-2ra Tbet	0.849	0.955	0.902	5
CFU IL-21 Nos2 IL-18bp Tbet	0.845	0.959	0.902	5
CFU IFNg IL-21 Gzmb Nos2 IL-12rb2	0.839	0.965	0.902	6
CFU IL-21 LTA Nos2 Socs1 IL-12rb2	0.844	0.959	0.902	6
CFU IL-21 LTA Nos2 Socs1 IL-2ra	0.841	0.964	0.902	6
CFU IFNg IL-21 Gzmb IL-12rb2 IL-2ra	0.839	0.966	0.902	6
CFU IL-21 LTA Gzmb Socs1 IL-18bp	0.85	0.954	0.902	6
CFU Gzmb Nos2 Socs1 IL-12rb2 IL-18bp	0.847	0.958	0.902	6
CFU Gzmb Nos2 Socs1 IL-2ra IL-18bp	0.846	0.958	0.902	6
CFU IFNg IL-21 LTA Gzmb IL-2ra IL-18bp	0.847	0.957	0.902	7
CFU LTA Nos2 Socs1 IL-12rb2 IL-2ra Tbet	0.829	0.976	0.902	7

CFU IFNg IL-21 LTA Gzmb Nos2 IL-2ra IL-18bp	0.848	0.957	0.902	8
CFU IL-21 LTA Gzmb Nos2 Socs1 IL-12rb2 Tbet	0.833	0.971	0.902	8
CFU Gzmb IL-12rb2 IL-18bp Tbet	0.857	0.945	0.901	5
CFU Nos2 IL-2ra IL-18bp Tbet	0.848	0.954	0.901	5
CFU IL-21 Gzmb Nos2 Socs1 IL-12rb2	0.846	0.955	0.901	6
CFU IL-21 Gzmb Nos2 Socs1 IL-2ra	0.844	0.958	0.901	6
CFU IFNg IL-21 Gzmb IL-2ra IL-18bp	0.835	0.967	0.901	6
CFU IFNg IL-21 Gzmb Nos2 IL-12rb2 IL-18bp	0.836	0.966	0.901	7
CFU IFNg IL-21 Gzmb IL-12rb2 IL-2ra IL-18bp	0.834	0.967	0.901	7
CFU LTA Gzmb Nos2 Socs1 IL-2ra Tbet	0.83	0.972	0.901	7
CFU IL-21 LTA Gzmb IL-2ra IL-18bp Tbet	0.841	0.961	0.901	7
CFU IFNg IL-21 LTA Gzmb IL-12rb2 IL-2ra IL-18bp	0.846	0.955	0.901	8
CFU Tbet	0.85	0.95	0.9	2
CFU IFNg Nos2 IL-12rb2	0.831	0.969	0.9	4
CFU Gzmb IL-12rb2 Tbet	0.855	0.945	0.9	4
CFU LTA Gzmb Nos2 Socs1	0.84	0.959	0.9	5
CFU IL-21 LTA Nos2 Socs1 IL-18bp	0.843	0.958	0.9	6
CFU IFNg Gzmb Nos2 Socs1 IL-18bp	0.823	0.978	0.9	6
CFU Gzmb Nos2 IL-12rb2 IL-2ra Tbet	0.847	0.953	0.9	6
CFU IFNg IL-21 Gzmb Nos2 IL-12rb2 IL-2ra	0.834	0.966	0.9	7
CFU IL-21 LTA Gzmb Socs1 IL-12rb2 IL-18bp	0.848	0.952	0.9	7
CFU IFNg Gzmb Nos2 Socs1 IL-12rb2 IL-18bp	0.82	0.979	0.9	7
CFU IL-21 LTA Socs1 IL-12rb2 IL-2ra IL-18bp	0.845	0.955	0.9	7
CFU Gzmb Nos2 Socs1 IL-12rb2 IL-2ra IL-18bp	0.844	0.957	0.9	7
CFU IL-21 LTA Nos2 Socs1 IL-12rb2 Tbet	0.826	0.975	0.9	7
CFU LTA Socs1 IL-12rb2 IL-2ra IL-18bp Tbet	0.832	0.969	0.9	7
CFU IL-21 LTA Gzmb Nos2 IL-12rb2 IL-18bp Tbet	0.838	0.963	0.9	8
CFU IFNg IL-21 LTA Gzmb Nos2 IL-12rb2 IL-2ra IL-18bp	0.845	0.955	0.9	9
CFU LTA Gzmb Tbet	0.835	0.962	0.899	4
CFU IL-21 Nos2 Tbet	0.832	0.966	0.899	4
CFU IFNg IL-21 Gzmb Nos2	0.834	0.965	0.899	5
CFU IFNg Gzmb Nos2 IL-12rb2	0.835	0.962	0.899	5
CFU IFNg Gzmb Nos2 IL-18bp	0.833	0.964	0.899	5
CFU IFNg Gzmb IL-2ra IL-18bp	0.833	0.965	0.899	5
CFU Gzmb Nos2 IL-18bp Tbet	0.853	0.944	0.899	5
CFU IL-21 IL-2ra IL-18bp Tbet	0.836	0.962	0.899	5
CFU LTA Gzmb Nos2 Socs1 IL-12rb2	0.841	0.956	0.899	6
CFU IL-21 Gzmb Nos2 Socs1 IL-18bp	0.845	0.953	0.899	6
CFU IFNg LTA IL-12rb2 IL-2ra IL-18bp	0.823	0.974	0.899	6
CFU IL-21 LTA Nos2 Socs1 IL-12rb2 IL-2ra	0.838	0.961	0.899	7
CFU IL-21 Gzmb Nos2 Socs1 IL-12rb2 IL-2ra	0.843	0.956	0.899	7
CFU IFNg IL-21 Gzmb Nos2 IL-2ra IL-18bp	0.832	0.966	0.899	7
CFU IL-21 LTA Gzmb Nos2 IL-12rb2 Tbet	0.834	0.963	0.899	7
CFU LTA Gzmb Nos2 Socs1 IL-12rb2 Tbet	0.827	0.971	0.899	7
CFU IL-21 LTA Gzmb Nos2 IL-2ra Tbet	0.835	0.962	0.899	7
CFU IFNg IL-21 Gzmb Nos2 IL-12rb2 IL-2ra IL-18bp	0.831	0.966	0.899	8
CFU IL-12rb2 Tbet	0.845	0.951	0.898	3
CFU Nos2 IL-2ra Tbet	0.835	0.961	0.898	4
CFU Nos2 IL-18bp Tbet	0.848	0.949	0.898	4
CFU IFNg Nos2 IL-12rb2 IL-18bp	0.825	0.971	0.898	5
CFU IFNg LTA IL-2ra IL-18bp	0.822	0.975	0.898	5
CFU IL-21 Nos2 IL-12rb2 Tbet	0.829	0.968	0.898	5
CFU LTA Gzmb IL-2ra Tbet	0.834	0.961	0.898	5
CFU LTA Gzmb Nos2 Socs1 IL-2ra	0.835	0.962	0.898	6
CFU IFNg Gzmb IL-12rb2 IL-2ra IL-18bp	0.832	0.964	0.898	6
CFU IFNg IL-12rb2 IL-18bp	0.819	0.975	0.897	4
CFU IFNg Nos2 IL-12rb2 IL-2ra	0.825	0.968	0.897	5

CFU Nos2 IL-12rb2 IL-18bp Tbet	0.848	0.945	0.897	5
CFU LTA Gzmb Nos2 Socs1 IL-18bp	0.838	0.956	0.897	6
CFU IFNg Gzmb Nos2 IL-12rb2 IL-18bp	0.83	0.964	0.897	6
CFU LTA Gzmb Nos2 Socs1 IL-12rb2 IL-2ra	0.837	0.958	0.897	7
CFU IL-21 LTA Nos2 Socs1 IL-12rb2 IL-18bp	0.837	0.957	0.897	7
CFU IL-21 Gzmb Nos2 Socs1 IL-12rb2 IL-18bp	0.842	0.952	0.897	7
CFU IL-21 Gzmb Nos2 Socs1 IL-2ra IL-18bp	0.841	0.953	0.897	7
CFU IL-21 LTA Gzmb IL-12rb2 IL-2ra IL-18bp Tbet	0.83	0.963	0.897	8
CFU LTA Gzmb Socs1 IL-12rb2 IL-2ra IL-18bp Tbet	0.828	0.966	0.897	8
CFU LTA Nos2 Socs1 IL-12rb2 IL-2ra IL-18bp Tbet	0.826	0.967	0.897	8
CFU LTA IL-12rb2 Tbet	0.824	0.968	0.896	4
CFU IL-21 LTA IL-12rb2 Tbet	0.821	0.971	0.896	5
CFU LTA Gzmb IL-12rb2 Tbet	0.831	0.962	0.896	5
CFU Nos2 IL-12rb2 IL-2ra Tbet	0.832	0.959	0.896	5
CFU IL-21 LTA Gzmb Nos2 Socs1	0.835	0.958	0.896	6
CFU IFNg IL-21 Gzmb Nos2 IL-2ra	0.828	0.965	0.896	6
CFU IFNg Nos2 IL-12rb2 IL-2ra IL-18bp	0.821	0.97	0.896	6
CFU LTA Gzmb IL-12rb2 IL-2ra Tbet	0.832	0.961	0.896	6
CFU IL-21 Nos2 IL-12rb2 IL-2ra Tbet	0.825	0.966	0.896	6
CFU Gzmb Nos2 IL-12rb2 IL-18bp Tbet	0.849	0.942	0.896	6
CFU LTA Gzmb Nos2 Socs1 IL-12rb2 IL-18bp	0.837	0.954	0.896	7
CFU IL-21 Gzmb Nos2 Socs1 IL-12rb2 IL-2ra IL-18bp	0.839	0.953	0.896	8
CFU IL-21 LTA Gzmb Nos2 IL-2ra IL-18bp Tbet	0.832	0.961	0.896	8
CFU Gzmb Tbet	0.844	0.947	0.895	3
CFU IFNg Nos2 IL-18bp	0.815	0.974	0.895	4
CFU IL-21 LTA Tbet	0.821	0.968	0.895	4
CFU IFNg IL-12rb2 IL-2ra IL-18bp	0.815	0.974	0.895	5
CFU IL-21 Nos2 IL-2ra Tbet	0.826	0.965	0.895	5
CFU LTA IL-12rb2 IL-2ra Tbet	0.822	0.968	0.895	5
CFU IFNg Gzmb Nos2 IL-2ra IL-18bp	0.826	0.963	0.895	6
CFU IL-21 LTA Gzmb Nos2 Socs1 IL-12rb2	0.836	0.955	0.895	7
CFU IL-21 LTA Gzmb Socs1 IL-2ra IL-18bp	0.836	0.954	0.895	7
CFU IL-21 LTA Nos2 Socs1 IL-2ra IL-18bp	0.831	0.959	0.895	7
CFU LTA Gzmb Nos2 Socs1 IL-2ra IL-18bp	0.833	0.958	0.895	7
CFU IL-21 LTA Gzmb Nos2 IL-12rb2 IL-2ra Tbet	0.828	0.961	0.895	8
CFU IFNg IL-18bp	0.811	0.978	0.894	3
CFU LTA IL-2ra Tbet	0.822	0.966	0.894	4
CFU IFNg Gzmb Nos2 IL-12rb2 IL-2ra	0.826	0.962	0.894	6
CFU IL-21 LTA Nos2 IL-12rb2 Tbet	0.816	0.971	0.894	6
CFU IL-21 Nos2 IL-2ra IL-18bp Tbet	0.831	0.958	0.894	6
CFU IL-21 LTA Gzmb Nos2 Socs1 IL-2ra	0.83	0.959	0.894	7
CFU LTA Gzmb Nos2 Socs1 IL-12rb2 IL-2ra IL-18bp	0.833	0.956	0.894	8
CFU IL-21 LTA Gzmb Nos2 Socs1 IL-12rb2 IL-2ra IL-18bp Tbet	0.819	0.97	0.894	10
CFU LTA Nos2 Tbet	0.821	0.966	0.893	4
CFU LTA IL-18bp Tbet	0.824	0.961	0.893	4
CFU IFNg Gzmb Nos2 IL-2ra	0.825	0.962	0.893	5
CFU IFNg Nos2 IL-2ra IL-18bp	0.813	0.972	0.893	5
CFU LTA Nos2 IL-12rb2 Tbet	0.818	0.969	0.893	5
CFU Gzmb Nos2 IL-12rb2 Tbet	0.841	0.945	0.893	5
CFU LTA Gzmb IL-18bp Tbet	0.829	0.957	0.893	5
CFU LTA IL-12rb2 IL-18bp Tbet	0.82	0.966	0.893	5
CFU IL-21 LTA IL-12rb2 IL-2ra Tbet	0.817	0.97	0.893	6
CFU LTA Nos2 IL-12rb2 IL-2ra Tbet	0.818	0.968	0.893	6
CFU LTA Gzmb IL-2ra IL-18bp Tbet	0.829	0.957	0.893	6
CFU IFNg Gzmb Nos2 IL-12rb2 IL-2ra IL-18bp	0.824	0.963	0.893	7
CFU IL-21 LTA Gzmb Nos2 Socs1 IL-12rb2 IL-2ra	0.83	0.956	0.893	8
CFU IL-21 LTA Gzmb Socs1 IL-12rb2 IL-2ra IL-18bp	0.833	0.953	0.893	8

CFU IL-21 LTA Nos2 Socs1 IL-12rb2 IL-2ra Tbet	0.81	0.976	0.893	8
CFU IL-21 LTA Gzmb Nos2 Socs1 IL-12rb2 IL-2ra Tbet	0.815	0.972	0.893	9
CFU IL-21 LTA Gzmb Nos2 IL-12rb2 IL-2ra IL-18bp Tbet	0.823	0.962	0.893	9
CFU Nos2 Tbet	0.832	0.952	0.892	3
CFU IFNg IL-2ra IL-18bp	0.809	0.976	0.892	4
CFU IL-21 LTA Nos2 Tbet	0.816	0.968	0.892	5
CFU LTA Nos2 IL-2ra Tbet	0.818	0.965	0.892	5
CFU LTA Gzmb IL-12rb2 IL-18bp Tbet	0.823	0.961	0.892	6
CFU LTA IL-12rb2 IL-2ra IL-18bp Tbet	0.817	0.966	0.892	6
CFU LTA Gzmb IL-12rb2 IL-2ra IL-18bp Tbet	0.823	0.961	0.892	7
CFU IL-21 LTA Nos2 Socs1 IL-12rb2 IL-2ra IL-18bp	0.826	0.958	0.892	8
CFU IFNg Nos2 IL-2ra	0.813	0.969	0.891	4
CFU Gzmb Nos2 Tbet	0.834	0.948	0.891	4
CFU IL-21 LTA IL-12rb2 IL-18bp Tbet	0.813	0.97	0.891	6
CFU LTA Nos2 IL-12rb2 IL-18bp Tbet	0.821	0.961	0.891	6
CFU IL-21 LTA Gzmb Nos2 Socs1 IL-18bp	0.829	0.953	0.891	7
CFU IL-21 LTA Nos2 IL-12rb2 IL-18bp Tbet	0.818	0.965	0.891	7
CFU LTA Nos2 IL-12rb2 IL-2ra IL-18bp Tbet	0.82	0.961	0.891	7
CFU LTA Gzmb Nos2 Socs1 IL-12rb2 IL-2ra Tbet	0.81	0.972	0.891	8
CFU Nos2 IL-12rb2 Tbet	0.829	0.952	0.89	4
CFU IL-21 LTA IL-2ra Tbet	0.813	0.967	0.89	5
CFU IL-21 LTA Nos2 IL-12rb2 IL-2ra Tbet	0.811	0.97	0.89	7
CFU IL-21 LTA Gzmb Nos2 Socs1 IL-12rb2 IL-18bp	0.828	0.952	0.89	8
CFU LTA Gzmb Nos2 Tbet	0.814	0.963	0.889	5
CFU LTA Nos2 IL-18bp Tbet	0.822	0.956	0.889	5
CFU LTA Gzmb Nos2 IL-2ra Tbet	0.817	0.962	0.889	6
CFU IL-21 LTA Nos2 IL-2ra Tbet	0.809	0.967	0.888	6
CFU LTA Gzmb Nos2 IL-12rb2 IL-2ra Tbet	0.815	0.962	0.888	7
CFU LTA Gzmb Nos2 IL-12rb2 IL-2ra IL-18bp Tbet	0.817	0.959	0.888	8
CFU LTA IL-2ra IL-18bp Tbet	0.815	0.96	0.887	5
CFU LTA Gzmb Nos2 IL-12rb2 Tbet	0.811	0.963	0.887	6
CFU LTA Gzmb Nos2 IL-18bp Tbet	0.817	0.957	0.887	6
CFU LTA Nos2 IL-2ra IL-18bp Tbet	0.818	0.955	0.887	6
CFU LTA Gzmb Nos2 IL-12rb2 IL-18bp Tbet	0.815	0.96	0.887	7
CFU LTA Gzmb Nos2 IL-2ra IL-18bp Tbet	0.819	0.956	0.887	7
CFU LTA Gzmb Nos2 Socs1 IL-12rb2 IL-2ra IL-18bp Tbet	0.809	0.966	0.887	9
CFU IL-21 LTA IL-18bp Tbet	0.81	0.963	0.886	5
CFU IL-21 LTA Nos2 IL-18bp Tbet	0.814	0.958	0.886	6
CFU IL-21 LTA Gzmb Nos2 Socs1 IL-2ra IL-18bp	0.816	0.955	0.885	8
CFU IL-21 LTA Gzmb Nos2 Socs1 IL-12rb2 IL-2ra IL-18bp	0.817	0.954	0.885	9
CFU IL-21 LTA IL-12rb2 IL-2ra IL-18bp Tbet	0.798	0.969	0.884	7
CFU IL-21 LTA Nos2 IL-12rb2 IL-2ra IL-18bp Tbet	0.804	0.964	0.884	8
CFU IL-21 LTA Socs1 IL-12rb2 IL-2ra IL-18bp Tbet	0.797	0.971	0.884	8
CFU IL-21 LTA Nos2 Socs1 IL-12rb2 IL-2ra IL-18bp Tbet	0.793	0.969	0.881	9
CFU IL-21 LTA IL-2ra IL-18bp Tbet	0.796	0.962	0.879	6
CFU IL-21 LTA Nos2 IL-2ra IL-18bp Tbet	0.801	0.957	0.879	7
CFU LTA	0.795	0.936	0.866	2
CFU LTA Gzmb	0.793	0.932	0.863	3
CFU LTA IL-12rb2	0.792	0.934	0.863	3
CFU LTA Nos2	0.788	0.935	0.862	3
CFU LTA IL-2ra	0.793	0.932	0.862	3
CFU LTA Gzmb IL-12rb2	0.794	0.929	0.861	4
CFU LTA Gzmb IL-2ra	0.795	0.925	0.86	4
CFU LTA Nos2 IL-2ra	0.79	0.931	0.86	4
CFU LTA IL-12rb2 IL-2ra	0.79	0.931	0.86	4
CFU LTA Nos2 IL-12rb2	0.786	0.933	0.859	4
CFU LTA Gzmb IL-12rb2 IL-2ra	0.795	0.923	0.859	5

CFU LTA Nos2 IL-12rb2 IL-2ra	0.788	0.928	0.858	5
CFU IL-21 LTA	0.779	0.935	0.857	3
CFU LTA Gzmb Nos2	0.78	0.932	0.856	4
CFU IL-21 Gzmb IL-12rb2	0.795	0.917	0.856	4
CFU IL-21 LTA IL-2ra	0.782	0.93	0.856	4
CFU IL-21 LTA Gzmb	0.78	0.931	0.855	4
CFU LTA Gzmb Nos2 IL-12rb2	0.782	0.929	0.855	5
CFU LTA Gzmb Nos2 IL-2ra	0.786	0.925	0.855	5
CFU IL-21 Gzmb IL-12rb2 IL-18bp	0.793	0.918	0.855	5
CFU IL-21 LTA IL-12rb2	0.776	0.933	0.854	4
CFU Gzmb IL-12rb2 IL-2ra	0.798	0.911	0.854	4
CFU IL-21 LTA Gzmb IL-12rb2	0.78	0.928	0.854	5
CFU IL-21 LTA Gzmb IL-2ra	0.784	0.924	0.854	5
CFU IL-21 LTA IL-12rb2 IL-2ra	0.779	0.929	0.854	5
CFU Gzmb IL-12rb2 IL-2ra IL-18bp	0.797	0.91	0.854	5
CFU LTA Gzmb Nos2 IL-12rb2 IL-2ra	0.787	0.922	0.854	6
CFU IL-21 Gzmb	0.795	0.912	0.853	3
CFU IL-21 Gzmb IL-12rb2 IL-2ra	0.794	0.912	0.853	5
CFU IL-21 LTA Gzmb IL-12rb2 IL-2ra	0.785	0.921	0.853	6
CFU IL-21 Gzmb IL-12rb2 IL-2ra IL-18bp	0.793	0.912	0.853	6
CFU Nos2 IL-12rb2 IL-2ra	0.789	0.915	0.852	4
CFU IL-21 Gzmb Nos2 IL-12rb2	0.789	0.916	0.852	5
CFU Gzmb Nos2 IL-12rb2 IL-2ra	0.795	0.909	0.852	5
CFU IL-21 Gzmb Nos2 IL-12rb2 IL-18bp	0.788	0.916	0.852	6
CFU IL-21	0.785	0.916	0.851	2
CFU IL-12rb2 IL-2ra	0.786	0.917	0.851	3
CFU LTA IL-18bp	0.767	0.936	0.851	3
CFU IL-21 Gzmb Nos2	0.791	0.911	0.851	4
CFU IL-21 Gzmb IL-2ra	0.794	0.908	0.851	4
CFU LTA IL-12rb2 IL-18bp	0.766	0.935	0.851	4
CFU IL-21 LTA Nos2 IL-2ra	0.773	0.929	0.851	5
CFU IL-2ra	0.789	0.911	0.85	2
CFU IL-21 IL-12rb2	0.779	0.92	0.85	3
CFU Gzmb IL-12rb2	0.79	0.909	0.85	3
CFU Gzmb IL-2ra	0.793	0.906	0.85	3
CFU Nos2 IL-2ra	0.791	0.909	0.85	3
CFU IL-21 LTA Nos2	0.766	0.935	0.85	4
CFU IL-21 Gzmb IL-18bp	0.79	0.911	0.85	4
CFU IL-21 Gzmb Nos2 IL-12rb2 IL-2ra	0.79	0.91	0.85	6
CFU Gzmb Nos2 IL-12rb2 IL-2ra IL-18bp	0.792	0.908	0.85	6
CFU IL-21 Nos2	0.783	0.915	0.849	3
CFU IL-21 IL-2ra	0.786	0.911	0.849	3
CFU IL-21 Nos2 IL-12rb2	0.779	0.918	0.849	4
CFU IL-21 Nos2 IL-2ra	0.788	0.909	0.849	4
CFU LTA Nos2 IL-18bp	0.762	0.937	0.849	4
CFU IL-21 Gzmb Nos2 IL-2ra	0.791	0.906	0.849	5
CFU IL-21 Nos2 IL-12rb2 IL-2ra	0.786	0.913	0.849	5
CFU IL-21 LTA Nos2 IL-12rb2 IL-2ra	0.772	0.927	0.849	6
CFU IL-21 Gzmb Nos2 IL-12rb2 IL-2ra IL-18bp	0.788	0.91	0.849	7
CFU IL-12rb2	0.784	0.913	0.848	2
CFU Gzmb Nos2 IL-2ra	0.793	0.904	0.848	4
CFU IL-21 IL-12rb2 IL-2ra	0.782	0.915	0.848	4
CFU Gzmb IL-12rb2 IL-18bp	0.788	0.908	0.848	4
CFU IL-21 LTA Nos2 IL-12rb2	0.765	0.932	0.848	5
CFU IL-21 Gzmb Nos2 IL-18bp	0.787	0.909	0.848	5
CFU LTA Nos2 IL-12rb2 IL-18bp	0.762	0.935	0.848	5
CFU IL-21 Gzmb IL-2ra IL-18bp	0.789	0.906	0.848	5

CFU Nos2 IL-12rb2	0.782	0.912	0.847	3
CFU IL-21 IL-18bp	0.779	0.915	0.847	3
CFU Gzmb Nos2 IL-12rb2	0.786	0.908	0.847	4
CFU IL-12rb2 IL-18bp	0.778	0.913	0.846	3
CFU IL-2ra IL-18bp	0.783	0.909	0.846	3
CFU LTA Gzmb IL-18bp	0.759	0.933	0.846	4
CFU IL-21 Nos2 IL-18bp	0.779	0.913	0.846	4
CFU LTA IL-2ra IL-18bp	0.76	0.932	0.846	4
CFU Gzmb IL-2ra IL-18bp	0.789	0.904	0.846	4
CFU Nos2 IL-2ra IL-18bp	0.786	0.906	0.846	4
CFU IL-12rb2 IL-2ra IL-18bp	0.776	0.917	0.846	4
IL-21 IL-12rb2 IL-18bp Tbet	0.775	0.916	0.846	4
CFU LTA Gzmb IL-12rb2 IL-18bp	0.762	0.93	0.846	5
CFU Nos2 IL-12rb2 IL-2ra IL-18bp	0.778	0.914	0.846	5
CFU IL-21 IL-12rb2 IL-18bp	0.77	0.921	0.845	4
CFU IL-21 LTA Gzmb Nos2	0.758	0.931	0.845	5
CFU Gzmb Nos2 IL-12rb2 IL-18bp	0.784	0.907	0.845	5
CFU LTA Nos2 IL-2ra IL-18bp	0.758	0.931	0.845	5
CFU Gzmb Nos2 IL-2ra IL-18bp	0.788	0.902	0.845	5
IL-21 Nos2 IL-12rb2 IL-18bp Tbet	0.767	0.922	0.845	5
IL-21 IL-12rb2 IL-2ra IL-18bp Tbet	0.764	0.925	0.845	5
CFU IL-21 LTA Gzmb Nos2 IL-2ra	0.767	0.923	0.845	6
CFU IL-21 Gzmb Nos2 IL-2ra IL-18bp	0.787	0.903	0.845	6
CFU IL-21 IL-2ra IL-18bp	0.779	0.91	0.844	4
CFU IL-21 Nos2 IL-12rb2 IL-18bp	0.769	0.919	0.844	5
CFU IL-21 Nos2 IL-2ra IL-18bp	0.782	0.907	0.844	5
CFU IL-21 IL-12rb2 IL-2ra IL-18bp	0.772	0.915	0.844	5
CFU LTA IL-12rb2 IL-2ra IL-18bp	0.757	0.931	0.844	5
CFU LTA Gzmb IL-12rb2 IL-2ra IL-18bp	0.766	0.922	0.844	6
IL-21 Nos2 IL-12rb2 IL-2ra IL-18bp Tbet	0.756	0.932	0.844	6
IFNg IL-21 Gzmb IL-12rb2 IL-2ra IL-18bp Tbet	0.732	0.956	0.844	7
CFU IL-21 LTA IL-18bp	0.751	0.935	0.843	4
CFU Nos2 IL-12rb2 IL-18bp	0.775	0.912	0.843	4
CFU LTA Gzmb IL-2ra IL-18bp	0.762	0.925	0.843	5
IL-21 Gzmb IL-12rb2 IL-18bp Tbet	0.768	0.918	0.843	5
CFU IL-21 Nos2 IL-12rb2 IL-2ra IL-18bp	0.775	0.912	0.843	6
CFU IL-21 LTA Gzmb Nos2 IL-12rb2 IL-2ra	0.766	0.92	0.843	7
CFU IL-21 LTA Gzmb Nos2 IL-12rb2	0.758	0.927	0.842	6
CFU LTA Nos2 IL-12rb2 IL-2ra IL-18bp	0.755	0.93	0.842	6
IFNg IL-21 IL-12rb2 IL-2ra IL-18bp Tbet	0.734	0.95	0.842	6
IL-21 Gzmb IL-12rb2 IL-2ra IL-18bp Tbet	0.758	0.926	0.842	6
IFNg IL-21 Nos2 IL-12rb2 IL-2ra IL-18bp Tbet	0.729	0.955	0.842	7
CFU IL-21 LTA Gzmb IL-18bp	0.751	0.931	0.841	5
CFU IL-21 LTA IL-12rb2 IL-18bp	0.748	0.934	0.841	5
IL-21 Nos2 Socs1 IL-2ra Tbet	0.747	0.935	0.841	5
CFU IL-21 LTA Nos2 IL-18bp	0.744	0.936	0.84	5
CFU LTA Gzmb Nos2 IL-18bp	0.747	0.933	0.84	5
CFU IL-21 LTA Gzmb IL-12rb2 IL-18bp	0.752	0.929	0.84	6
CFU LTA Gzmb Nos2 IL-12rb2 IL-18bp	0.749	0.931	0.84	6
IFNg IL-21 LTA Gzmb IL-12rb2 IL-2ra IL-18bp Tbet	0.726	0.955	0.84	8
IFNg Nos2 Socs1 IL-2ra	0.717	0.961	0.839	4
CFU IL-21 LTA IL-2ra IL-18bp	0.749	0.93	0.839	5
CFU IL-21 LTA Nos2 IL-12rb2 IL-18bp	0.743	0.935	0.839	6
IL-21 Gzmb Nos2 IL-12rb2 IL-18bp Tbet	0.757	0.921	0.839	6
IL-21 Nos2 Socs1 IL-2ra IL-18bp Tbet	0.742	0.937	0.839	6
IFNg IL-21 LTA IL-12rb2 IL-2ra IL-18bp Tbet	0.729	0.948	0.839	7
IFNg IL-21 Nos2 Socs1 IL-2ra	0.713	0.964	0.838	5

IFNg Gzmb IL-12rb2 IL-18bp Tbet	0.723	0.953	0.838	5
IFNg IL-21 LTA Nos2 Socs1 IL-2ra	0.717	0.958	0.838	6
CFU LTA Gzmb Nos2 IL-2ra IL-18bp	0.752	0.924	0.838	6
CFU IL-21 LTA IL-12rb2 IL-2ra IL-18bp	0.746	0.929	0.838	6
CFU LTA Gzmb Nos2 IL-12rb2 IL-2ra IL-18bp	0.754	0.921	0.838	7
IL-21 Gzmb Nos2 IL-12rb2 IL-2ra IL-18bp Tbet	0.746	0.93	0.838	7
IFNg IL-21 Gzmb Nos2 IL-12rb2 IL-2ra IL-18bp Tbet	0.72	0.957	0.838	8
IFNg Gzmb Nos2 Socs1 IL-2ra	0.708	0.966	0.837	5
CFU IL-21 LTA Gzmb IL-2ra IL-18bp	0.75	0.923	0.837	6
CFU IL-21 LTA Nos2 IL-2ra IL-18bp	0.745	0.93	0.837	6
IFNg IL-21 Gzmb IL-12rb2 IL-18bp Tbet	0.724	0.95	0.837	6
IFNg Gzmb IL-12rb2 IL-2ra IL-18bp Tbet	0.719	0.955	0.837	6
IFNg LTA Gzmb IL-12rb2 IL-2ra IL-18bp Tbet	0.717	0.957	0.837	7
IL-21 LTA IL-12rb2 IL-18bp Tbet	0.752	0.919	0.836	5
IFNg LTA Gzmb Nos2 Socs1 IL-2ra	0.714	0.958	0.836	6
CFU IL-21 LTA Gzmb IL-12rb2 IL-2ra IL-18bp	0.751	0.92	0.836	7
CFU IL-21 LTA Nos2 IL-12rb2 IL-2ra IL-18bp	0.743	0.928	0.836	7
IFNg LTA Nos2 Socs1 IL-2ra	0.715	0.955	0.835	5
CFU IL-21 LTA Gzmb Nos2 IL-18bp	0.738	0.932	0.835	6
IFNg IL-21 LTA Nos2 IL-12rb2 IL-2ra IL-18bp Tbet	0.717	0.953	0.835	8
IL-21 Gzmb Nos2 Socs1 IL-2ra Tbet	0.737	0.932	0.834	6
IFNg LTA Gzmb IL-12rb2 IL-18bp Tbet	0.715	0.953	0.834	6
IL-21 LTA Nos2 IL-12rb2 IL-18bp Tbet	0.739	0.929	0.834	6
IFNg Gzmb Nos2 IL-12rb2 IL-18bp Tbet	0.715	0.952	0.834	6
IL-21 Gzmb Nos2 Socs1 IL-2ra IL-18bp Tbet	0.733	0.934	0.834	7
IFNg IL-21 LTA Gzmb Nos2 IL-12rb2 IL-2ra IL-18bp Tbet	0.712	0.956	0.834	9
IFNg LTA Gzmb Socs1 IL-2ra	0.72	0.947	0.833	5
IFNg IL-21 Nos2 Socs1 IL-2ra Tbet	0.712	0.955	0.833	6
IFNg IL-21 Nos2 IL-12rb2 IL-18bp Tbet	0.72	0.946	0.833	6
CFU IL-21 LTA Gzmb Nos2 IL-12rb2 IL-18bp	0.739	0.928	0.833	7
IFNg Gzmb Nos2 IL-12rb2 IL-2ra IL-18bp Tbet	0.713	0.954	0.833	7
IL-21 Gzmb Socs1 IL-12rb2 IL-2ra IL-18bp Tbet	0.727	0.94	0.833	7
CFU	0.789	0.874	0.832	1
IFNg IL-21 IL-12rb2 IL-18bp Tbet	0.724	0.941	0.832	5
IFNg IL-21 Nos2 IL-12rb2 IL-2ra Tbet	0.712	0.952	0.832	6
IFNg IL-21 LTA Nos2 Socs1 IL-2ra Tbet	0.715	0.949	0.832	7
IFNg IL-21 LTA Gzmb IL-12rb2 IL-18bp Tbet	0.715	0.948	0.832	7
IL-21 LTA Nos2 Socs1 IL-2ra IL-18bp Tbet	0.724	0.939	0.832	7
CFU IL-18bp	0.779	0.884	0.831	2
IFNg IL-21 LTA Socs1 IL-2ra	0.724	0.938	0.831	5
IL-21 LTA Nos2 Socs1 IL-2ra Tbet	0.722	0.941	0.831	6
IL-21 Socs1 IL-12rb2 IL-2ra IL-18bp Tbet	0.728	0.934	0.831	6
CFU IL-21 LTA Gzmb Nos2 IL-2ra IL-18bp	0.739	0.922	0.831	7
IFNg IL-21 Gzmb Nos2 IL-12rb2 IL-18bp Tbet	0.712	0.95	0.831	7
IFNg LTA Gzmb Nos2 IL-12rb2 IL-2ra IL-18bp Tbet	0.704	0.957	0.831	8
IFNg Gzmb IL-12rb2 Tbet	0.715	0.945	0.83	4
IL-21 Socs1 IL-2ra IL-18bp Tbet	0.741	0.92	0.83	5
IL-21 Gzmb Socs1 IL-2ra IL-18bp Tbet	0.74	0.92	0.83	6
CFU Nos2 IL-18bp	0.776	0.882	0.829	3
IFNg Gzmb Socs1 IL-2ra	0.703	0.954	0.829	4
IL-21 Nos2 IL-12rb2 Tbet	0.748	0.91	0.829	4
IFNg Nos2 Socs1 IL-12rb2 IL-2ra	0.696	0.963	0.829	5
IL-21 Socs1 IL-12rb2 IL-18bp Tbet	0.741	0.916	0.829	5
IFNg IL-21 LTA IL-12rb2 IL-18bp Tbet	0.718	0.94	0.829	6
IL-21 Nos2 Socs1 IL-12rb2 IL-2ra IL-18bp Tbet	0.71	0.948	0.829	7
CFU IL-21 LTA Gzmb Nos2 IL-12rb2 IL-2ra IL-18bp	0.739	0.919	0.829	8
IFNg LTA Gzmb IL-12rb2 Tbet	0.71	0.945	0.828	5

IFNg Gzmb IL-12rb2 IL-2ra Tbet	0.711	0.945	0.828	5
IFNg IL-21 Gzmb IL-12rb2 IL-2ra Tbet	0.709	0.947	0.828	6
IFNg LTA Gzmb IL-12rb2 IL-2ra Tbet	0.709	0.947	0.828	6
IFNg IL-21 LTA Nos2 IL-12rb2 IL-2ra Tbet	0.703	0.952	0.828	7
IFNg LTA Gzmb Nos2 IL-12rb2 IL-18bp Tbet	0.704	0.952	0.828	7
IFNg LTA Gzmb Socs1	0.721	0.932	0.827	4
IFNg Gzmb Socs1 IL-12rb2	0.697	0.958	0.827	4
IFNg IL-21 Gzmb IL-12rb2 Tbet	0.71	0.944	0.827	5
IFNg Nos2 Socs1 IL-2ra Tbet	0.706	0.949	0.827	5
IL-21 LTA IL-12rb2 IL-2ra IL-18bp Tbet	0.724	0.93	0.827	6
IL-21 LTA Gzmb IL-12rb2 IL-2ra IL-18bp Tbet	0.718	0.935	0.827	7
CFU Nos2	0.775	0.878	0.826	2
IFNg IL-21 Nos2 Socs1 IL-12rb2 IL-2ra	0.687	0.965	0.826	6
IFNg Gzmb Nos2 Socs1 IL-2ra Tbet	0.698	0.954	0.826	6
IL-21 LTA Gzmb Nos2 Socs1 IL-2ra Tbet	0.714	0.937	0.826	7
IFNg IL-21 Nos2 Socs1 IL-2ra IL-18bp Tbet	0.697	0.955	0.826	7
IFNg IL-21 LTA Gzmb Nos2 IL-12rb2 IL-18bp Tbet	0.704	0.948	0.826	8
IL-21 LTA Gzmb Nos2 Socs1 IL-2ra IL-18bp Tbet	0.716	0.936	0.826	8
IFNg Nos2 Socs1 IL-2ra IL-18bp	0.691	0.959	0.825	5
IFNg IL-21 Nos2 IL-12rb2 Tbet	0.706	0.944	0.825	5
LTA Gzmb IL-12rb2 IL-18bp Tbet	0.724	0.926	0.825	5
IFNg IL-21 Nos2 Socs1 IL-2ra IL-18bp	0.688	0.961	0.825	6
IL-21 LTA Gzmb IL-12rb2 IL-18bp Tbet	0.726	0.925	0.825	6
IFNg IL-21 Gzmb Nos2 IL-12rb2 IL-2ra Tbet	0.698	0.952	0.825	7
IFNg IL-21 LTA Nos2 IL-12rb2 IL-18bp Tbet	0.706	0.943	0.825	7
IL-21 LTA Nos2 IL-12rb2 IL-2ra IL-18bp Tbet	0.708	0.942	0.825	7
IL-21 Gzmb Nos2 Socs1 IL-12rb2 IL-2ra IL-18bp Tbet	0.704	0.947	0.825	8
IFNg LTA Socs1	0.73	0.919	0.824	3
IFNg LTA Gzmb Socs1 IL-18bp	0.715	0.932	0.824	5
IFNg Gzmb Nos2 IL-12rb2 Tbet	0.699	0.949	0.824	5
IFNg Gzmb Socs1 IL-2ra Tbet	0.705	0.944	0.824	5
IFNg IL-21 IL-12rb2 IL-2ra Tbet	0.708	0.94	0.824	5
IL-21 Nos2 IL-12rb2 IL-2ra Tbet	0.732	0.916	0.824	5
IFNg IL-21 Gzmb Nos2 Socs1 IL-2ra	0.684	0.963	0.824	6
IFNg Gzmb Nos2 Socs1 IL-2ra IL-18bp	0.685	0.964	0.824	6
IFNg Gzmb Socs1 IL-12rb2 IL-18bp Tbet	0.696	0.952	0.824	6
IFNg IL-21 Gzmb Nos2 Socs1 IL-2ra Tbet	0.693	0.955	0.824	7
IFNg IL-21 LTA Gzmb IL-12rb2 IL-2ra Tbet	0.704	0.945	0.824	7
CFU Gzmb	0.755	0.891	0.823	2
CFU Gzmb IL-18bp	0.752	0.894	0.823	3
Nos2 Socs1 Tbet	0.753	0.893	0.823	3
IFNg Nos2 Socs1 IL-12rb2	0.695	0.951	0.823	4
IFNg Gzmb Socs1 IL-12rb2 IL-18bp	0.686	0.96	0.823	5
IL-21 Gzmb Socs1 IL-2ra Tbet	0.736	0.91	0.823	5
Gzmb Socs1 IL-12rb2 IL-18bp Tbet	0.73	0.917	0.823	5
Nos2 Socs1 IL-12rb2 IL-18bp Tbet	0.744	0.902	0.823	5
IL-21 Nos2 Socs1 IL-12rb2 IL-2ra Tbet	0.707	0.94	0.823	6
IL-21 LTA Socs1 IL-2ra IL-18bp Tbet	0.728	0.917	0.823	6
LTA Gzmb IL-12rb2 IL-2ra IL-18bp Tbet	0.717	0.93	0.823	6
Gzmb Socs1 IL-12rb2 IL-2ra IL-18bp Tbet	0.725	0.921	0.823	6
IFNg IL-21 LTA Socs1	0.722	0.922	0.822	4
CFU Gzmb Nos2 IL-18bp	0.754	0.891	0.822	4
IFNg LTA Gzmb Socs1 IL-12rb2	0.693	0.951	0.822	5
IFNg LTA Gzmb Socs1 Tbet	0.723	0.922	0.822	5
Gzmb Nos2 Socs1 IL-2ra Tbet	0.734	0.91	0.822	5
IFNg LTA Gzmb Socs1 IL-2ra IL-18bp	0.696	0.949	0.822	6
IFNg Nos2 Socs1 IL-12rb2 IL-2ra IL-18bp	0.679	0.964	0.822	6

IFNg IL-21 LTA Gzmb IL-12rb2 Tbet	0.703	0.941	0.822	6
IFNg Gzmb Nos2 IL-12rb2 IL-2ra Tbet	0.695	0.949	0.822	6
LTA Nos2 Socs1 IL-12rb2 IL-18bp Tbet	0.719	0.926	0.822	6
IFNg IL-21 LTA Gzmb Nos2 Socs1 IL-2ra	0.686	0.957	0.822	7
IFNg LTA Gzmb Nos2 IL-12rb2 IL-2ra Tbet	0.69	0.954	0.822	7
IFNg IL-21 LTA Gzmb Nos2 IL-12rb2 IL-2ra Tbet	0.692	0.952	0.822	8
IL-21 Socs1 IL-2ra Tbet	0.736	0.907	0.821	4
Nos2 Socs1 IL-2ra Tbet	0.75	0.891	0.821	4
IFNg Gzmb Socs1 IL-2ra IL-18bp	0.686	0.956	0.821	5
IL-21 Gzmb Nos2 IL-12rb2 Tbet	0.736	0.905	0.821	5
Gzmb Socs1 IL-2ra IL-18bp Tbet	0.741	0.902	0.821	5
IFNg LTA Nos2 Socs1 IL-12rb2 IL-2ra	0.683	0.96	0.821	6
IFNg LTA Gzmb Socs1 IL-2ra Tbet	0.703	0.938	0.821	6
IFNg IL-21 LTA IL-12rb2 IL-2ra Tbet	0.705	0.938	0.821	6
IL-21 LTA Socs1 IL-12rb2 IL-18bp Tbet	0.726	0.916	0.821	6
IL-21 Gzmb Socs1 IL-12rb2 IL-18bp Tbet	0.721	0.922	0.821	6
IL-21 Nos2 Socs1 IL-12rb2 IL-18bp Tbet	0.716	0.926	0.821	6
IFNg Gzmb Socs1 IL-2ra IL-18bp Tbet	0.697	0.945	0.821	6
IFNg Nos2 Socs1 IL-2ra IL-18bp Tbet	0.693	0.948	0.821	6
IFNg LTA Gzmb Nos2 Socs1 IL-2ra IL-18bp	0.684	0.958	0.821	7
IFNg LTA Gzmb Nos2 Socs1 IL-2ra Tbet	0.692	0.949	0.821	7
IFNg Gzmb Nos2 Socs1 IL-2ra IL-18bp Tbet	0.689	0.954	0.821	7
IFNg IL-21 LTA Nos2 Socs1 IL-2ra IL-18bp Tbet	0.693	0.948	0.821	8
IFNg Socs1	0.709	0.93	0.82	2
CFU Gzmb Nos2	0.75	0.89	0.82	3
IFNg LTA Socs1 IL-12rb2	0.711	0.929	0.82	4
IFNg IL-21 Socs1 IL-2ra	0.696	0.943	0.82	4
LTA Nos2 Socs1 IL-2ra	0.701	0.939	0.82	4
IFNg LTA Socs1 IL-18bp	0.719	0.921	0.82	4
IFNg IL-21 IL-12rb2 Tbet	0.706	0.934	0.82	4
IFNg Gzmb Nos2 Socs1 IL-12rb2	0.679	0.96	0.82	5
IFNg LTA Socs1 IL-12rb2 IL-18bp	0.705	0.935	0.82	5
IFNg Gzmb Socs1 IL-12rb2 Tbet	0.694	0.947	0.82	5
Nos2 Socs1 IL-2ra IL-18bp Tbet	0.741	0.899	0.82	5
IFNg IL-21 LTA Socs1 IL-2ra IL-18bp	0.698	0.941	0.82	6
IFNg IL-21 Gzmb Nos2 IL-12rb2 Tbet	0.695	0.946	0.82	6
IFNg LTA Gzmb Nos2 IL-12rb2 Tbet	0.691	0.95	0.82	6
Gzmb Nos2 Socs1 IL-2ra IL-18bp Tbet	0.727	0.913	0.82	6
IFNg IL-21 LTA Gzmb Nos2 Socs1 IL-2ra Tbet	0.69	0.949	0.82	8
IL-21 LTA Gzmb Nos2 IL-12rb2 IL-2ra IL-18bp Tbet	0.698	0.941	0.82	8
IFNg Socs1 IL-12rb2	0.7	0.938	0.819	3
IFNg LTA Nos2 Socs1	0.702	0.936	0.819	4
IFNg Nos2 Socs1 Tbet	0.703	0.936	0.819	4
Gzmb Nos2 Socs1 Tbet	0.734	0.904	0.819	4
Nos2 Socs1 IL-18bp Tbet	0.74	0.898	0.819	4
IFNg IL-21 LTA Gzmb Socs1	0.709	0.929	0.819	5
IFNg IL-21 Gzmb Socs1 IL-2ra	0.688	0.95	0.819	5
IFNg Gzmb Socs1 IL-12rb2 IL-2ra	0.673	0.965	0.819	5
IFNg Nos2 IL-12rb2 IL-18bp Tbet	0.696	0.942	0.819	5
IFNg IL-21 LTA Nos2 IL-12rb2 Tbet	0.696	0.943	0.819	6
IFNg IL-21 Gzmb Socs1 IL-2ra Tbet	0.694	0.943	0.819	6
IFNg LTA Nos2 Socs1 IL-2ra Tbet	0.695	0.944	0.819	6
LTA Gzmb Nos2 IL-12rb2 IL-18bp Tbet	0.708	0.93	0.819	6
IFNg IL-21 LTA Nos2 Socs1 IL-2ra IL-18bp	0.682	0.957	0.819	7
IFNg IL-21 Nos2 Socs1 IL-12rb2 IL-2ra IL-18bp	0.672	0.966	0.819	7
IL-21 LTA Gzmb Nos2 IL-12rb2 IL-18bp Tbet	0.71	0.929	0.819	7
Gzmb Socs1 Tbet	0.75	0.886	0.818	3

Socs1 IL-12rb2 IL-18bp Tbet	0.761	0.874	0.818	4
IFNg IL-21 Gzmb Socs1 IL-12rb2	0.68	0.957	0.818	5
IFNg IL-21 Nos2 Socs1 IL-12rb2	0.687	0.949	0.818	5
IFNg LTA Nos2 Socs1 IL-12rb2	0.692	0.944	0.818	5
IFNg Nos2 Socs1 IL-12rb2 IL-18bp	0.686	0.951	0.818	5
LTA Nos2 Socs1 IL-2ra Tbet	0.708	0.928	0.818	5
IFNg IL-21 LTA Gzmb Socs1 IL-2ra	0.692	0.945	0.818	6
IFNg Gzmb Socs1 IL-12rb2 IL-2ra Tbet	0.68	0.956	0.818	6
IFNg LTA Gzmb Socs1 IL-18bp Tbet	0.714	0.921	0.818	6
IL-21 LTA Nos2 Socs1 IL-12rb2 IL-18bp Tbet	0.708	0.929	0.818	7
IFNg IL-21 Gzmb Nos2 Socs1 IL-2ra IL-18bp Tbet	0.683	0.954	0.818	8
IFNg Socs1 IL-2ra	0.698	0.936	0.817	3
LTA Nos2 Socs1 Tbet	0.722	0.912	0.817	4
IFNg LTA Gzmb Nos2 Socs1	0.695	0.939	0.817	5
IFNg LTA Nos2 Socs1 IL-18bp	0.7	0.934	0.817	5
IFNg LTA Nos2 Socs1 Tbet	0.707	0.927	0.817	5
IFNg IL-21 LTA IL-12rb2 Tbet	0.702	0.932	0.817	5
IFNg IL-21 Gzmb Socs1 IL-12rb2 IL-2ra	0.67	0.963	0.817	6
IFNg LTA Gzmb Socs1 IL-12rb2 IL-18bp	0.679	0.956	0.817	6
IFNg Gzmb Nos2 Socs1 IL-12rb2 IL-18bp	0.673	0.96	0.817	6
IFNg LTA Nos2 Socs1 IL-2ra IL-18bp	0.68	0.954	0.817	6
IFNg IL-21 LTA Socs1 IL-2ra Tbet	0.705	0.929	0.817	6
IL-21 Gzmb Nos2 IL-12rb2 IL-2ra Tbet	0.723	0.911	0.817	6
IFNg LTA Nos2 IL-12rb2 IL-18bp Tbet	0.691	0.944	0.817	6
LTA Gzmb Socs1 IL-12rb2 IL-18bp Tbet	0.707	0.926	0.817	6
Gzmb Nos2 Socs1 IL-12rb2 IL-18bp Tbet	0.714	0.919	0.817	6
IFNg IL-21 Nos2 Socs1 IL-12rb2 IL-18bp Tbet	0.691	0.944	0.817	7
IFNg IL-21 LTA Socs1 IL-2ra IL-18bp Tbet	0.701	0.933	0.817	7
IFNg LTA Gzmb Socs1 IL-2ra IL-18bp Tbet	0.695	0.939	0.817	7
IFNg LTA Nos2 IL-12rb2 IL-2ra IL-18bp Tbet	0.689	0.946	0.817	7
LTA Gzmb Nos2 IL-12rb2 IL-2ra IL-18bp Tbet	0.698	0.935	0.817	7
IFNg Gzmb Socs1 IL-12rb2 IL-2ra IL-18bp Tbet	0.664	0.969	0.817	7
IFNg Nos2 Socs1 IL-12rb2 IL-2ra IL-18bp Tbet	0.679	0.955	0.817	7
IL-21 IL-12rb2 Tbet	0.738	0.893	0.816	3
IFNg LTA Socs1 IL-2ra	0.706	0.927	0.816	4
IL-21 Nos2 Socs1 Tbet	0.719	0.913	0.816	4
IFNg Nos2 IL-12rb2 Tbet	0.693	0.939	0.816	4
Gzmb Socs1 IL-18bp Tbet	0.736	0.895	0.816	4
IFNg IL-12rb2 IL-18bp Tbet	0.7	0.932	0.816	4
IFNg IL-21 LTA Socs1 IL-18bp	0.71	0.923	0.816	5
IFNg LTA Nos2 IL-12rb2 Tbet	0.69	0.943	0.816	5
LTA Nos2 IL-12rb2 IL-18bp Tbet	0.708	0.923	0.816	5
IFNg Gzmb Socs1 IL-12rb2 IL-2ra IL-18bp	0.659	0.973	0.816	6
LTA Gzmb Nos2 Socs1 IL-2ra Tbet	0.698	0.935	0.816	6
IFNg LTA Nos2 IL-12rb2 IL-2ra Tbet	0.687	0.945	0.816	6
IL-21 LTA Nos2 IL-12rb2 IL-2ra Tbet	0.693	0.939	0.816	6
IFNg Nos2 Socs1 IL-12rb2 IL-2ra Tbet	0.679	0.953	0.816	6
IFNg Nos2 Socs1 IL-12rb2 IL-18bp Tbet	0.689	0.943	0.816	6
IFNg IL-21 Gzmb Nos2 Socs1 IL-2ra IL-18bp	0.67	0.962	0.816	7
IFNg IL-21 LTA Gzmb Nos2 IL-12rb2 Tbet	0.687	0.945	0.816	7
IFNg IL-21 LTA Gzmb Socs1 IL-2ra Tbet	0.694	0.937	0.816	7
Gzmb Nos2 Socs1 IL-12rb2 IL-2ra IL-18bp Tbet	0.708	0.924	0.816	7
IFNg Gzmb Socs1	0.689	0.941	0.815	3
IFNg Nos2 Socs1	0.683	0.947	0.815	3
IFNg Socs1 IL-12rb2 IL-2ra	0.688	0.941	0.815	4
IFNg Socs1 IL-12rb2 IL-18bp	0.687	0.942	0.815	4
LTA Gzmb IL-12rb2 Tbet	0.716	0.915	0.815	4

IL-21 LTA Nos2 Socs1 IL-2ra	0.683	0.946	0.815	5
IFNg IL-21 Socs1 IL-12rb2 IL-2ra	0.682	0.949	0.815	5
LTA Nos2 Socs1 IL-2ra IL-18bp	0.692	0.938	0.815	5
IFNg Socs1 IL-12rb2 IL-2ra IL-18bp	0.682	0.948	0.815	5
IL-21 LTA Nos2 IL-12rb2 Tbet	0.704	0.926	0.815	5
Gzmb Nos2 Socs1 IL-18bp Tbet	0.723	0.907	0.815	5
IFNg IL-21 LTA Socs1 IL-12rb2 IL-2ra	0.685	0.944	0.815	6
IFNg LTA Gzmb Nos2 Socs1 IL-18bp	0.692	0.937	0.815	6
IFNg LTA Nos2 Socs1 IL-12rb2 IL-18bp	0.686	0.945	0.815	6
IFNg IL-21 Socs1 IL-12rb2 IL-2ra IL-18bp	0.676	0.953	0.815	6
IFNg LTA Gzmb Nos2 Socs1 Tbet	0.7	0.929	0.815	6
IFNg Nos2 IL-12rb2 IL-2ra IL-18bp Tbet	0.69	0.94	0.815	6
IFNg IL-21 LTA Socs1 IL-12rb2 IL-2ra IL-18bp	0.679	0.951	0.815	7
IL-21 Gzmb Nos2 Socs1 IL-12rb2 IL-2ra Tbet	0.694	0.937	0.815	7
IFNg Gzmb Nos2 Socs1 IL-12rb2 IL-18bp Tbet	0.678	0.952	0.815	7
IL-21 Gzmb Nos2 Socs1 IL-12rb2 IL-18bp Tbet	0.704	0.926	0.815	7
IFNg IL-21 Gzmb Socs1 IL-2ra IL-18bp Tbet	0.686	0.944	0.815	7
IFNg IL-21 Socs1 IL-12rb2 IL-2ra IL-18bp Tbet	0.678	0.952	0.815	7
IL-21 LTA Socs1 IL-12rb2 IL-2ra IL-18bp Tbet	0.694	0.935	0.815	7
IL-21 LTA Nos2 Socs1 IL-12rb2 IL-2ra IL-18bp Tbet	0.677	0.953	0.815	8
IFNg Nos2 Socs1 IL-18bp	0.685	0.942	0.814	4
IL-21 Nos2 IL-2ra Tbet	0.714	0.913	0.814	4
Gzmb Socs1 IL-2ra Tbet	0.739	0.889	0.814	4
IFNg IL-21 LTA Nos2 Socs1	0.693	0.934	0.814	5
IFNg IL-21 LTA Socs1 IL-12rb2	0.694	0.933	0.814	5
LTA Gzmb Nos2 Socs1 IL-2ra	0.678	0.949	0.814	5
IFNg IL-21 Socs1 IL-2ra IL-18bp	0.684	0.944	0.814	5
IFNg IL-21 LTA Socs1 Tbet	0.715	0.912	0.814	5
IFNg Gzmb Nos2 Socs1 Tbet	0.692	0.936	0.814	5
LTA Nos2 Socs1 IL-18bp Tbet	0.718	0.91	0.814	5
IFNg IL-21 LTA Socs1 IL-12rb2 IL-18bp	0.69	0.937	0.814	6
IFNg IL-21 Gzmb Socs1 IL-2ra IL-18bp	0.676	0.951	0.814	6
IFNg IL-21 Gzmb Socs1 IL-12rb2 Tbet	0.681	0.947	0.814	6
IFNg IL-21 LTA Nos2 Socs1 IL-12rb2 IL-2ra	0.668	0.961	0.814	7
LTA Nos2 Socs1 IL-12rb2 IL-2ra IL-18bp Tbet	0.689	0.938	0.814	7
IFNg LTA Gzmb Nos2 Socs1 IL-2ra IL-18bp Tbet	0.679	0.948	0.814	8
IFNg Gzmb IL-12rb2	0.675	0.952	0.813	3
IFNg IL-21 Nos2 Socs1	0.683	0.943	0.813	4
IFNg Socs1 IL-2ra IL-18bp	0.688	0.938	0.813	4
LTA Nos2 IL-12rb2 Tbet	0.704	0.921	0.813	4
LTA Gzmb Nos2 Socs1 Tbet	0.711	0.914	0.813	5
IL-21 LTA Gzmb IL-12rb2 Tbet	0.713	0.913	0.813	5
LTA Gzmb IL-12rb2 IL-2ra Tbet	0.71	0.916	0.813	5
IL-21 Nos2 Socs1 IL-18bp Tbet	0.711	0.914	0.813	5
IFNg LTA IL-12rb2 IL-18bp Tbet	0.694	0.933	0.813	5
IL-21 Nos2 IL-2ra IL-18bp Tbet	0.709	0.917	0.813	5
IFNg IL-21 LTA Nos2 Socs1 IL-12rb2	0.683	0.943	0.813	6
IFNg LTA Gzmb Socs1 IL-12rb2 IL-2ra	0.666	0.96	0.813	6
IFNg LTA Nos2 Socs1 IL-18bp Tbet	0.701	0.926	0.813	6
LTA Nos2 IL-12rb2 IL-2ra IL-18bp Tbet	0.702	0.925	0.813	6
Nos2 Socs1 IL-12rb2 IL-2ra IL-18bp Tbet	0.726	0.9	0.813	6
IFNg IL-21 Gzmb Socs1 IL-12rb2 IL-2ra IL-18bp	0.651	0.974	0.813	7
IFNg LTA Gzmb Socs1 IL-12rb2 IL-2ra IL-18bp	0.658	0.969	0.813	7
IL-21 LTA Gzmb Socs1 IL-2ra IL-18bp Tbet	0.708	0.918	0.813	7
IFNg IL-21 LTA Gzmb Socs1 IL-2ra IL-18bp Tbet	0.687	0.939	0.813	8
IFNg IL-21 Nos2 Socs1 IL-12rb2 IL-2ra IL-18bp Tbet	0.663	0.964	0.813	8
IFNg LTA Socs1 Tbet	0.715	0.909	0.812	4

IL-21 IL-2ra IL-18bp Tbet	0.717	0.908	0.812	4
LTA Nos2 Socs1 IL-12rb2 IL-2ra	0.684	0.941	0.812	5
IFNg IL-21 Nos2 Socs1 Tbet	0.69	0.934	0.812	5
LTA Gzmb Nos2 IL-12rb2 Tbet	0.694	0.93	0.812	5
IFNg Nos2 Socs1 IL-12rb2 Tbet	0.682	0.942	0.812	5
IFNg IL-21 Socs1 IL-2ra Tbet	0.691	0.934	0.812	5
IFNg Nos2 IL-12rb2 IL-2ra Tbet	0.687	0.937	0.812	5
IFNg Nos2 Socs1 IL-18bp Tbet	0.69	0.934	0.812	5
IFNg IL-21 LTA Gzmb Nos2 Socs1	0.687	0.936	0.812	6
IFNg IL-21 Gzmb Socs1 IL-12rb2 IL-18bp	0.661	0.963	0.812	6
IL-21 LTA Nos2 Socs1 IL-2ra IL-18bp	0.678	0.945	0.812	6
IFNg LTA Socs1 IL-12rb2 IL-2ra IL-18bp	0.682	0.942	0.812	6
IFNg IL-21 LTA Gzmb Socs1 Tbet	0.705	0.92	0.812	6
IFNg IL-21 LTA Nos2 Socs1 Tbet	0.698	0.926	0.812	6
IFNg IL-21 Socs1 IL-12rb2 IL-18bp Tbet	0.688	0.936	0.812	6
LTA Nos2 Socs1 IL-2ra IL-18bp Tbet	0.699	0.926	0.812	6
IFNg IL-21 LTA Gzmb Socs1 IL-2ra IL-18bp	0.677	0.947	0.812	7
IFNg LTA Nos2 Socs1 IL-12rb2 IL-2ra IL-18bp	0.664	0.96	0.812	7
IFNg IL-21 Gzmb Socs1 IL-12rb2 IL-2ra Tbet	0.668	0.955	0.812	7
IFNg LTA Gzmb Nos2 Socs1 IL-18bp Tbet	0.696	0.927	0.812	7
LTA Gzmb Nos2 Socs1 IL-12rb2 IL-18bp Tbet	0.693	0.931	0.812	7
IFNg LTA Nos2 Socs1 IL-2ra IL-18bp Tbet	0.68	0.943	0.812	7
IFNg IL-21 Gzmb Socs1 IL-12rb2 IL-2ra IL-18bp Tbet	0.656	0.968	0.812	8
IL-21 Nos2 Tbet	0.719	0.904	0.811	3
IFNg Gzmb Socs1 Tbet	0.695	0.927	0.811	4
IL-21 Gzmb IL-12rb2 Tbet	0.727	0.894	0.811	4
Nos2 Socs1 IL-12rb2 Tbet	0.731	0.891	0.811	4
IFNg LTA Socs1 IL-12rb2 IL-2ra	0.689	0.933	0.811	5
IL-21 LTA Nos2 Socs1 Tbet	0.709	0.914	0.811	5
IL-21 Nos2 Socs1 IL-12rb2 Tbet	0.703	0.919	0.811	5
LTA Nos2 Socs1 IL-12rb2 Tbet	0.696	0.926	0.811	5
IFNg IL-12rb2 IL-2ra IL-18bp Tbet	0.692	0.93	0.811	5
IFNg LTA Gzmb Nos2 Socs1 IL-12rb2	0.668	0.953	0.811	6
IFNg Gzmb Nos2 Socs1 IL-12rb2 IL-2ra	0.649	0.973	0.811	6
IFNg IL-21 Nos2 Socs1 IL-12rb2 IL-18bp	0.673	0.948	0.811	6
IL-21 LTA Gzmb IL-12rb2 IL-2ra Tbet	0.703	0.919	0.811	6
IFNg IL-21 Socs1 IL-2ra IL-18bp Tbet	0.685	0.938	0.811	6
IFNg LTA IL-12rb2 IL-2ra IL-18bp Tbet	0.69	0.932	0.811	6
IFNg Gzmb Nos2 Socs1 IL-12rb2 IL-2ra IL-18bp	0.645	0.976	0.811	7
IFNg LTA Gzmb Socs1 IL-12rb2 IL-18bp Tbet	0.673	0.949	0.811	7
IL-21 LTA Gzmb Socs1 IL-12rb2 IL-2ra IL-18bp Tbet	0.677	0.945	0.811	8
IFNg IL-21 LTA Gzmb Nos2 Socs1 IL-2ra IL-18bp Tbet	0.674	0.948	0.811	9
IFNg IL-21 Socs1	0.694	0.926	0.81	3
IFNg IL-21 Gzmb Socs1	0.686	0.934	0.81	4
IFNg IL-21 Socs1 IL-12rb2	0.682	0.938	0.81	4
IL-21 Gzmb Socs1 Tbet	0.728	0.893	0.81	4
LTA Gzmb Socs1 IL-2ra Tbet	0.708	0.912	0.81	5
LTA Nos2 IL-12rb2 IL-2ra Tbet	0.698	0.923	0.81	5
IFNg IL-21 LTA Nos2 Socs1 IL-18bp	0.688	0.933	0.81	6
LTA Gzmb Nos2 IL-12rb2 IL-2ra Tbet	0.686	0.934	0.81	6
LTA Nos2 Socs1 IL-12rb2 IL-2ra Tbet	0.682	0.939	0.81	6
IFNg IL-21 LTA Socs1 IL-18bp Tbet	0.707	0.914	0.81	6
IFNg IL-21 Gzmb Socs1 IL-12rb2 IL-18bp Tbet	0.667	0.953	0.81	7
LTA Gzmb Nos2 Socs1 IL-2ra IL-18bp Tbet	0.69	0.931	0.81	7
IFNg Gzmb Nos2 Socs1	0.669	0.949	0.809	4
IL-21 LTA IL-12rb2 Tbet	0.714	0.904	0.809	4
IL-21 Nos2 IL-18bp Tbet	0.711	0.906	0.809	4

IFNg IL-21 Gzmb Nos2 Socs1	0.674	0.944	0.809	5
IFNg LTA Socs1 IL-2ra IL-18bp	0.687	0.931	0.809	5
Gzmb Nos2 Socs1 IL-12rb2 Tbet	0.707	0.912	0.809	5
IFNg IL-21 Nos2 IL-2ra Tbet	0.675	0.942	0.809	5
IL-21 LTA IL-12rb2 IL-2ra Tbet	0.706	0.913	0.809	5
Gzmb IL-12rb2 IL-2ra IL-18bp Tbet	0.734	0.885	0.809	5
Socs1 IL-12rb2 IL-2ra IL-18bp Tbet	0.746	0.873	0.809	5
IL-21 LTA Gzmb Nos2 Socs1 IL-2ra	0.669	0.949	0.809	6
IL-21 LTA Gzmb Nos2 IL-12rb2 Tbet	0.694	0.924	0.809	6
IFNg IL-21 Nos2 Socs1 IL-12rb2 Tbet	0.676	0.942	0.809	6
IL-21 LTA Gzmb Socs1 IL-2ra Tbet	0.701	0.916	0.809	6
IFNg IL-21 Socs1 IL-12rb2 IL-2ra Tbet	0.677	0.942	0.809	6
IFNg LTA Gzmb Nos2 Socs1 IL-12rb2 IL-18bp	0.661	0.956	0.809	7
IL-21 LTA Gzmb Nos2 IL-12rb2 IL-2ra Tbet	0.684	0.934	0.809	7
IFNg LTA Gzmb Socs1 IL-12rb2 IL-2ra Tbet	0.667	0.952	0.809	7
IFNg LTA Nos2 Socs1 IL-12rb2 IL-2ra Tbet	0.666	0.953	0.809	7
IFNg IL-21 LTA Gzmb Nos2 Socs1 IL-2ra IL-18bp	0.661	0.956	0.809	8
IFNg LTA Nos2 Socs1 IL-12rb2 IL-2ra IL-18bp Tbet	0.665	0.953	0.809	8
IFNg Gzmb Nos2 Socs1 IL-12rb2 IL-2ra IL-18bp Tbet	0.647	0.971	0.809	8
IL-21 Socs1 Tbet	0.727	0.89	0.808	3
IFNg IL-12rb2 Tbet	0.694	0.922	0.808	3
IFNg LTA Gzmb IL-12rb2	0.666	0.951	0.808	4
IL-21 Nos2 Socs1 IL-2ra	0.681	0.936	0.808	4
IFNg Gzmb Nos2 Socs1 IL-18bp	0.672	0.944	0.808	5
IL-21 Gzmb Nos2 Socs1 Tbet	0.706	0.911	0.808	5
IFNg Gzmb Nos2 Socs1 IL-12rb2 Tbet	0.665	0.951	0.808	6
IL-21 LTA Nos2 Socs1 IL-18bp Tbet	0.704	0.912	0.808	6
LTA Gzmb Nos2 Socs1 IL-18bp Tbet	0.705	0.911	0.808	6
IFNg IL-21 LTA Nos2 Socs1 IL-18bp Tbet	0.692	0.925	0.808	7
IFNg IL-21 LTA Socs1 IL-12rb2 IL-18bp Tbet	0.684	0.933	0.808	7
IL-21 LTA Gzmb Socs1 IL-12rb2 IL-18bp Tbet	0.691	0.925	0.808	7
IFNg LTA Gzmb Socs1 IL-12rb2 IL-2ra IL-18bp Tbet	0.651	0.965	0.808	8
IFNg IL-12rb2	0.679	0.934	0.807	2
IFNg IL-21 IL-12rb2	0.676	0.938	0.807	3
IFNg Nos2 IL-12rb2	0.67	0.943	0.807	3
IFNg Nos2 Tbet	0.686	0.929	0.807	3
IFNg Socs1 Tbet	0.698	0.916	0.807	3
IFNg IL-21 Nos2 Tbet	0.683	0.931	0.807	4
LTA Gzmb Socs1 Tbet	0.718	0.896	0.807	4
IFNg Socs1 IL-12rb2 Tbet	0.69	0.924	0.807	4
IL-21 Socs1 IL-12rb2 Tbet	0.718	0.897	0.807	4
IFNg Socs1 IL-2ra Tbet	0.691	0.923	0.807	4
IL-21 IL-12rb2 IL-2ra Tbet	0.718	0.897	0.807	4
IFNg IL-21 Socs1 IL-12rb2 IL-18bp	0.673	0.941	0.807	5
IL-21 Gzmb Nos2 IL-2ra Tbet	0.705	0.909	0.807	5
LTA Gzmb Socs1 IL-18bp Tbet	0.721	0.894	0.807	5
IFNg Socs1 IL-12rb2 IL-18bp Tbet	0.682	0.931	0.807	5
IFNg IL-21 LTA Gzmb Socs1 IL-12rb2	0.662	0.951	0.807	6
IFNg IL-21 Gzmb Nos2 Socs1 IL-12rb2	0.653	0.961	0.807	6
LTA Gzmb Nos2 Socs1 IL-2ra IL-18bp	0.667	0.948	0.807	6
LTA Nos2 Socs1 IL-12rb2 IL-2ra IL-18bp	0.675	0.939	0.807	6
IFNg Gzmb Nos2 Socs1 IL-18bp Tbet	0.679	0.935	0.807	6
IFNg IL-21 LTA Gzmb Socs1 IL-12rb2 IL-2ra	0.656	0.959	0.807	7
IFNg IL-21 LTA Nos2 Socs1 IL-12rb2 IL-18bp	0.672	0.943	0.807	7
IFNg Gzmb Nos2 Socs1 IL-12rb2 IL-2ra Tbet	0.648	0.966	0.807	7
LTA Gzmb Socs1 IL-12rb2 IL-2ra IL-18bp Tbet	0.673	0.941	0.807	7
IL-21 IL-18bp Tbet	0.716	0.895	0.806	3

IFNg LTA IL-12rb2 Tbet	0.689	0.924	0.806	4
IFNg IL-21 LTA Nos2 Tbet	0.686	0.925	0.806	5
IFNg LTA Socs1 IL-18bp Tbet	0.701	0.911	0.806	5
IL-21 Gzmb IL-2ra IL-18bp Tbet	0.707	0.906	0.806	5
IFNg Socs1 IL-2ra IL-18bp Tbet	0.685	0.926	0.806	5
IL-21 Gzmb Nos2 IL-2ra IL-18bp Tbet	0.698	0.915	0.806	6
Gzmb Nos2 IL-12rb2 IL-2ra IL-18bp Tbet	0.73	0.883	0.806	6
IFNg Socs1 IL-12rb2 IL-2ra IL-18bp Tbet	0.677	0.936	0.806	6
IFNg IL-21 Gzmb Nos2 Socs1 IL-12rb2 IL-2ra	0.64	0.971	0.806	7
IFNg IL-21 Gzmb Nos2 Socs1 IL-12rb2 IL-18bp	0.648	0.964	0.806	7
IFNg IL-21 LTA Gzmb Nos2 Socs1 Tbet	0.685	0.926	0.806	7
IFNg IL-21 Nos2 Socs1 IL-12rb2 IL-2ra Tbet	0.65	0.961	0.806	7
IFNg IL-21 Gzmb Nos2 Socs1 IL-12rb2 IL-2ra IL-18bp	0.636	0.976	0.806	8
IFNg IL-21 LTA Gzmb Socs1 IL-12rb2 IL-2ra IL-18bp Tbet	0.649	0.963	0.806	9
Nos2 Socs1	0.713	0.898	0.805	2
IL-21 Tbet	0.723	0.886	0.805	2
IFNg Socs1 IL-18bp	0.682	0.929	0.805	3
IFNg IL-21 Nos2 IL-12rb2	0.666	0.944	0.805	4
IFNg Gzmb IL-12rb2 IL-2ra	0.659	0.952	0.805	4
IL-21 Socs1 IL-18bp Tbet	0.713	0.898	0.805	4
LTA IL-12rb2 IL-18bp Tbet	0.711	0.899	0.805	4
Gzmb IL-12rb2 IL-18bp Tbet	0.725	0.885	0.805	4
IFNg IL-21 Nos2 Socs1 IL-18bp	0.671	0.939	0.805	5
IL-21 Nos2 Socs1 IL-2ra IL-18bp	0.674	0.937	0.805	5
IFNg IL-21 Socs1 IL-12rb2 Tbet	0.68	0.931	0.805	5
IL-21 LTA Socs1 IL-2ra Tbet	0.7	0.91	0.805	5
IL-21 Gzmb Socs1 IL-18bp Tbet	0.713	0.898	0.805	5
IL-21 LTA Gzmb Nos2 Socs1 Tbet	0.7	0.911	0.805	6
IFNg LTA Gzmb Socs1 IL-12rb2 Tbet	0.668	0.942	0.805	6
Gzmb Nos2 Socs1 IL-12rb2 IL-2ra Tbet	0.697	0.913	0.805	6
IL-21 Gzmb Nos2 Socs1 IL-18bp Tbet	0.699	0.911	0.805	6
IFNg LTA Nos2 Socs1 IL-12rb2 IL-18bp Tbet	0.671	0.939	0.805	7
IFNg IL-21 LTA Gzmb Socs1 IL-12rb2 IL-2ra IL-18bp	0.64	0.97	0.805	8
IFNg LTA Gzmb Nos2 Socs1 IL-12rb2 IL-2ra IL-18bp	0.638	0.972	0.805	8
IL-21 LTA Gzmb Nos2 Socs1 IL-12rb2 IL-18bp Tbet	0.68	0.93	0.805	8
IFNg IL-21 Gzmb Nos2 Socs1 IL-12rb2 IL-2ra IL-18bp Tbet	0.64	0.97	0.805	9
IFNg	0.681	0.926	0.804	1
Socs1 IL-18bp Tbet	0.738	0.869	0.804	3
IFNg IL-21 Gzmb IL-12rb2	0.656	0.952	0.804	4
IFNg Gzmb Nos2 IL-12rb2	0.655	0.952	0.804	4
IFNg Gzmb Socs1 IL-18bp	0.67	0.938	0.804	4
IFNg LTA Nos2 Tbet	0.682	0.926	0.804	4
IL-21 LTA Nos2 Tbet	0.699	0.91	0.804	4
IFNg Socs1 IL-12rb2 IL-2ra Tbet	0.682	0.926	0.804	5
IFNg IL-21 Gzmb Nos2 Socs1 Tbet	0.672	0.935	0.804	6
IL-21 Gzmb Nos2 Socs1 IL-12rb2 Tbet	0.69	0.918	0.804	6
IL-21 Gzmb Socs1 IL-12rb2 IL-2ra Tbet	0.694	0.915	0.804	6
LTA Gzmb Socs1 IL-12rb2 IL-2ra Tbet	0.676	0.932	0.804	6
LTA Gzmb Socs1 IL-2ra IL-18bp Tbet	0.697	0.911	0.804	6
IFNg LTA Gzmb Nos2 Socs1 IL-12rb2 IL-2ra	0.64	0.968	0.804	7
IFNg IL-21 LTA Nos2 Socs1 IL-12rb2 IL-2ra IL-18bp	0.646	0.962	0.804	8
IFNg IL-21 LTA Nos2 Socs1 IL-12rb2 IL-18bp Tbet	0.671	0.938	0.804	8
IL-21 LTA Gzmb Nos2 Socs1 IL-12rb2 IL-2ra IL-18bp Tbet	0.654	0.954	0.804	9
IFNg LTA	0.682	0.924	0.803	2
IL-21 IL-2ra Tbet	0.711	0.894	0.803	3
LTA Nos2 Socs1 IL-12rb2	0.685	0.921	0.803	4
IFNg Gzmb IL-12rb2 IL-18bp	0.654	0.952	0.803	4

IFNg IL-12rb2 IL-2ra Tbet	0.687	0.92	0.803	4
IFNg IL-21 Gzmb Socs1 Tbet	0.679	0.926	0.803	5
LTA Gzmb Socs1 IL-12rb2 Tbet	0.685	0.92	0.803	5
IFNg LTA Socs1 IL-2ra Tbet	0.69	0.916	0.803	5
IFNg LTA IL-12rb2 IL-2ra Tbet	0.684	0.921	0.803	5
IL-21 Gzmb IL-12rb2 IL-2ra Tbet	0.709	0.897	0.803	5
IL-21 LTA Socs1 IL-18bp Tbet	0.714	0.893	0.803	5
IFNg IL-21 Nos2 IL-2ra IL-18bp Tbet	0.665	0.941	0.803	6
IFNg IL-21 LTA Gzmb Socs1 IL-12rb2 IL-2ra Tbet	0.656	0.95	0.803	8
IFNg LTA IL-12rb2	0.671	0.934	0.802	3
Nos2 Socs1 IL-2ra	0.705	0.898	0.802	3
LTA Gzmb Socs1 IL-2ra	0.677	0.927	0.802	4
IFNg IL-21 IL-12rb2 IL-2ra	0.664	0.939	0.802	4
IFNg IL-21 LTA Tbet	0.689	0.914	0.802	4
IFNg Gzmb Nos2 Tbet	0.673	0.93	0.802	4
IL-21 Gzmb Nos2 Tbet	0.704	0.9	0.802	4
IFNg IL-21 IL-2ra Tbet	0.675	0.928	0.802	4
IL-21 Gzmb Socs1 IL-12rb2 Tbet	0.701	0.904	0.802	5
IL-21 Socs1 IL-12rb2 IL-2ra Tbet	0.696	0.909	0.802	5
Nos2 Socs1 IL-12rb2 IL-2ra Tbet	0.717	0.887	0.802	5
Gzmb Nos2 IL-12rb2 IL-18bp Tbet	0.721	0.884	0.802	5
LTA IL-12rb2 IL-2ra IL-18bp Tbet	0.708	0.897	0.802	5
IFNg LTA Nos2 Socs1 IL-12rb2 Tbet	0.666	0.938	0.802	6
IFNg IL-21 LTA Nos2 IL-2ra Tbet	0.666	0.938	0.802	6
IFNg IL-21 Nos2 Socs1 IL-18bp Tbet	0.672	0.933	0.802	6
IFNg IL-21 Gzmb Nos2 Socs1 IL-12rb2 Tbet	0.652	0.951	0.802	7
IFNg IL-21 Gzmb Nos2 Socs1 IL-12rb2 IL-18bp Tbet	0.651	0.953	0.802	8
IFNg LTA Gzmb Nos2 Socs1 IL-12rb2 IL-18bp Tbet	0.657	0.948	0.802	8
IFNg IL-21 LTA Socs1 IL-12rb2 IL-2ra IL-18bp Tbet	0.657	0.948	0.802	8
Gzmb Socs1	0.704	0.898	0.801	2
LTA Nos2 Socs1	0.685	0.918	0.801	3
IFNg IL-12rb2 IL-2ra	0.669	0.932	0.801	3
Nos2 Socs1 IL-18bp	0.703	0.899	0.801	3
IFNg Gzmb Tbet	0.679	0.923	0.801	3
IFNg LTA Nos2 IL-12rb2	0.658	0.944	0.801	4
IL-21 Gzmb Nos2 Socs1 IL-2ra	0.664	0.938	0.801	5
IFNg LTA Socs1 IL-12rb2 Tbet	0.682	0.919	0.801	5
IL-21 LTA Gzmb Socs1 IL-18bp Tbet	0.708	0.894	0.801	6
IFNg LTA Socs1 IL-2ra IL-18bp Tbet	0.683	0.92	0.801	6
IL-21 LTA Gzmb Nos2 Socs1 IL-18bp Tbet	0.694	0.909	0.801	7
IFNg LTA Socs1 IL-12rb2 IL-2ra IL-18bp Tbet	0.67	0.933	0.801	7
IFNg IL-21 Gzmb Nos2 Socs1 IL-12rb2 IL-2ra Tbet	0.637	0.965	0.801	8
LTA Gzmb Nos2 Socs1 IL-12rb2 IL-2ra IL-18bp Tbet	0.652	0.95	0.801	8
IFNg Nos2	0.663	0.937	0.8	2
IFNg IL-12rb2 IL-18bp	0.666	0.935	0.8	3
LTA Nos2 Tbet	0.695	0.905	0.8	3
Gzmb Nos2 Socs1 IL-2ra	0.673	0.927	0.8	4
IFNg Nos2 IL-12rb2 IL-2ra	0.658	0.941	0.8	4
LTA Nos2 Socs1 IL-18bp	0.685	0.916	0.8	4
IFNg LTA Gzmb Tbet	0.681	0.92	0.8	4
Gzmb Socs1 IL-12rb2 Tbet	0.707	0.894	0.8	4
IFNg Gzmb IL-2ra Tbet	0.672	0.928	0.8	4
IFNg Nos2 IL-2ra Tbet	0.669	0.931	0.8	4
Socs1 IL-2ra IL-18bp Tbet	0.732	0.867	0.8	4
LTA Socs1 IL-12rb2 IL-18bp Tbet	0.699	0.9	0.8	5
IFNg IL-21 LTA Gzmb Socs1 IL-18bp	0.671	0.929	0.8	6
IL-21 LTA Nos2 Socs1 IL-12rb2 Tbet	0.673	0.927	0.8	6

IFNg LTA Socs1 IL-12rb2 IL-18bp Tbet	0.672	0.927	0.8	6
IFNg IL-21 LTA Gzmb Socs1 IL-12rb2 IL-18bp	0.641	0.96	0.8	7
IFNg IL-21 LTA Gzmb Socs1 IL-12rb2 Tbet	0.659	0.942	0.8	7
IL-21 LTA Gzmb Socs1 IL-12rb2 IL-2ra Tbet	0.669	0.932	0.8	7
IL-21 LTA Nos2 Socs1 IL-12rb2 IL-2ra Tbet	0.648	0.952	0.8	7
LTA Gzmb Nos2 Socs1 IL-12rb2 IL-2ra Tbet	0.647	0.953	0.8	7
IFNg IL-21 LTA Nos2 Socs1 IL-12rb2 IL-2ra IL-18bp Tbet	0.641	0.959	0.8	9
IFNg IL-21 LTA	0.672	0.926	0.799	3
Gzmb Nos2 Socs1	0.683	0.916	0.799	3
Gzmb Socs1 IL-2ra	0.693	0.905	0.799	3
IFNg IL-21 LTA IL-12rb2	0.662	0.936	0.799	4
IFNg IL-21 IL-12rb2 IL-18bp	0.659	0.938	0.799	4
IFNg IL-21 Gzmb IL-12rb2 IL-2ra	0.646	0.951	0.799	5
IFNg LTA Gzmb IL-12rb2 IL-2ra	0.646	0.952	0.799	5
IFNg IL-21 Nos2 IL-12rb2 IL-2ra	0.653	0.945	0.799	5
IFNg IL-21 Gzmb IL-12rb2 IL-18bp	0.645	0.953	0.799	5
IFNg LTA Gzmb IL-12rb2 IL-18bp	0.646	0.952	0.799	5
LTA Nos2 Socs1 IL-12rb2 IL-18bp	0.681	0.918	0.799	5
IFNg Gzmb IL-12rb2 IL-2ra IL-18bp	0.645	0.953	0.799	5
IFNg IL-21 LTA IL-2ra Tbet	0.674	0.924	0.799	5
IL-21 LTA Nos2 IL-18bp Tbet	0.691	0.908	0.799	5
IL-21 Gzmb Nos2 IL-18bp Tbet	0.696	0.903	0.799	5
IFNg Gzmb Socs1 IL-18bp Tbet	0.673	0.926	0.799	5
LTA Gzmb Nos2 Socs1 IL-12rb2 Tbet	0.663	0.935	0.799	6
IFNg LTA Socs1 IL-12rb2 IL-2ra Tbet	0.677	0.922	0.799	6
IFNg IL-21 LTA Socs1 IL-12rb2 IL-2ra Tbet	0.661	0.937	0.799	7
IFNg IL-21 LTA Gzmb Socs1 IL-18bp Tbet	0.678	0.919	0.799	7
IFNg LTA Gzmb Nos2 Socs1 IL-12rb2 IL-2ra IL-18bp Tbet	0.632	0.967	0.799	9
IFNg LTA Nos2	0.661	0.935	0.798	3
IFNg IL-21 Tbet	0.678	0.918	0.798	3
IL-21 Gzmb Tbet	0.711	0.885	0.798	3
IL-21 LTA Socs1 Tbet	0.706	0.889	0.798	4
IL-21 Gzmb IL-18bp Tbet	0.703	0.894	0.798	4
IFNg Nos2 IL-18bp Tbet	0.668	0.927	0.798	4
IFNg LTA Gzmb Nos2 IL-12rb2	0.645	0.952	0.798	5
IFNg LTA Gzmb Nos2 Tbet	0.668	0.927	0.798	5
IL-21 LTA Gzmb Nos2 Tbet	0.691	0.904	0.798	5
IFNg IL-21 IL-2ra IL-18bp Tbet	0.666	0.93	0.798	5
IFNg IL-21 LTA Socs1 IL-12rb2 Tbet	0.671	0.926	0.798	6
IFNg IL-21 Gzmb Nos2 IL-2ra Tbet	0.658	0.938	0.798	6
IL-21 LTA Gzmb Nos2 Socs1 IL-2ra IL-18bp	0.648	0.947	0.798	7
IFNg IL-21 LTA Gzmb Nos2 Socs1 IL-12rb2 IL-2ra	0.628	0.968	0.798	8
IFNg LTA Gzmb Nos2 Socs1 IL-12rb2 IL-2ra Tbet	0.634	0.963	0.798	8
IL-21 LTA Gzmb Nos2 Socs1 IL-12rb2 IL-2ra Tbet	0.643	0.952	0.798	8
IFNg IL-21 LTA Gzmb Nos2 Socs1 IL-12rb2 IL-2ra IL-18bp	0.624	0.972	0.798	9
IFNg IL-21 LTA Gzmb Nos2 Socs1 IL-12rb2 IL-2ra IL-18bp Tbet	0.631	0.965	0.798	10
IFNg IL-21	0.666	0.928	0.797	2
IFNg Tbet	0.683	0.912	0.797	2
IFNg LTA Gzmb	0.659	0.934	0.797	3
IFNg LTA IL-12rb2 IL-18bp	0.659	0.935	0.797	4
Gzmb Socs1 IL-2ra IL-18bp	0.675	0.919	0.797	4
Nos2 Socs1 IL-2ra IL-18bp	0.693	0.902	0.797	4
IFNg IL-21 Socs1 Tbet	0.677	0.917	0.797	4
IL-21 Gzmb IL-2ra Tbet	0.701	0.893	0.797	4
IL-21 LTA IL-18bp Tbet	0.702	0.893	0.797	4
IFNg Socs1 IL-18bp Tbet	0.678	0.917	0.797	4
IFNg IL-21 LTA Gzmb IL-12rb2	0.642	0.952	0.797	5

IFNg Gzmb Nos2 IL-12rb2 IL-2ra	0.641	0.953	0.797	5
IFNg IL-21 Gzmb Socs1 IL-18bp	0.661	0.932	0.797	5
IFNg Gzmb Nos2 IL-12rb2 IL-18bp	0.643	0.952	0.797	5
IL-21 LTA Gzmb Socs1 Tbet	0.701	0.894	0.797	5
IFNg Nos2 IL-2ra IL-18bp Tbet	0.664	0.93	0.797	5
IFNg IL-21 Gzmb Nos2 Socs1 IL-18bp	0.651	0.942	0.797	6
LTA Socs1 IL-12rb2 IL-2ra IL-18bp Tbet	0.69	0.903	0.797	6
IFNg IL-21 LTA Nos2 Socs1 IL-12rb2 IL-2ra Tbet	0.637	0.957	0.797	8
LTA Gzmb Socs1	0.685	0.907	0.796	3
IL-21 Nos2 Socs1	0.681	0.911	0.796	3
IFNg Nos2 IL-2ra	0.653	0.94	0.796	3
IL-21 LTA Nos2 Socs1	0.674	0.917	0.796	4
IL-21 Gzmb Socs1 IL-2ra	0.679	0.913	0.796	4
IFNg IL-21 Socs1 IL-18bp	0.667	0.925	0.796	4
IFNg Nos2 IL-12rb2 IL-18bp	0.65	0.941	0.796	4
IFNg IL-12rb2 IL-2ra IL-18bp	0.659	0.933	0.796	4
IL-21 LTA Nos2 Socs1 IL-12rb2	0.672	0.919	0.796	5
LTA Gzmb Socs1 IL-2ra IL-18bp	0.664	0.929	0.796	5
Gzmb Nos2 Socs1 IL-2ra IL-18bp	0.661	0.931	0.796	5
IFNg IL-21 Gzmb IL-2ra Tbet	0.662	0.93	0.796	5
IFNg LTA Gzmb IL-2ra Tbet	0.667	0.926	0.796	5
IFNg LTA Nos2 IL-2ra Tbet	0.661	0.931	0.796	5
IL-21 LTA Nos2 IL-2ra Tbet	0.666	0.925	0.796	5
IFNg Gzmb Nos2 IL-2ra Tbet	0.657	0.935	0.796	5
IFNg IL-21 Nos2 IL-18bp Tbet	0.663	0.93	0.796	5
IFNg Gzmb IL-2ra IL-18bp Tbet	0.663	0.928	0.796	5
IL-21 LTA Gzmb Socs1 IL-12rb2 Tbet	0.675	0.917	0.796	6
IFNg IL-21 LTA IL-2ra IL-18bp Tbet	0.665	0.926	0.796	6
IFNg IL-21 LTA Gzmb Nos2 Socs1 IL-18bp	0.658	0.935	0.796	7
IFNg IL-21 LTA Nos2 IL-2ra IL-18bp Tbet	0.656	0.937	0.796	7
IFNg IL-21 LTA Gzmb Nos2 Socs1 IL-18bp Tbet	0.667	0.925	0.796	8
IFNg Gzmb	0.654	0.936	0.795	2
IFNg IL-2ra	0.662	0.927	0.795	2
Nos2 Socs1 IL-12rb2	0.694	0.896	0.795	3
IFNg LTA Tbet	0.681	0.909	0.795	3
LTA IL-12rb2 Tbet	0.708	0.882	0.795	3
IFNg LTA IL-12rb2 IL-2ra	0.66	0.931	0.795	4
Gzmb Nos2 Socs1 IL-18bp	0.673	0.916	0.795	4
IFNg IL-21 LTA Nos2 IL-12rb2	0.647	0.942	0.795	5
IFNg IL-21 Gzmb Nos2 IL-12rb2	0.638	0.952	0.795	5
IFNg IL-21 IL-12rb2 IL-2ra IL-18bp	0.65	0.94	0.795	5
Nos2 IL-12rb2 IL-2ra IL-18bp Tbet	0.732	0.858	0.795	5
IL-21 LTA Nos2 Socs1 IL-12rb2 IL-2ra	0.643	0.947	0.795	6
IL-21 Gzmb Nos2 Socs1 IL-2ra IL-18bp	0.651	0.938	0.795	6
IFNg LTA Gzmb IL-12rb2 IL-2ra IL-18bp	0.637	0.953	0.795	6
IFNg IL-21 LTA Gzmb Nos2 Socs1 IL-12rb2	0.634	0.956	0.795	7
IFNg IL-21 Nos2	0.652	0.937	0.794	3
Gzmb Socs1 IL-18bp	0.682	0.907	0.794	3
LTA Gzmb Nos2 Socs1	0.664	0.923	0.794	4
IL-12rb2 IL-2ra IL-18bp Tbet	0.743	0.844	0.794	4
IFNg IL-21 LTA IL-12rb2 IL-2ra	0.651	0.937	0.794	5
IL-21 LTA Nos2 Socs1 IL-18bp	0.673	0.915	0.794	5
IFNg IL-21 LTA IL-12rb2 IL-18bp	0.651	0.937	0.794	5
IFNg IL-21 Gzmb Nos2 Tbet	0.66	0.929	0.794	5
IL-21 LTA Socs1 IL-12rb2 Tbet	0.685	0.903	0.794	5
IFNg LTA Nos2 IL-18bp Tbet	0.663	0.925	0.794	5
IFNg IL-21 LTA Gzmb IL-12rb2 IL-18bp	0.636	0.953	0.794	6

IFNg IL-21 Gzmb IL-12rb2 IL-2ra IL-18bp	0.636	0.953	0.794	6
IFNg LTA Nos2 IL-2ra IL-18bp Tbet	0.658	0.93	0.794	6
IFNg IL-21 LTA Nos2 Socs1 IL-12rb2 Tbet	0.651	0.937	0.794	7
IFNg IL-21 LTA Gzmb Nos2 Socs1 IL-12rb2 IL-18bp	0.628	0.959	0.794	8
IFNg IL-21 LTA Gzmb Nos2 Socs1 IL-12rb2 IL-2ra Tbet	0.627	0.961	0.794	9
IFNg IL-2ra Tbet	0.673	0.912	0.793	3
IL-21 Nos2 Socs1 IL-18bp	0.676	0.91	0.793	4
Nos2 Socs1 IL-12rb2 IL-18bp	0.689	0.896	0.793	4
IFNg IL-21 Gzmb Tbet	0.665	0.921	0.793	4
LTA Nos2 IL-18bp Tbet	0.682	0.903	0.793	4
IFNg LTA IL-12rb2 IL-2ra IL-18bp	0.654	0.932	0.793	5
Gzmb Socs1 IL-12rb2 IL-2ra Tbet	0.694	0.892	0.793	5
IFNg IL-21 LTA Gzmb IL-12rb2 IL-2ra	0.635	0.952	0.793	6
IFNg IL-21 Gzmb Nos2 IL-12rb2 IL-18bp	0.634	0.952	0.793	6
IFNg Gzmb Nos2 IL-12rb2 IL-2ra IL-18bp	0.634	0.953	0.793	6
IL-21 LTA Gzmb Nos2 IL-18bp Tbet	0.682	0.903	0.793	6
IFNg Gzmb Nos2 IL-2ra IL-18bp Tbet	0.651	0.934	0.793	6
IFNg LTA Gzmb Nos2 Socs1 IL-12rb2 Tbet	0.641	0.946	0.793	7
IFNg IL-21 Gzmb Nos2 Socs1 IL-18bp Tbet	0.651	0.934	0.793	7
IFNg IL-21 LTA Gzmb Socs1 IL-12rb2 IL-18bp Tbet	0.638	0.948	0.793	8
IFNg IL-21 IL-2ra	0.652	0.933	0.792	3
IL-21 LTA Tbet	0.695	0.889	0.792	3
IFNg IL-21 LTA Nos2	0.651	0.933	0.792	4
LTA Gzmb Nos2 Tbet	0.678	0.907	0.792	4
IFNg Gzmb IL-18bp Tbet	0.661	0.922	0.792	4
IL-21 LTA Gzmb Nos2 Socs1	0.663	0.921	0.792	5
IFNg IL-21 Nos2 IL-12rb2 IL-18bp	0.643	0.942	0.792	5
IFNg Gzmb Nos2 IL-18bp Tbet	0.655	0.929	0.792	5
IL-21 LTA IL-2ra IL-18bp Tbet	0.675	0.908	0.792	5
IL-21 LTA Nos2 Socs1 IL-12rb2 IL-18bp	0.667	0.917	0.792	6
IL-21 LTA Gzmb Nos2 IL-2ra Tbet	0.665	0.919	0.792	6
IL-21 LTA Socs1 IL-12rb2 IL-2ra Tbet	0.667	0.917	0.792	6
IFNg IL-21 Gzmb Socs1 IL-18bp Tbet	0.66	0.924	0.792	6
IFNg IL-21 Gzmb IL-2ra IL-18bp Tbet	0.652	0.931	0.792	6
IFNg LTA Gzmb IL-2ra IL-18bp Tbet	0.659	0.926	0.792	6
IL-21 LTA Nos2 Socs1 IL-12rb2 IL-2ra IL-18bp	0.638	0.945	0.792	7
IFNg IL-21 Gzmb Nos2 IL-2ra IL-18bp Tbet	0.647	0.937	0.792	7
IL-21 Gzmb Socs1	0.685	0.896	0.791	3
IFNg IL-18bp Tbet	0.669	0.913	0.791	3
IFNg IL-21 LTA IL-2ra	0.652	0.93	0.791	4
IFNg IL-21 Nos2 IL-2ra	0.64	0.943	0.791	4
LTA Socs1 IL-12rb2 Tbet	0.698	0.885	0.791	4
IL-21 LTA IL-2ra Tbet	0.68	0.902	0.791	4
LTA IL-12rb2 IL-2ra Tbet	0.704	0.877	0.791	4
LTA Socs1 IL-18bp Tbet	0.703	0.88	0.791	4
IFNg LTA Nos2 IL-12rb2 IL-2ra	0.639	0.943	0.791	5
IFNg IL-21 LTA Gzmb Tbet	0.667	0.915	0.791	5
IFNg IL-21 LTA IL-18bp Tbet	0.667	0.915	0.791	5
IFNg LTA Gzmb IL-18bp Tbet	0.662	0.919	0.791	5
IFNg IL-21 Gzmb Nos2 IL-12rb2 IL-2ra	0.629	0.952	0.791	6
IFNg LTA Gzmb Nos2 IL-12rb2 IL-18bp	0.631	0.952	0.791	6
IFNg IL-21 LTA IL-12rb2 IL-2ra IL-18bp	0.645	0.937	0.791	6
IFNg IL-21 LTA Gzmb Nos2 Tbet	0.661	0.921	0.791	6
IFNg LTA Gzmb Nos2 IL-2ra Tbet	0.648	0.935	0.791	6
IFNg IL-21 LTA Gzmb IL-12rb2 IL-2ra IL-18bp	0.628	0.953	0.791	7
IL-21 LTA Gzmb Nos2 Socs1 IL-12rb2 Tbet	0.652	0.93	0.791	7
IFNg Gzmb Nos2	0.639	0.94	0.79	3

IFNg LTA IL-2ra	0.657	0.923	0.79	3
IFNg LTA Nos2 IL-2ra	0.643	0.938	0.79	4
IFNg LTA IL-2ra Tbet	0.67	0.909	0.79	4
IFNg IL-21 IL-18bp Tbet	0.662	0.918	0.79	4
IFNg IL-2ra IL-18bp Tbet	0.667	0.914	0.79	4
IFNg LTA Nos2 IL-12rb2 IL-18bp	0.639	0.942	0.79	5
IL-21 Gzmb Socs1 IL-2ra IL-18bp	0.659	0.921	0.79	5
IFNg Nos2 IL-12rb2 IL-2ra IL-18bp	0.642	0.939	0.79	5
IFNg LTA Gzmb Nos2 IL-12rb2 IL-2ra	0.627	0.953	0.79	6
LTA Gzmb Nos2 Socs1 IL-12rb2 IL-2ra	0.623	0.958	0.79	6
IFNg IL-21 LTA Gzmb IL-2ra Tbet	0.656	0.924	0.79	6
IFNg IL-21 LTA Nos2 IL-18bp Tbet	0.657	0.924	0.79	6
IL-21 LTA Nos2 IL-2ra IL-18bp Tbet	0.657	0.923	0.79	6
IFNg IL-21 LTA Gzmb Nos2 IL-2ra Tbet	0.646	0.934	0.79	7
IFNg IL-18bp	0.654	0.924	0.789	2
IFNg Gzmb IL-2ra	0.637	0.94	0.789	3
IFNg LTA Gzmb Nos2	0.642	0.937	0.789	4
IL-21 LTA Gzmb Socs1	0.676	0.903	0.789	4
IL-21 Gzmb Nos2 Socs1	0.665	0.914	0.789	4
LTA Gzmb Socs1 IL-12rb2	0.656	0.921	0.789	4
LTA Nos2 IL-2ra Tbet	0.67	0.909	0.789	4
IL-21 LTA Gzmb Socs1 IL-2ra	0.653	0.924	0.789	5
LTA Gzmb Nos2 Socs1 IL-18bp	0.657	0.921	0.789	5
IFNg IL-21 LTA Gzmb Nos2 IL-12rb2	0.626	0.953	0.789	6
IFNg IL-21 Gzmb Nos2 IL-12rb2 IL-2ra IL-18bp	0.625	0.953	0.789	7
IFNg Nos2 IL-18bp	0.642	0.933	0.788	3
LTA Socs1 Tbet	0.7	0.875	0.788	3
IFNg LTA IL-18bp Tbet	0.667	0.91	0.788	4
IFNg IL-21 Socs1 IL-18bp Tbet	0.658	0.917	0.788	5
IFNg IL-21 Nos2 IL-12rb2 IL-2ra IL-18bp	0.632	0.944	0.788	6
IFNg LTA Gzmb Nos2 IL-2ra IL-18bp Tbet	0.643	0.933	0.788	7
IFNg IL-21 LTA Gzmb Nos2 Socs1 IL-12rb2 Tbet	0.631	0.946	0.788	8
IFNg IL-21 Gzmb	0.639	0.935	0.787	3
LTA Gzmb Nos2 Socs1 IL-12rb2	0.64	0.933	0.787	5
IFNg IL-21 LTA Nos2 IL-2ra	0.635	0.939	0.787	5
IL-21 Nos2 Socs1 IL-12rb2 IL-2ra	0.64	0.933	0.787	5
IFNg LTA IL-2ra IL-18bp Tbet	0.663	0.911	0.787	5
IFNg IL-21 LTA Nos2 IL-12rb2 IL-2ra	0.629	0.944	0.787	6
IFNg LTA Gzmb Nos2 IL-18bp Tbet	0.648	0.926	0.787	6
IFNg IL-21 LTA Gzmb Nos2 IL-12rb2 IL-18bp	0.622	0.953	0.787	7
IFNg LTA Gzmb Nos2 IL-12rb2 IL-2ra IL-18bp	0.621	0.953	0.787	7
IFNg IL-21 LTA Gzmb Nos2 Socs1 IL-12rb2 IL-18bp Tbet	0.626	0.947	0.787	9
IL-21 Socs1 IL-2ra	0.672	0.899	0.786	3
IFNg LTA IL-18bp	0.651	0.922	0.786	3
Gzmb IL-18bp Tbet	0.705	0.867	0.786	3
IL-21 Nos2 Socs1 IL-12rb2	0.662	0.91	0.786	4
IFNg LTA Gzmb IL-2ra	0.635	0.937	0.786	4
LTA Gzmb Socs1 IL-18bp	0.665	0.908	0.786	4
IL-21 LTA Gzmb IL-2ra Tbet	0.67	0.902	0.786	5
LTA Socs1 IL-12rb2 IL-2ra Tbet	0.689	0.884	0.786	5
IL-21 LTA Gzmb IL-18bp Tbet	0.681	0.892	0.786	5
LTA Nos2 IL-2ra IL-18bp Tbet	0.665	0.907	0.786	5
IL-21 LTA Gzmb Nos2 Socs1 IL-18bp	0.654	0.918	0.786	6
IFNg LTA Nos2 IL-12rb2 IL-2ra IL-18bp	0.632	0.941	0.786	6
IFNg IL-21 LTA Gzmb IL-2ra IL-18bp Tbet	0.647	0.925	0.786	7
IL-21 LTA Gzmb Nos2 IL-2ra IL-18bp Tbet	0.652	0.919	0.786	7
IFNg IL-2ra IL-18bp	0.647	0.924	0.785	3

LTA Gzmb Tbet	0.681	0.89	0.785	3
IFNg IL-21 LTA Gzmb	0.637	0.932	0.785	4
Nos2 Socs1 IL-12rb2 IL-2ra	0.673	0.897	0.785	4
IL-21 Socs1 IL-2ra IL-18bp	0.658	0.912	0.785	4
IL-21 LTA Gzmb Tbet	0.679	0.89	0.785	4
LTA Socs1 IL-2ra Tbet	0.691	0.878	0.785	4
Nos2 IL-12rb2 IL-18bp Tbet	0.72	0.851	0.785	4
IFNg IL-21 Gzmb IL-18bp Tbet	0.649	0.921	0.785	5
LTA Socs1 IL-2ra IL-18bp Tbet	0.683	0.886	0.785	5
IFNg IL-21 LTA Nos2 IL-12rb2 IL-18bp	0.628	0.941	0.785	6
IFNg IL-21 Gzmb Nos2 IL-18bp Tbet	0.643	0.927	0.785	6
IL-21 LTA Gzmb IL-2ra IL-18bp Tbet	0.663	0.907	0.785	6
IFNg IL-21 LTA Gzmb Nos2 IL-12rb2 IL-2ra	0.617	0.953	0.785	7
Socs1 IL-2ra Tbet	0.734	0.834	0.784	3
IL-12rb2 IL-18bp Tbet	0.736	0.831	0.784	3
IFNg Gzmb Nos2 IL-2ra	0.623	0.945	0.784	4
LTA Gzmb Nos2 IL-18bp Tbet	0.664	0.904	0.784	5
IFNg IL-21 LTA Gzmb Nos2 IL-2ra IL-18bp Tbet	0.636	0.932	0.784	8
IFNg IL-21 IL-18bp	0.641	0.925	0.783	3
Nos2 IL-18bp Tbet	0.715	0.851	0.783	3
IFNg LTA Nos2 IL-18bp	0.634	0.932	0.783	4
IFNg Nos2 IL-2ra IL-18bp	0.631	0.935	0.783	4
Gzmb Nos2 IL-18bp Tbet	0.695	0.871	0.783	4
Nos2 IL-2ra IL-18bp Tbet	0.71	0.855	0.783	4
IL-21 Gzmb Nos2 Socs1 IL-18bp	0.655	0.912	0.783	5
IL-21 LTA Socs1 IL-2ra IL-18bp	0.651	0.915	0.783	5
Nos2 Socs1 IL-12rb2 IL-2ra IL-18bp	0.668	0.898	0.783	5
IL-21 Nos2 Socs1 IL-12rb2 IL-2ra IL-18bp	0.633	0.934	0.783	6
LTA Gzmb Nos2 Socs1 IL-12rb2 IL-2ra IL-18bp	0.611	0.955	0.783	7
IFNg IL-21 LTA Gzmb Nos2 IL-12rb2 IL-2ra IL-18bp	0.614	0.953	0.783	8
Nos2 IL-2ra Tbet	0.722	0.842	0.782	3
IL-21 Gzmb Socs1 IL-18bp	0.663	0.9	0.782	4
IFNg IL-21 IL-2ra IL-18bp	0.633	0.931	0.782	4
IFNg LTA IL-2ra IL-18bp	0.644	0.921	0.782	4
LTA Gzmb IL-2ra Tbet	0.67	0.894	0.782	4
IL-21 Nos2 Socs1 IL-12rb2 IL-18bp	0.655	0.909	0.782	5
LTA Gzmb Nos2 IL-2ra Tbet	0.65	0.913	0.782	5
IFNg IL-21 LTA Nos2 IL-12rb2 IL-2ra IL-18bp	0.62	0.943	0.782	7
IL-21 Socs1	0.679	0.883	0.781	2
LTA Tbet	0.692	0.871	0.781	2
Socs1 Tbet	0.728	0.834	0.781	2
Gzmb Socs1 IL-12rb2	0.659	0.903	0.781	3
IFNg Gzmb IL-18bp	0.628	0.933	0.781	3
Gzmb Nos2 Socs1 IL-12rb2	0.644	0.919	0.781	4
IFNg IL-21 Gzmb IL-2ra	0.624	0.939	0.781	4
IL-21 LTA Socs1 IL-2ra	0.652	0.909	0.781	4
IFNg IL-21 LTA IL-18bp	0.638	0.924	0.781	4
IFNg LTA Gzmb IL-18bp	0.63	0.932	0.781	4
IFNg IL-21 Nos2 IL-18bp	0.628	0.933	0.781	4
Gzmb IL-2ra IL-18bp Tbet	0.697	0.865	0.781	4
IL-18bp Tbet	0.729	0.831	0.78	2
Nos2 IL-12rb2 IL-2ra Tbet	0.721	0.839	0.78	4
LTA Gzmb IL-18bp Tbet	0.671	0.889	0.78	4
IFNg IL-21 LTA IL-2ra IL-18bp	0.631	0.928	0.78	5
LTA Gzmb Socs1 IL-12rb2 IL-2ra IL-18bp	0.617	0.942	0.78	6
LTA Socs1	0.68	0.878	0.779	2
IFNg IL-21 Gzmb Nos2	0.619	0.939	0.779	4

IFNg Gzmb Nos2 IL-18bp	0.621	0.937	0.779	4
Gzmb Socs1 IL-12rb2 IL-18bp	0.644	0.915	0.779	4
IFNg LTA Gzmb Nos2 IL-2ra	0.616	0.942	0.779	5
LTA Gzmb Socs1 IL-12rb2 IL-2ra	0.62	0.938	0.779	5
IL-21 LTA Gzmb Socs1 IL-18bp	0.655	0.903	0.779	5
Gzmb Nos2 IL-12rb2 IL-2ra Tbet	0.703	0.856	0.779	5
IL-21 Gzmb Nos2 Socs1 IL-12rb2 IL-2ra	0.62	0.937	0.779	6
IFNg IL-21 LTA Gzmb IL-18bp Tbet	0.644	0.915	0.779	6
IL-2ra IL-18bp Tbet	0.718	0.838	0.778	3
IFNg Gzmb IL-2ra IL-18bp	0.619	0.938	0.778	4
Gzmb Nos2 Socs1 IL-12rb2 IL-2ra	0.63	0.926	0.778	5
IFNg IL-21 Nos2 IL-2ra IL-18bp	0.618	0.939	0.778	5
IFNg LTA Nos2 IL-2ra IL-18bp	0.621	0.935	0.778	5
Gzmb Nos2 IL-2ra IL-18bp Tbet	0.688	0.869	0.778	5
IL-21 LTA Gzmb Socs1 IL-2ra IL-18bp	0.63	0.926	0.778	6
IL-21 LTA Socs1	0.667	0.888	0.777	3
LTA IL-2ra Tbet	0.686	0.869	0.777	3
LTA IL-18bp Tbet	0.677	0.876	0.777	3
Gzmb Nos2 IL-2ra Tbet	0.697	0.857	0.777	4
Socs1 IL-12rb2 IL-2ra Tbet	0.725	0.83	0.777	4
Gzmb Socs1 IL-12rb2 IL-2ra IL-18bp	0.631	0.924	0.777	5
IL-21 LTA Gzmb Nos2 Socs1 IL-12rb2	0.62	0.934	0.777	6
IL-21 Socs1 IL-12rb2	0.67	0.882	0.776	3
IL-21 Socs1 IL-18bp	0.664	0.889	0.776	3
LTA Socs1 IL-18bp	0.671	0.882	0.776	3
Gzmb Nos2 Tbet	0.694	0.857	0.776	3
IFNg IL-21 Gzmb IL-18bp	0.619	0.932	0.776	4
LTA Gzmb Socs1 IL-12rb2 IL-18bp	0.631	0.922	0.776	5
IFNg LTA Gzmb IL-2ra IL-18bp	0.617	0.935	0.776	5
LTA Gzmb Nos2 Socs1 IL-12rb2 IL-18bp	0.622	0.93	0.776	6
LTA Gzmb Nos2 IL-2ra IL-18bp Tbet	0.642	0.91	0.776	6
Gzmb Socs1 IL-12rb2 IL-2ra	0.645	0.905	0.775	4
IFNg IL-21 LTA Gzmb Nos2	0.614	0.936	0.775	5
IFNg LTA Gzmb Nos2 IL-18bp	0.616	0.935	0.775	5
Gzmb Nos2 Socs1 IL-12rb2 IL-18bp	0.63	0.92	0.775	5
IFNg Gzmb Nos2 IL-2ra IL-18bp	0.609	0.941	0.775	5
LTA Gzmb IL-2ra IL-18bp Tbet	0.657	0.894	0.775	5
IFNg IL-21 LTA Gzmb Nos2 IL-18bp Tbet	0.631	0.919	0.775	7
IL-21 LTA Socs1 IL-18bp	0.657	0.89	0.774	4
LTA Socs1 IL-12rb2 IL-18bp	0.663	0.885	0.774	4
IL-21 LTA Gzmb Socs1 IL-12rb2	0.628	0.92	0.774	5
IFNg IL-21 LTA Gzmb IL-2ra	0.613	0.936	0.774	5
IFNg IL-21 Gzmb Nos2 IL-2ra	0.604	0.944	0.774	5
Gzmb Nos2 Socs1 IL-12rb2 IL-2ra IL-18bp	0.615	0.932	0.774	6
Socs1 IL-12rb2 Tbet	0.713	0.834	0.773	3
IL-21 Socs1 IL-12rb2 IL-18bp	0.657	0.889	0.773	4
LTA IL-2ra IL-18bp Tbet	0.67	0.876	0.773	4
IL-21 Gzmb Socs1 IL-12rb2 IL-2ra	0.632	0.913	0.773	5
IFNg IL-21 LTA Gzmb IL-18bp	0.615	0.93	0.773	5
IFNg IL-21 LTA Nos2 IL-18bp	0.616	0.931	0.773	5
IFNg IL-21 Gzmb IL-2ra IL-18bp	0.61	0.936	0.773	5
IL-21 Socs1 IL-12rb2 IL-2ra IL-18bp	0.637	0.908	0.773	5
IL-21 LTA Gzmb Socs1 IL-12rb2 IL-2ra	0.611	0.935	0.773	6
IL-21 LTA Socs1 IL-12rb2 IL-2ra IL-18bp	0.632	0.915	0.773	6
LTA Socs1 IL-12rb2	0.664	0.88	0.772	3
IL-21 LTA Socs1 IL-12rb2	0.654	0.891	0.772	4
IL-21 Gzmb Socs1 IL-12rb2	0.643	0.901	0.772	4

IL-21 Socs1 IL-12rb2 IL-2ra	0.648	0.897	0.772	4
Gzmb Nos2 IL-12rb2 Tbet	0.687	0.856	0.772	4
IL-21 Gzmb Nos2 Socs1 IL-12rb2	0.627	0.917	0.772	5
IFNg IL-21 LTA Nos2 IL-2ra IL-18bp	0.607	0.937	0.772	6
IL-21 LTA Gzmb Nos2 Socs1 IL-12rb2 IL-2ra	0.587	0.957	0.772	7
Nos2 Tbet	0.718	0.824	0.771	2
IFNg IL-21 Gzmb Nos2 IL-18bp	0.607	0.936	0.771	5
IL-21 LTA Socs1 IL-12rb2 IL-18bp	0.648	0.894	0.771	5
IFNg IL-21 LTA Gzmb IL-2ra IL-18bp	0.607	0.934	0.77	6
IFNg LTA Gzmb Nos2 IL-2ra IL-18bp	0.599	0.94	0.77	6
IL-21 Gzmb Socs1 IL-12rb2 IL-2ra IL-18bp	0.616	0.925	0.77	6
Gzmb IL-2ra Tbet	0.694	0.844	0.769	3
IFNg IL-21 Gzmb Nos2 IL-2ra IL-18bp	0.597	0.941	0.769	6
IL-21 Gzmb Nos2 Socs1 IL-12rb2 IL-2ra IL-18bp	0.601	0.937	0.769	7
Socs1 IL-18bp	0.675	0.861	0.768	2
Nos2 IL-12rb2 Tbet	0.707	0.83	0.768	3
Gzmb IL-12rb2 IL-2ra Tbet	0.693	0.843	0.768	4
IL-21 LTA Gzmb Nos2 Socs1 IL-12rb2 IL-18bp	0.608	0.928	0.768	7
LTA Socs1 IL-2ra	0.65	0.883	0.767	3
IL-21 Gzmb Socs1 IL-12rb2 IL-18bp	0.626	0.909	0.767	5
LTA Socs1 IL-2ra IL-18bp	0.641	0.891	0.766	4
IL-21 LTA Socs1 IL-12rb2 IL-2ra	0.624	0.909	0.766	5
IFNg IL-21 LTA Gzmb Nos2 IL-2ra	0.59	0.941	0.766	6
Gzmb Tbet	0.685	0.844	0.765	2
IFNg IL-21 LTA Gzmb Nos2 IL-18bp	0.597	0.933	0.765	6
Socs1 IL-12rb2 IL-18bp	0.67	0.857	0.764	3
IL-2ra Tbet	0.726	0.801	0.763	2
IFNg IL-21 LTA Gzmb Nos2 IL-2ra IL-18bp	0.588	0.939	0.763	7
Socs1 IL-2ra IL-18bp	0.667	0.858	0.762	3
LTA Socs1 IL-12rb2 IL-2ra IL-18bp	0.632	0.892	0.762	5
IL-21 LTA Gzmb Socs1 IL-12rb2 IL-18bp	0.604	0.919	0.762	6
IL-21 Gzmb Nos2 Socs1 IL-12rb2 IL-18bp	0.609	0.915	0.762	6
IL-21 LTA Gzmb Nos2 Socs1 IL-12rb2 IL-2ra IL-18bp	0.57	0.952	0.761	8
Socs1 IL-2ra	0.691	0.826	0.759	2
Gzmb IL-12rb2 Tbet	0.675	0.844	0.759	3
LTA Socs1 IL-12rb2 IL-2ra	0.636	0.882	0.759	4
Socs1 IL-12rb2 IL-2ra IL-18bp	0.663	0.853	0.758	4
IL-12rb2 IL-2ra Tbet	0.716	0.797	0.757	3
IL-21 LTA Gzmb Socs1 IL-12rb2 IL-2ra IL-18bp	0.574	0.937	0.756	7
Socs1 IL-12rb2	0.678	0.832	0.755	2
Socs1 IL-12rb2 IL-2ra	0.681	0.827	0.754	3
Socs1	0.681	0.825	0.753	1
LTA Nos2 IL-12rb2	0.62	0.886	0.753	3
LTA Gzmb Nos2 IL-12rb2	0.604	0.899	0.752	4
LTA Nos2	0.614	0.886	0.75	2
LTA Gzmb IL-12rb2	0.608	0.889	0.748	3
LTA Nos2 IL-18bp	0.608	0.884	0.746	3
IL-21 LTA Nos2 IL-12rb2	0.606	0.886	0.746	4
LTA Nos2 IL-2ra	0.603	0.887	0.745	3
LTA Nos2 IL-12rb2 IL-2ra	0.605	0.885	0.745	4
LTA Nos2 IL-12rb2 IL-18bp	0.606	0.884	0.745	4
LTA Gzmb Nos2 IL-12rb2 IL-18bp	0.59	0.897	0.743	5
IL-21 LTA Nos2	0.597	0.886	0.742	3
LTA Gzmb Nos2	0.591	0.893	0.742	3
LTA Gzmb	0.602	0.88	0.741	2
LTA Gzmb IL-12rb2 IL-18bp	0.592	0.888	0.74	4
LTA Gzmb Nos2 IL-12rb2 IL-2ra	0.578	0.901	0.739	5

Tbet	0.728	0.749	0.738	1
LTA Gzmb Nos2 IL-18bp	0.585	0.892	0.738	4
IL-21 LTA Nos2 IL-12rb2 IL-2ra	0.587	0.889	0.738	5
IL-12rb2 Tbet	0.697	0.778	0.737	2
IL-21 LTA Nos2 IL-2ra	0.583	0.891	0.737	4
LTA Gzmb IL-12rb2 IL-2ra	0.584	0.889	0.737	4
LTA Gzmb Nos2 IL-2ra	0.576	0.897	0.736	4
IL-21 LTA Nos2 IL-18bp	0.585	0.884	0.735	4
IL-21 LTA Gzmb Nos2	0.577	0.891	0.734	4
IL-21 LTA Gzmb IL-12rb2	0.581	0.888	0.734	4
IL-21 LTA Nos2 IL-12rb2 IL-18bp	0.585	0.884	0.734	5
IL-21 LTA	0.6	0.863	0.732	2
LTA Nos2 IL-2ra IL-18bp	0.58	0.885	0.732	4
IL-21 LTA Gzmb Nos2 IL-12rb2	0.565	0.898	0.732	5
LTA Nos2 IL-12rb2 IL-2ra IL-18bp	0.581	0.883	0.732	5
LTA	0.609	0.851	0.73	1
LTA Gzmb IL-2ra	0.579	0.882	0.73	3
LTA Gzmb IL-18bp	0.579	0.88	0.73	3
IL-21 LTA Gzmb IL-12rb2 IL-2ra	0.571	0.887	0.729	5
LTA Gzmb IL-12rb2 IL-2ra IL-18bp	0.569	0.889	0.729	5
LTA Gzmb Nos2 IL-12rb2 IL-2ra IL-18bp	0.56	0.898	0.729	6
IL-21 LTA Gzmb	0.577	0.878	0.728	3
IL-21 LTA IL-12rb2	0.592	0.864	0.728	3
IL-21 LTA Gzmb IL-12rb2 IL-18bp	0.568	0.887	0.728	5
IL-21 LTA Gzmb Nos2 IL-12rb2 IL-18bp	0.56	0.895	0.727	6
IL-21 LTA Gzmb Nos2 IL-2ra	0.557	0.896	0.726	5
IL-21 LTA Gzmb Nos2 IL-12rb2 IL-2ra	0.551	0.9	0.726	6
LTA IL-12rb2	0.597	0.851	0.724	2
LTA Gzmb Nos2 IL-2ra IL-18bp	0.551	0.895	0.723	5
IL-21 LTA Gzmb IL-12rb2 IL-2ra IL-18bp	0.559	0.887	0.723	6
LTA IL-2ra	0.598	0.846	0.722	2
IL-21 LTA IL-2ra	0.58	0.865	0.722	3
IL-21 LTA Nos2 IL-2ra IL-18bp	0.556	0.889	0.722	5
IL-21 LTA Nos2 IL-12rb2 IL-2ra IL-18bp	0.557	0.887	0.722	6
IL-21 LTA Gzmb Nos2 IL-12rb2 IL-2ra IL-18bp	0.547	0.897	0.722	7
IL-21 LTA IL-18bp	0.577	0.863	0.72	3
IL-21 LTA Gzmb Nos2 IL-18bp	0.552	0.889	0.72	5
IL-21 Nos2	0.589	0.848	0.719	2
LTA IL-12rb2 IL-2ra	0.594	0.845	0.719	3
IL-21 LTA IL-12rb2 IL-18bp	0.576	0.863	0.719	4
LTA IL-18bp	0.584	0.851	0.718	2
IL-21 LTA Gzmb IL-2ra	0.557	0.88	0.718	4
LTA IL-12rb2 IL-18bp	0.583	0.851	0.717	3
IL-21 LTA IL-12rb2 IL-2ra	0.571	0.864	0.717	4
LTA Gzmb IL-2ra IL-18bp	0.553	0.881	0.717	4
IL-21 Nos2 IL-12rb2	0.581	0.849	0.715	3
IL-21 LTA Gzmb Nos2 IL-2ra IL-18bp	0.537	0.894	0.715	6
IL-21 Nos2 IL-18bp	0.582	0.847	0.714	3
IL-21	0.593	0.832	0.713	1
IL-21 Nos2 IL-2ra	0.581	0.846	0.713	3
IL-21 LTA Gzmb IL-18bp	0.549	0.877	0.713	4
LTA IL-12rb2 IL-2ra IL-18bp	0.581	0.846	0.713	4
LTA IL-2ra IL-18bp	0.578	0.847	0.712	3
IL-21 IL-18bp	0.584	0.836	0.71	2
IL-21 Nos2 IL-12rb2 IL-18bp	0.574	0.846	0.71	4
IL-21 IL-12rb2	0.586	0.833	0.709	2
IL-21 IL-2ra	0.589	0.829	0.709	2

IL-21 LTA IL-2ra IL-18bp	0.553	0.865	0.709	4
IL-21 Nos2 IL-2ra IL-18bp	0.574	0.844	0.709	4
IL-21 LTA Gzmb IL-2ra IL-18bp	0.539	0.88	0.709	5
IL-21 LTA IL-12rb2 IL-2ra IL-18bp	0.554	0.863	0.709	5
IL-21 Gzmb Nos2	0.567	0.849	0.708	3
IL-21 Nos2 IL-12rb2 IL-2ra	0.571	0.845	0.708	4
IL-21 IL-12rb2 IL-18bp	0.579	0.834	0.706	3
IL-21 Gzmb	0.568	0.842	0.705	2
IL-21 IL-2ra IL-18bp	0.577	0.832	0.705	3
IL-21 IL-12rb2 IL-2ra	0.578	0.829	0.704	3
IL-21 Gzmb Nos2 IL-12rb2	0.558	0.849	0.704	4
IL-21 Gzmb Nos2 IL-2ra	0.561	0.847	0.704	4
IL-21 Nos2 IL-12rb2 IL-2ra IL-18bp	0.565	0.843	0.704	5
IL-21 Gzmb Nos2 IL-18bp	0.557	0.848	0.702	4
IL-21 Gzmb IL-12rb2	0.561	0.841	0.701	3
IL-21 Gzmb IL-2ra	0.565	0.837	0.701	3
Nos2 IL-2ra	0.614	0.785	0.7	2
IL-21 IL-12rb2 IL-2ra IL-18bp	0.571	0.83	0.7	4
IL-21 Gzmb IL-18bp	0.554	0.845	0.699	3
Gzmb Nos2 IL-2ra	0.576	0.821	0.698	3
IL-21 Gzmb Nos2 IL-12rb2 IL-2ra	0.551	0.845	0.698	5
Gzmb IL-2ra	0.581	0.813	0.697	2
IL-21 Gzmb Nos2 IL-12rb2 IL-18bp	0.549	0.846	0.697	5
IL-21 Gzmb Nos2 IL-2ra IL-18bp	0.551	0.844	0.697	5
IL-21 Gzmb IL-12rb2 IL-2ra	0.556	0.836	0.696	4
IL-21 Gzmb IL-12rb2 IL-18bp	0.548	0.843	0.696	4
Nos2 IL-2ra IL-18bp	0.604	0.785	0.694	3
IL-21 Gzmb IL-2ra IL-18bp	0.548	0.841	0.694	4
Gzmb IL-2ra IL-18bp	0.562	0.822	0.692	3
Gzmb Nos2 IL-12rb2 IL-2ra	0.564	0.819	0.692	4
Gzmb IL-12rb2 IL-2ra	0.571	0.811	0.691	3
Gzmb Nos2 IL-2ra IL-18bp	0.56	0.822	0.691	4
IL-21 Gzmb Nos2 IL-12rb2 IL-2ra IL-18bp	0.541	0.842	0.691	6
Nos2 IL-12rb2 IL-2ra	0.588	0.793	0.69	3
IL-21 Gzmb IL-12rb2 IL-2ra IL-18bp	0.542	0.838	0.69	5
IL-2ra	0.636	0.741	0.689	1
Gzmb IL-12rb2 IL-2ra IL-18bp	0.557	0.819	0.688	4
Nos2 IL-12rb2 IL-2ra IL-18bp	0.585	0.79	0.687	4
Gzmb IL-18bp	0.557	0.815	0.686	2
Gzmb Nos2 IL-12rb2 IL-2ra IL-18bp	0.553	0.82	0.686	5
IL-2ra IL-18bp	0.611	0.757	0.684	2
Gzmb Nos2 IL-18bp	0.555	0.814	0.684	3
Gzmb Nos2 IL-12rb2	0.554	0.81	0.682	3
Gzmb IL-12rb2 IL-18bp	0.549	0.813	0.681	3
Gzmb Nos2	0.56	0.8	0.68	2
Gzmb IL-12rb2	0.561	0.8	0.68	2
IL-12rb2 IL-2ra	0.61	0.75	0.68	2
Gzmb Nos2 IL-12rb2 IL-18bp	0.545	0.814	0.679	4
IL-12rb2 IL-2ra IL-18bp	0.596	0.758	0.677	3
Gzmb	0.559	0.791	0.675	1
Nos2 IL-12rb2	0.556	0.767	0.661	2
Nos2 IL-12rb2 IL-18bp	0.553	0.764	0.659	3
IL-12rb2	0.575	0.696	0.636	1
IL-12rb2 IL-18bp	0.553	0.713	0.633	2
Nos2 IL-18bp	0.549	0.71	0.629	2
Nos2	0.564	0.683	0.623	1
IL-18bp	0.529	0.653	0.591	1
