# Supplementary material for: A panel of correlates predicts vaccine-induced protection of rats against respiratory challenge with virulent Francisella tularensis
Source: PLoS One. 2018 May 25;13(5):e0198140. doi: 10.1371/journal.pone.0198140 (PMC5969757; doi:10.1371/journal.pone.0198140)
Supplement: S3 Table — Experimental models consisting of 11 possible variables were analyzed. Shown here are models that have either one explanatory variable (the same name in column and row) or two explanatory variables (different names in the column and the row), for a total of 66 models. Sensitivities and specificity were calculated by discriminating individuals vaccinated with LVS from naïve, as well as from individuals vaccinated with LVS-R or HK-LVS. The corrected classification values is shown, calculated as average of sensitivity and specificity. (PDF) [file pone.0198140.s005.pdf]

S3 Table. Correct Classification for linear discriminant analyses

	CFU	IFN-γ	IL-21	LTA	Gzmb	Nos2	Socs1	IL-12rβ2	IL-2ra	IL-18bp	Tbet
**CFU**	83.1										
**IFN-γ**	90.6	80.3									
**IL-21**	85.0	79.7	71.2								
**LTA**	86.5	80.3	73.2	73.0							
**Gzmb**	82.3	79.5	70.5	74.1	67.5						
**Nos2**	82.6	80.0	71.9	75.0	68.0	62.3					
**Socs1**	92.0	81.9	78.1	77.9	80.1	80.5	75.3				
**IL-12rβ2**	84.8	80.7	70.9	72.4	68.0	66.1	75.5	63.5			
**IL-2ra**	85.0	79.5	70.9	72.2	69.7	70.0	75.9	68.0	68.8		
**IL-18bp**	83.1	79.5	71	71.8	68.6	62.9	76.8	63.3	68.4	59.1	
**Tbet**	90.0	79.7	80.5	78.1	76.5	77.1	78.1	73.7	76.3	78.0	73.8

Experimental models consisting of 11 possible variables were analyzed. Shown here are models that have either one explanatory variable (the same name in column and row) or two explanatory variables (different names in the column and the row), for a total of 66 models. Sensitivities and specificity were calculated by discriminating individuals vaccinated with LVS from naïve, as well as from individuals vaccinated with LVS-R or HK-LVS. The corrected classification values is shown, calculated as average of sensitivity and specificity.
